# Antiviral Effectiveness, Clinical Outcomes, and Artificial Intelligence Imaging Analysis for Hospitalized COVID‐19 Patients Receiving Antivirals

**DOI:** 10.1111/irv.70006

**Published:** 2024-09-16

**Authors:** Yuan Gao, Yixi Dong, Qiushi Bu, Zhijie Gong, Wei Wang, Zhongkai Zhou, Yunyi Gao, Liwei Liu, Menghua Wu, Jiaying Zhang, Lianchun Liang, Hongjun Li, Mengxi Jiang, Zujin Luo, Yingmin Ma, Xinyu Zhang, Zhongjie Hu

**Affiliations:** ^1^ Fourth Department of Liver Disease Center, Beijing You'An Hospital Capital Medical University Beijing China; ^2^ School of Management University of Science and Technology of China Hefei China; ^3^ Academy of Mathematics and Systems Science Chinese Academy of Sciences Beijing China; ^4^ Department of Radiology Beijing Youan Hospital, Capital Medical University Beijing China; ^5^ School of Basic Medicine Qingdao University Qingdao China; ^6^ Department of Urology Beijing You'An Hospital, Capital Medical University Beijing China; ^7^ Department of Infectious Diseases Beijing You'An Hospital, Capital Medical University Beijing China; ^8^ Department of Pharmacology, School of Basic Medical Sciences Capital Medical University Beijing China; ^9^ Department of Respiratory and Critical Care Medicine Beijing Chao‐Yang Hospital, Capital Medical University Beijing China; ^10^ Department of Respiratory and Critical Care Medicine Beijing You'An Hospital, Capital Medical University Beijing China; ^11^ Liver Disease Center Beijing You'An Hospital, Capital Medical University Beijing China

**Keywords:** antiviral, artificial intelligence image, COVID‐19, viral dynamics

## Abstract

**Introduction:**

There is still a lack of clinical evidence comprehensively evaluating the effectiveness of antiviral treatments for COVID‐19 hospitalized patients.

**Methods:**

A retrospective cohort study was conducted at Beijing You'An Hospital, focusing on patients treated with nirmatrelvir/ritonavir or azvudine. The study employed a tripartite analysis—viral dynamics, survival curve analysis, and AI‐based radiological analysis of pulmonary CT images—aiming to assess the severity of pneumonia.

**Results:**

Of 370 patients treated with either nirmatrelvir/ritonavir or azvudine as monotherapy, those in the nirmatrelvir/ritonavir group experienced faster viral clearance than those treated with azvudine (5.4 days vs. 8.4 days, *p* < 0.001). No significant differences were observed in the survival curves between the two drug groups. AI‐based radiological analysis revealed that patients in the nirmatrelvir group had more severe pneumonia conditions (infection ratio is 11.1 vs. 5.35, *p* = 0.007). Patients with an infection ratio higher than 9.2 had nearly three times the mortality rate compared to those with an infection ratio lower than 9.2.

**Conclusions:**

Our study suggests that in real‐world studies regarding hospitalized patients with COVID‐19 pneumonia, the antiviral effect of nirmatrelvir/ritonavir is significantly superior to azvudine, but the choice of antiviral agents is not necessarily linked to clinical outcomes; the severity of pneumonia at admission is the most important factor to determine prognosis. Additionally, our findings indicate that pulmonary AI imaging analysis can be a powerful tool for predicting patient prognosis and guiding clinical decision‐making.

## 1 Introduction

The end of 2022 and the beginning of 2023 marked a pivotal period for China in its battle against the COVID‐19 pandemic, especially concerning the Omicron variant. With the exit from the “zero‐COVID” policy, a vast majority of the population was infected in a remarkably short period, leading to significant medical strain and a considerable number of excess deaths [1, 2]. At that time, the vast majority of patients hospitalized due to COVID‐19 were elderly. In addition to receiving standard care, they were also administered various available antiviral medications. During the initial surge of Omicron infections, nirmatrelvir/ritonavir and azvudine were the sole antiviral medications accessible to patients. The former, however, experienced significant shortages in many Chinese hospitals.

It is noteworthy that there are likely differences in antiviral efficacy between these two drugs. The first real‐world drug comparison study in China indicated that nirmatrelvir/ritonavir could inhibit viral replication more rapidly than azvudine and the time from admission to first SARS‐CoV‐2 negative test is 5.8 days for nirmatrelvir/ritonavir and 10.0 days for azvudine [3]. Subsequently, a series of real‐world studies comparing the two drugs emerged, most of which used mortality as a clinical endpoint or a composite endpoint of mortality and severe disease progression [4, 5, 6, 7, 8]. Meta‐analyses based on real‐world studies have also suggested that patients receiving azvudine may be associated with clinical outcomes improvement, with some analyses indicating that azvudine could be more effective than nirmatrelvir/ritonavir [9, 10, 11]. The majority of these studies showed little difference in mortality rates between the two drugs in real‐world settings, with some suggesting that azvudine might be superior to nirmatrelvir/ritonavir in clinical outcomes [12]. Among these numerous observational studies of hospitalized patients, the use of drugs with greater antiviral efficacy did not seem to translate into substantial clinical benefits. Therefore, it is imperative to explore the reasons for this consequence behind these findings.

In this retrospective cohort study, we conduct a comprehensive analysis of all data from our hospital during the first wave of beginning from late 2022, including viral dynamics, clinical events, and pulmonary imaging analysis, to elucidate the causes behind these observations. To our knowledge, this is the first real‐world study to simultaneously analyze virological features and clinical outcomes and employ artificial intelligence (AI)–assisted imaging to assess the severity of pneumonia in patients receiving antivirals.

## 2 Methods

### 2.1 Study Design and Patients

From November 1, 2022, to March 31, 2023, a period that approximately corresponds to the duration of the first nationwide wave of the COVID‐19 pandemic in China, we conducted a retrospective cohort study at Beijing You'An Hospital. During the study period, nirmatrelvir/ritonavir and azvudine were the only COVID‐19 antiviral drugs available at our hospital. In April 2023, our hospital introduced simnotrelvir/ritonavir, and by the end of 2023, VV116 was also introduced. However, during the study period, no patients received simnotrelvir/ritonavir or VV116. During the initial surge of Omicron variant infection, Beijing You'An Hospital was designated as the highest level COVID‐19 specific hospital in Beijing. This study focuses on the therapeutic outcomes of COVID‐19 treatments. Inclusion criteria were as follows: hospitalized individuals aged 18 years or older, confirmed to have SARS‐CoV‐2 infection by reverse transcription‐polymerase chain reaction (RT‐PCR) testing; those treated with nirmatrelvir/ritonavir or azvudine, in accordance with the standard treatment protocols outlined in “Chinese Diagnosis and Treatment Protocol for COVID‐19 (Trial Version 10)” (Supporting Information). Exclusion criteria were as follows: patients receiving both nirmatrelvir/ritonavir and azvudine; those administered other antiviral or SARS‐CoV‐2 monoclonal antibody therapies; and patients requiring invasive mechanical ventilation at the time of admission. Ethical approval was secured from the Clinical Ethics Committee of Beijing You'An Hospital, affiliated with Capital Medical University (LL‐2023‐105‐K). Anonymity was maintained for all study cases, thus waiving the requirement for informed consent.

### 2.2 Data Source

Utilizing the electronic health records (EHR) from hospitalized patients at Beijing You'An Hospital, Capital Medical University, we accessed the EHRs of all individuals admitted due to COVID‐19. We extracted EHR data relevant to our study from the hospital information system. The extraction process was carried out by experienced IT personnel to ensure the accuracy of data retrieval. During the extraction, we applied multiple filtering criteria to include only data that met the research standards. We performed initial data cleaning on the extracted data, which included removing duplicate records, handling missing values, and correcting data format inconsistencies. The data cleaning process was automated using R to minimize human errors. One of the authors conducted manual validation of the dataset through random sampling to ensure the accuracy of key variables. During data analysis, we also reviewed the EHR for verification in cases of apparent outliers or suspicious anomalies.

These records comprised demographic information, admission dates, discharge status (discharged alive or deceased), previous medical history, records of medication prescribed during hospitalization, laboratory tests, intensive care unit (ICU) admissions, and the results of the initial chest computed tomography (CT) scans among admitted patients (if a CT scan was performed at the outpatient/emergency department prior to admission, this scan was considered the initial one).

### 2.3 Drug Exposure Group

Patients who received at least one dose of oral nirmatrelvir/ritonavir (300 mg/100 mg, twice daily) or at least one dose of oral azvudine (5 mg, once daily) were considered to have treatment exposure and were categorized into the nirmatrelvir/ritonavir group or the azvudine group, respectively. The course of nirmatrelvir/ritonavir is 5 days, and the course of azvudine is 7 days. Hospitalized patients generally have good adherence and can take the medication as prescribed unless death or intubation occurs, leading to treatment termination or inability to continue medication. Patients were included in the analysis if the initial administration of antiviral medication occurred within 48 h after admission.

### 2.4 Primary Measures

In this study, we conducted a tripartite analysis, including viral dynamics, survival curve analysis, and AI‐assisted radiological analysis of chest CT images.

Viral load was measured using the Ct value targeted N‐gene from RT‐PCR tests. A Ct value ≥ 35 was defined as indicative of SARS‐CoV‐2 viral clearance. The time from hospitalization to the first viral clearance was defined as negative RT‐PCR conversion time.

Mortality during hospitalization and ICU admission were considered as a primary composite endpoint. Survival curves were generated based on data provided by EHRs.

The severity of pneumonia in patients who exposed to nirmatrelvir/ritonavir or azvudine was assessed using the first chest CT scan obtained during the current visit (either the first post‐admission chest CT scan or CT scan in outpatient/emergency department). We employed AI‐assisted imaging analysis called VB‐Net (United Imaging Intelligence) on all patients undergoing chest CT scan. This algorithm, which had already been used on more than 6000 multicenter CT scans at the start of our study, enabled the automatic segmentation of infected lung fields, including both left and right lungs, the five lung lobes, and 18 bronchopulmonary segments (Figure S1). The specific details of the initial training, validation, and accuracy metrics of this algorithm are provided in the Supporting Information. We calculated traditional quantitative metrics to quantify the infected areas in each patient's images: (1) the total volume of infection in the lungs (cm^3^), as well as the volume of infection in each lung lobe and bronchopulmonary segment (cm^3^); (2) the percentage of infection in the entire lung, each lung lobe, and each bronchopulmonary segment; and (3) histograms of Hounsfield units (HU) within different infected areas, performing a component analysis using various CT values (HU) within the infected regions [13]. Finally, an infection ratio score was calculated for each chest CT, including a dual analysis for scans with 1 or 5 mm slice thickness.

### 2.5 Covariates

Baseline characteristics for each patient were incorporated into the study as covariates, including age, sex, comorbidities, and the initial N‐gene Ct value (defined as the N‐gene Ct value obtained from RT‐PCR testing within 24 h before or after admission). These covariates were consistent with those included in previous real‐world studies of COVID‐19 antiviral drugs in China [4, 14, 15, 16]. Other laboratory test parameters were also included as part of the baseline data.

### 2.6 Statistical Analysis

We employ propensity score matching (PSM) to balance baseline covariates for all patients who underwent antiviral treatment and received chest CT scans, with a caliper width of 0.2. Covariates for matching included age, sex, COVID‐19 vaccination status, the time from symptom onset to hospitalization, comorbidities, the initial Ct value, and a series of laboratory test parameters. Functional principal component analysis was utilized both pre‐PSM and post‐PSM to model the dynamic Ct values, comparing the intercepts of the fitted curves for different groups at a Ct value of 35. This approach follows the methodology of our previous studies [3, 17]. For all hospitalized patients who underwent chest CT scans, survival analysis was conducted using Kaplan–Meier (KM) curves. The infection ratio before and after matching in the two groups was compared using two independent‐sample *t*‐test.

## 3 Results

**FIGURE 1:**
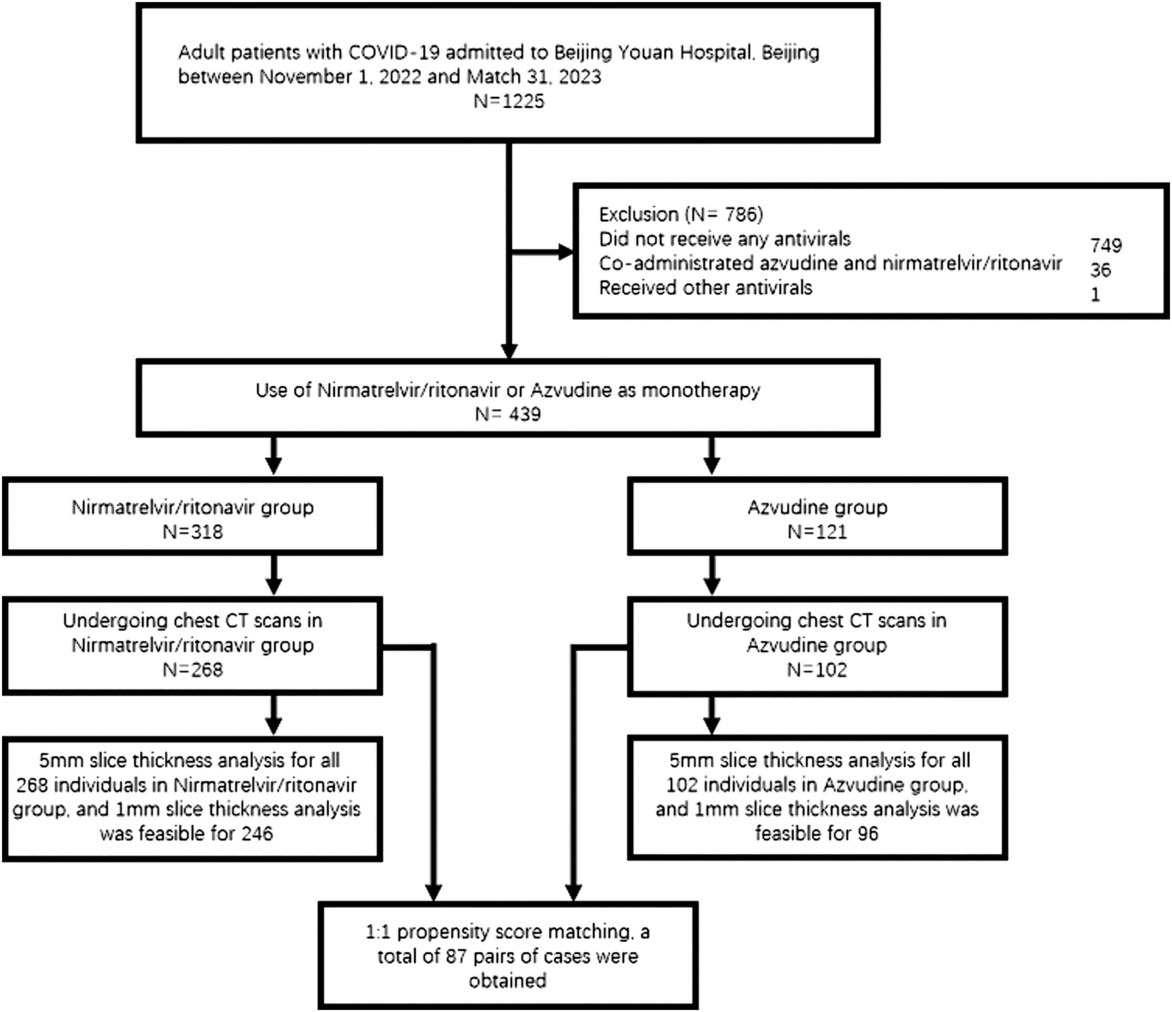
Patient flow diagram.

Between November 1, 2022, and March 31, 2023, a total of 1225 patients were admitted to our hospital due to SARS‐CoV‐2 infection, spanning the period of China's first wave of the Omicron variant surge. Official reports indicate that Omicron subvariants BA.5.2 and BF.7 predominated during the first wave in China [18], resulting in over 80% of the population being infected in a short timeframe. Among these patients, 749 did not receive antiviral treatment, 1 was treated with other antiviral drug, and 36 received combined therapy with nirmatrelvir/ritonavir and azvudine. Of the remaining 439 patients, 318 were treated with nirmatrelvir/ritonavir, and 121 with azvudine, and their clinical features and viral dynamics were placed in supplementary file (Table S1 and Figure S2). Within the cohort treated with nirmatrelvir/ritonavir, 268 underwent chest CT scans in the early stages of the disease (defined as outpatient/emergency visits or within the first week of hospital admission), and among those treated with azvudine, 102 underwent chest CT scans (Figure 1).

In patients who underwent chest CT scans, those who received nirmatrelvir/ritonavir and those who received azvudine showed similar demographic characteristics; however, discrepancies existed in certain features, with a slightly higher incidence of diabetes among patients treated with nirmatrelvir/ritonavir. After PSM was applied to baseline covariates, 87 pairs of patients were formed (Table 1).

**TABLE 1:** Characteristics of hospitalized patients treated with nirmatrelvir or azvudine and underwent chest CT scans.

	Before PSM		After PSM	
	Nirmatrelvir/ritonavir group (*n* = 268)	Azvudine group (*n* = 102)	Nirmatrelvir/ritonavir group (*n* = 87)	Azvudine group (*n* = 87)
Age, years	71.7 (14.0)	70.2 (16.8)	72.2 (13.7)	71.1 (16.8)
Sex				
Female	91 (34%)	43 (42%)	40 (46%)	35 (40%)
Male	177 (66%)	59 (58%)	47 (54%)	52 (60%)
Comorbidities				
Heart diseases	74 (28%)	27 (27%)	24 (28%)	24 (28%)
Cerebrovascular diseases	39 (15%)	20 (20%)	15 (17%)	18 (21%)
Diabetes	79 (30%)	21 (21%)	22 (25%)	20 (23%)
Hypertension	126 (47%)	48 (47%)	43 (49%)	43 (49%)
Gastrointestinal or liver diseases	60 (22%)	19 (19%)	15 (17%)	12 (14%)
Kidney diseases	54 (20%)	22 (22%)	21 (24%)	18 (21%)
History of cancer	45 (17%)	11 (11%)	8 (10%)	9 (10%)
Smoking history	52 (19%)	19 (19%)	16 (18%)	18 (21%)
Alcohol consumption history	35 (13%)	11 (11%)	6 (7%)	8 (9%)
Severity at admission ^a^				
Moderate	129 (48%)	54 (53%)	41 (47%)	43 (49%)
Severe to critical	139 (52%)	48 (47%)	46 (53%)	44 (51%)
Laboratory test				
Alanine aminotransaminase, IU/L	33 (23–50)	29 (20–50)	31 (19–48)	27 (20–46)
Aspartate aminotransferase, IU/L	26 (18–38)	22 (17–35)	23 (18–36)	22 (16–30)
Total bilirubin, mmol/L	10.9 (7.9–15.0)	12.7 (8.2–18.1)	12.4 (8.3–16.0)	12.4 (7.7–16.5)
Creatinine, umol/L	69 (57–85)	73 (62–102)	67 (55–91)	73 (61–101)
Blood urea nitrogen, mmol/L	5.9 (4.2, 8.4)	5.4 (4.0, 8.0)	5.5 (3.9, 8.4)	5.3 (3.9, 8.0)
White blood cell, ×10^9^/L	5.4 (4.3–7.9)	5.9 (4.0–10.2)	5.6 (4.3–8.5)	5.8 (3.9–9.6)
Hemoglobin, g/L	123 (111–136)	123 (108–135)	124 (112–137)	124 (114–136)
Platelets, ×10^9^/L	173 (124–222)	173 (129–241)	187 (145–220)	173 (137–236)
International normalized ratio	1.1 (1.0–1.2)	1.1 (1.1–1.3)	1.1 (1.0–1.2)	1.1 (1.0–1.2)
Procalcitonin, ng/mL	0.1 (0.1–0.3)	0.1 (0.1–0.2)	0.1 (0.1–0.4)	0.1 (0.1–0.2)
C‐reactive protein, mg/L	50.4 (19.1–78.7)	37.2 (13.7–72.6)	54.9 (20.3–80.4)	35.1 (12.5–65.5)
Initial Ct value^b^	31.3 (27.0–35.6)	29.8 (24.6–36.0)	29.4 (24.1–33.1)	29.8 (25.0–34.9)

*Note:* Data are mean (SD), *n* (%), n/N (%), or median (IQR). Data are rounded to the nearest whole number or to one decimal place, as dictated by the specifics of the dataset.

Abbreviation: PSM = propensity score matching.

^a^ The determination of the severity condition is based on the “Chinese Diagnosis and Treatment Protocol for COVID‐19 (Trial Version 10).”

^b^ Ct values exceeding 40 or negative results, are denoted by a Ct value of 40. The initial Ct value refers to the first Ct value recorded after admission, although this measurement is not necessarily obtained from tests conducted on the first day of hospitalisation.

**FIGURE 2:**
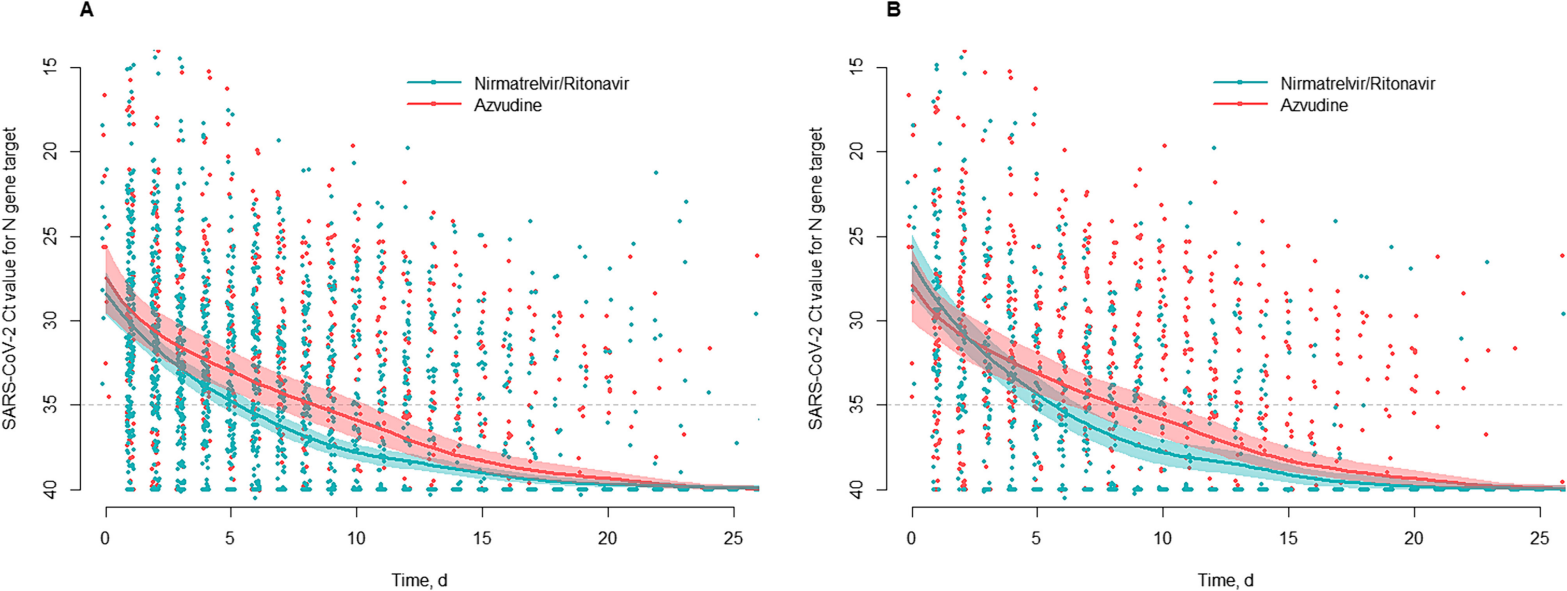
Scatter plot of serial cycle threshold values for patients undergoing chest CT scans. Cycle threshold (Ct) values obtained from qRT‐PCR tests targeting N genes in infected individuals are depicted in the figure. Blue and red circles represent patients treated with nirmatrelvir/ritonavir and azvudine, respectively. The solid lines show the average trend based on functional principal components analysis, with shaded regions illustrating 95% credible intervals for these trends. A dashed line marks the Ct threshold of 35, and any Ct values above 40 or indicating negative results are denoted by a Ct value of 40 for uniformity in representation. (A) 2214 samples collected from subjects before propensity score matching. (B) 1202 samples collected from 87 propensity score matched pairs.

Before PSM, patients receiving nirmatrelvir/ritonavir achieved viral clearance in a median of 5.4 days (95% CI 4.8–6.3) compared to 8.4 days (95% CI 6.6–10.8) for the azvudine group (*p* < 0.001) (Figure 2A). After PSM, patients treated with nirmatrelvir/ritonavir still demonstrate faster viral clearance compared to those receiving azvudine (5.7 days vs. 8.4 days) (*p* = 0.002) (Figure 2B). We also plotted KM curves for the two groups based on the time to first negative test result, which similarly indicated that nirmatrelvir/ritonavir was superior to azvudine (Figure S3). This finding is consistent with conclusions from our previous research [3].

**FIGURE 3:**
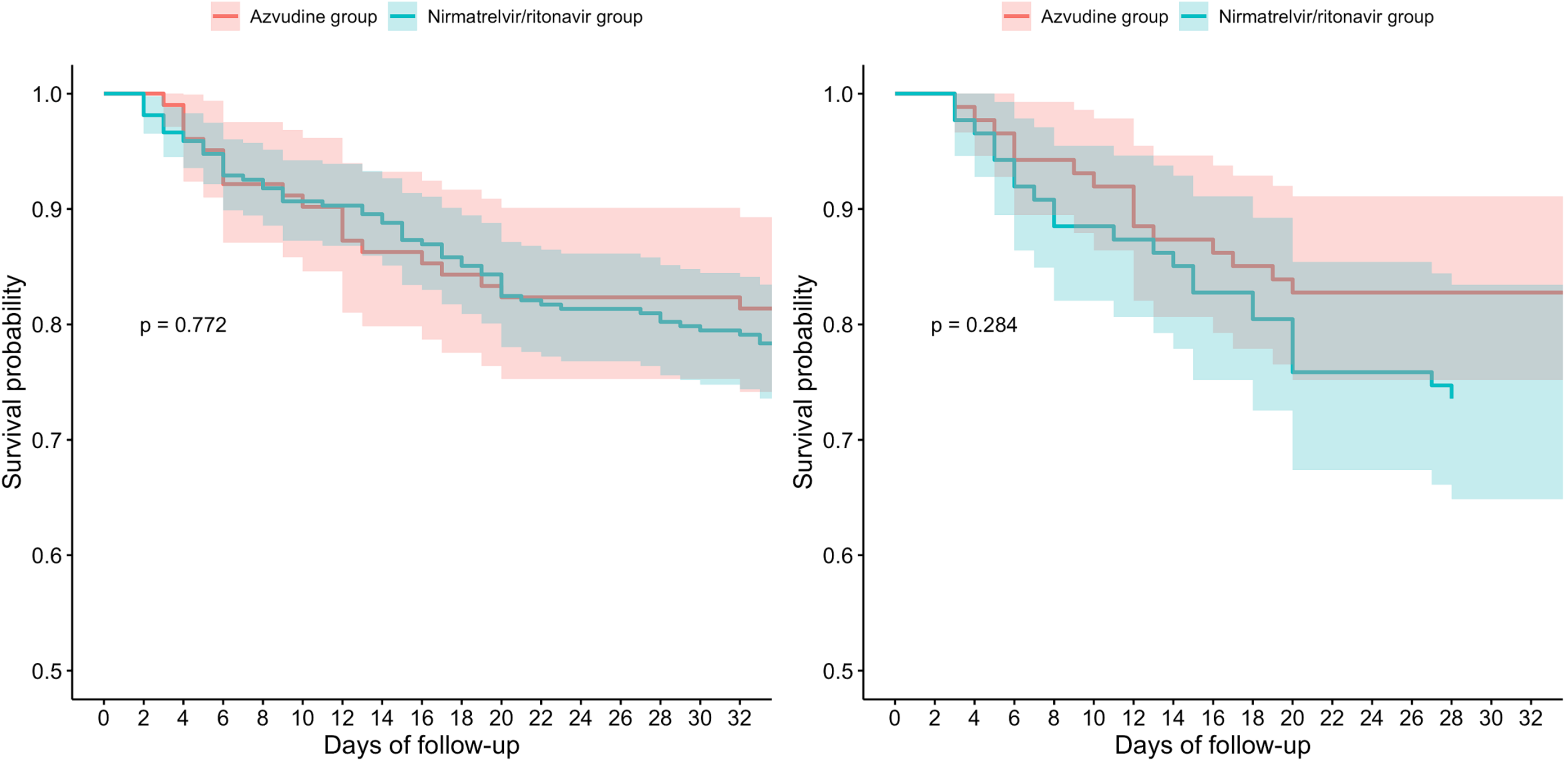
Kaplan–Meier time‐to‐event curves for patients undergoing chest CT scans. (A) Before propensity score matching, composite outcomes of patients receiving nirmatrelvir/ritonavir or azvudine. (B) After propensity score matching, composite outcomes of patients receiving nirmatrelvir/ritonavir or azvudine.

KM survival plots, based on information from EHRs and utilizing the log‐rank test to compare outcome events between patients receiving different antivirals, showed no significant differences between two groups (*p* = 0.772) (Figure 3A). After PSM, no differences were still observed in the survival curves between patients treated with nirmatrelvir/ritonavir and those treated with azvudine (*p* = 0.284) (Figure 3B). We performed a stratified analysis of the impact of different baseline characteristics on patient prognosis, with data provided in the appendix (Table S2). Additionally, detailed information on follow‐up times and the number of patients triggering endpoint events in each group is provided in the appendix (Table S3).

**FIGURE 4:**
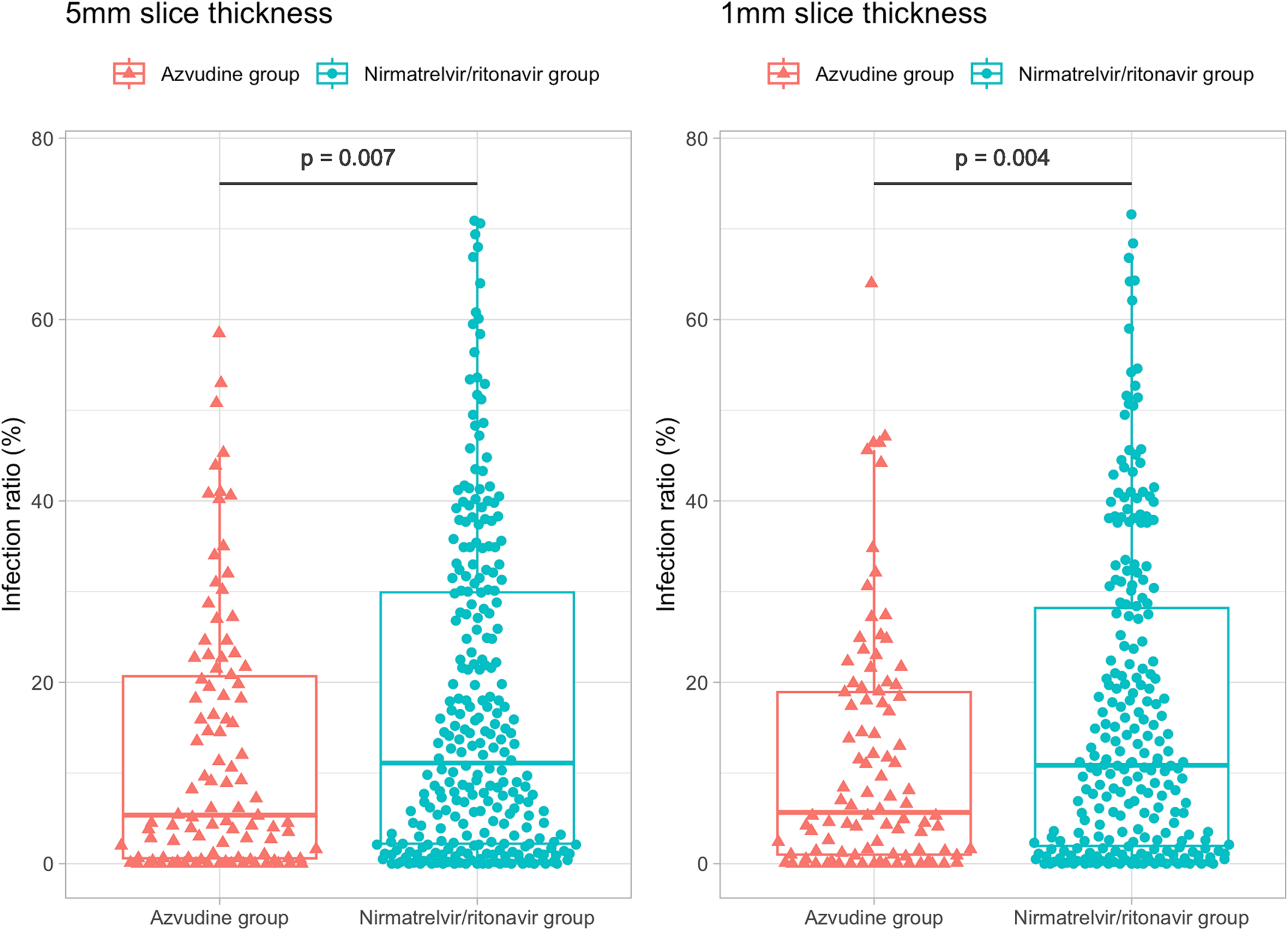
COVID‐19 pneumonia condition assessed by artificial intelligence imaging analysis. Each red triangle represents the lung infection ratio of patients treated with azvudine, and each blue circle represents the lung infection ratio of patients treated with nirmatrelvir/ritonavir. (A) The analysis with a slice thickness of 5 mm included a total of 370 patients (268 in the nirmatrelvir/ritonavir group and 102 in the azvudine group). (B) Among these, 342 could be analyzed with a slice thickness of 1 mm (246 in the nirmatrelvir/ritonavir group and 96 in the azvudine group).

AI‐assisted chest CT analysis suggested a significantly higher infection ratio in patients treated with nirmatrelvir/ritonavir compared to those receiving azvudine (Data S1, Data S2). AI‐based radiological analysis with a 5 mm slice thickness revealed a higher pneumonia infection ratio in the nirmatrelvir/ritonavir group (median 11.1%, IQR 2.2–29.93) compared to the azvudine group (median 5.35%, IQR 0.6–20.68) with *p* value of 0.007 (Figure 4A), in the analysis with a 1 mm slice thickness, infection ratio is 10.85 [IQR 1.95–28.20] for patients receiving nirmatrelvir/ritonavir, and 5.65 [IQR 1.0–18.93] for patients receiving azvudine (*p* = 0.004) (Figure 4B). This trend was consistent across cohorts both pre‐PSM and post‐PSM (Figure S4).

**FIGURE 5:**
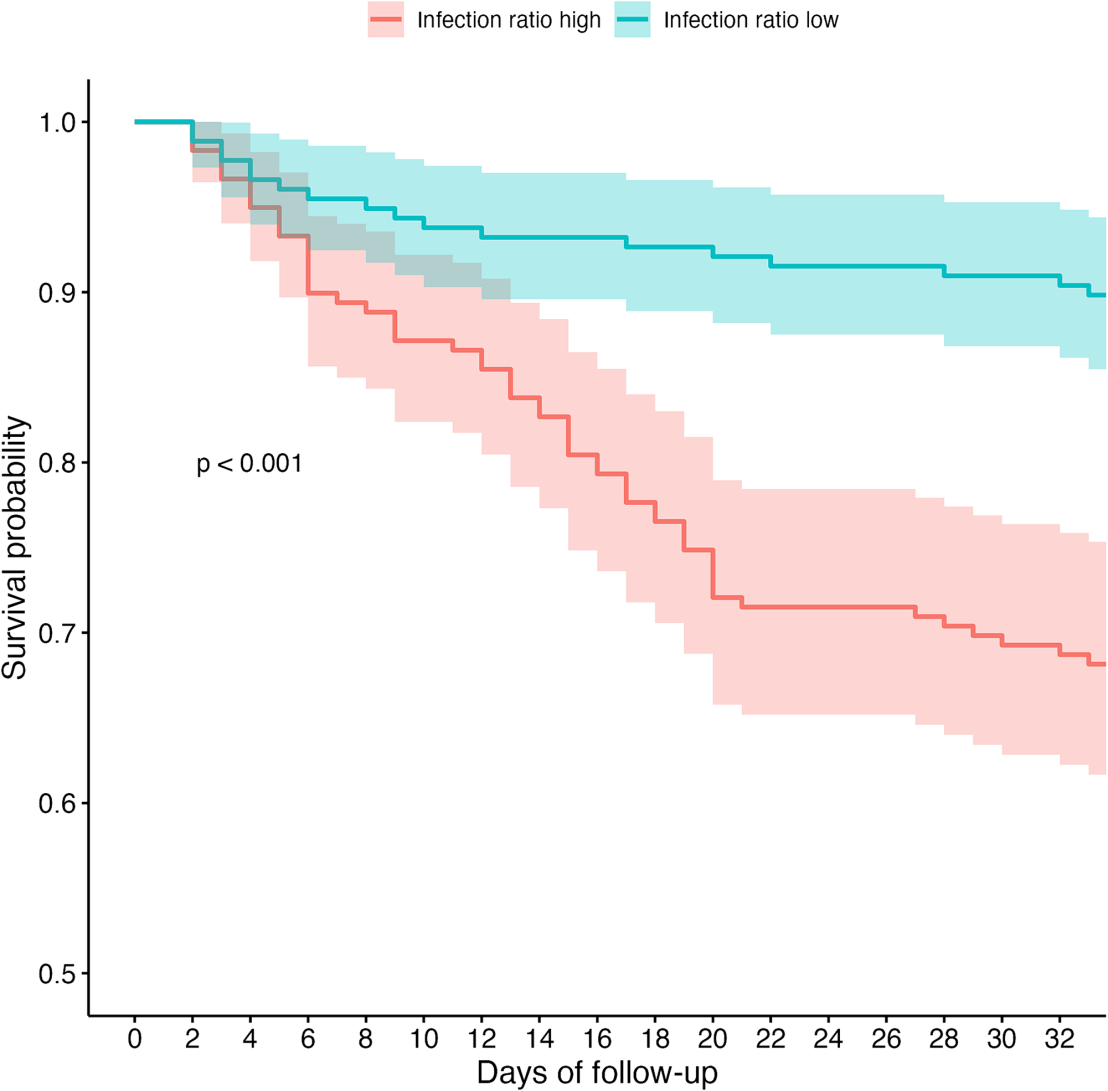
Kaplan–Meier time‐to‐event curves for patients grouping by the median infection ratio of 9.2.

After including the infection ratio as a covariate in the propensity score matching, we generated KM curves for the two drug groups. The survival curves for the nirmatrelvir/ritonavir and azvudine groups still did not show significant differences (Figure S5). Additionally, we used the median infection ratio of 9.2 to divide all patients into two groups and generated KM curves for each group separately (Figure 5). As shown, the rate of mortality during hospitalization or ICU admission for the population with an infection ratio greater than 9.2 was nearly three times that of the population with an infection ratio less than 9.2. We also plotted KM curves for the two drug groups after stratifying by infection ratio. Among patients with an infection ratio lower than 9.2, the mortality rate in the nirmatrelvir/ritonavir group was lower than in the azvudine group, but it did not reach statistical significance due to wide confidence intervals. In patients with an infection ratio higher than 9.2, no survival difference was observed between the two antiviral drug groups (Figure S6).

## 4 Discussion

In our study, among COVID‐19 patients who underwent chest CT scans, negative RT‐PCR conversion time was significantly faster in patients treated with nirmatrelvir/ritonavir compared to those treated with azvudine. Our first finding reconfirmed that the time to viral clearance was significantly shorter in the nirmatrelvir/ritonavir group than in the azvudine group. Our second finding was that the infection ratio scores in patients exposed to nirmatrelvir/ritonavir were significantly higher than those in patients exposed to azvudine, consistently observed in both pre‐PSM and post‐PSM cohorts.

To understand the phenomenon observed in our study, it is necessary to revisit the winter of 2022 in China, a critical phase in the pandemic was characterized by a rampant surge in Omicron variant infections. During this time, a pronounced imbalance emerged in the availability of antiviral agents, with hospitals across the nation grappling with a significant scarcity of nirmatrelvir/ritonavir, in stark contrast to the relatively steady availability of azvudine. Faced with this dilemma, medical professionals were often compelled to prioritize administering antiviral medications with the strongest evidence base to patients with more severe pneumonia. This strategic allocation led to an interesting phenomenon: Despite the severe shortage of nirmatrelvir/ritonavir, its use in hospitalized patients still surpassed that of azvudine, as evidenced by multiple real‐world studies [4, 5, 6, 12].

A prevalent method to address disparities of baseline characteristics in real‐world studies is PSM, which aims to equalize baseline covariates across study groups. However, PSM primarily relies on accessible data, which may not fully represent the differences in patient histories or conditions not explicitly accounted for in the model [19, 20]. Our research suggests that employing standard clinical features as covariates for PSM may not truly balance baseline characteristics because of the lack of quantitative assessment of pneumonia.

Our third finding is that when AI imaging analysis is introduced, the mortality rate for patients with an infection ratio higher than 9.2 is three times that of patients with an infection ratio lower than 9.2. This suggests that the severity of pneumonia at admission is the most important indicator determining patient prognosis, but the impact of baseline comorbidities on prognosis is very limited. Our results also suggest that in patients with COVID‐19 pneumonia, potent antiviral treatment does not lead to improved prognosis, which is consistent with previous study findings [21]. This could be attributed to the fact that, in the later stages of the disease, once the virus has caused substantial lung injury, the clinical benefits by the inhibition of SARS‐CoV‐2 are relatively limited. It is important to note that our study's subjects were primarily elderly patients with an impaired viral clearance capacity compared to the younger population [17]. Even with antiviral treatment, with a considerable proportion of individuals still testing positive for SARS‐CoV‐2 after 5 days of hospitalization, this study does not address whether an intervention that more rapidly inhibits viral replication than the above two agents could improve prognosis, which may require further research.

Our research is subject to certain limitations. Firstly, the follow‐up period for our study is relatively short, ending at the point of either patients discharge or death. Secondly, the application of AI in imaging analysis, though innovative, demands significant computational power and extended processing times compared to conventional medical reports, and these requirements pose substantial barriers to widespread adoption in current hospital settings. Thirdly, in this study, the confidence intervals for survival curves in patients with a low infection ratio are too wide. We cannot exclude the possibility that potent antiviral treatment may improve the prognosis of patients with milder pneumonia. Lastly, this is the first use of the VB‐Net tool to quantify clinical images of lung CT scans and to apply this quantification to determine clinical endpoints. Given the limited sample size of this study, further verification with more data is needed in the future.

It is the first time to integrate analysis of viral dynamics, clinical endpoints, and AI‐assisted imaging analysis to examine multiple characteristics of COVID‐19 patients undergoing antiviral therapy in one study. Our study suggests that in real‐world studies regarding hospitalized patients with COVID‐19 pneumonia, the antiviral effect of nirmatrelvir/ritonavir is significantly superior to azvudine, but the choice of antiviral agents is not necessarily linked to clinical outcomes; the severity of pneumonia at admission is the most important factor determining prognosis. Additionally, our findings indicate that pulmonary AI imaging analysis can be a powerful tool for predicting patient prognosis and guiding clinical decision‐making.

## Author Contributions

Conceptualization: Yuan Gao, Yixi Dong, Qiushi Bu, Zhijie Gong, Yingmin Ma, Xinyu Zhang, and Zhongjie Hu. Writing – original draft: Yuan Gao, Yixi Dong, Qiushi Bu, Zhijie Gong, Wei Wang, and Zhongkai Zhou. Implementation of the computer code: Yixi Dong, Qiushi Bu, and Zhijie Gong. Data collection and validation: Yunyi Gao, Liwei Liu, and Menghua Wu. Visualization: Yixi Dong and Qiushi Bu. Writing – review and editing: Jiaying Zhang, Lianchun Liang, Mengxi Jiang, and Zujin Luo. AI image analysis processing: Wei Wang, Zhongkai Zhou, and Hongjun Li. Interpretation of data: All authors. Supervision: Yingmin Ma, Xinyu Zhang, and Zhongji Hu.

## Ethics Statement

The ethics of this study have been approved by the Ethics Committee of Beijing You'An Hospital (Approval No: LL‐2023‐105‐K).

## Conflicts of Interest

Yuan Gao is a medical consultant for Sinovac. He declares receiving consulting fees from Sinovac and receiving lecture fees from Pfizer over the past 36 months. The other authors declare no conflicts of interest.

### Peer Review

The peer review history for this article is available at https://www.webofscience.com/api/gateway/wos/peer‐review/10.1111/irv.70006.

## Supporting information


**Data S1.** Supporting Information.

**Data S2.** Supporting Information.

**Table S1.** Characteristics of hospitalized patients receiving nirmatrelvir/ritonavir or receiving azvudine.
**Table S2.** Stratified analysis of the impact of different baseline characteristics on patient prognosis.
**Table S3.** Analysis of follow‐up durations and number of patients triggering endpoint events in the two groups.
**Figure S1.** Visualization of artificial intelligence imaging analysis results for chest ct scans based on VB‐Net (United Imaging Intelligence).
**Figure S2.** Scatter plot of serial cycle threshold values for all patients treated with nirmatrelvir/ritonavir and azvudine (including patients did not undergo chest CT scans).
**Figure S3.** KM curves for the two groups based on the time to first negative test result.
**Figure S4.** AI‐based radiological analysis in hospitalized patients treated with nirmatrelvir/ritonavir or azvudine after propensity score matching.
**Figure S5.** KM curves for nirmatrelvir/ritonavir and azvudine after infection ratio incorporate into covariates of propensity score matching.
**Figure S6.** KM curves for nirmatrelvir/ritonavir and azvudine in patients with infection ratio high (> 9.2) and infection ratio low (≤ 9.2).
**Figure S7.** Scatter plot of serial cycle threshold values for all patients with infection ratio high (> 9.2) and infection ratio low (≤ 9.2).

## Data Availability

All data generated or analyzed during this study are included in this published article and its supplementary information files.
