# Supplementary material for: Antiviral Effectiveness, Clinical Outcomes, and Artificial Intelligence Imaging Analysis for Hospitalized COVID‐19 Patients Receiving Antivirals
Source: Influenza Other Respir Viruses. 2024 Sep 16;18(9):e70006. doi: 10.1111/irv.70006 (PMC11405122; doi:10.1111/irv.70006)
Supplement: Supplementary file 1 — Data S1. Supporting Information. [file IRV-18-e70006-s003.pdf]

医保卡号	treatment	Volume.of.whole.lung.cm..	Volume.of.left.lung.cm..	Volume.of.right.lung.cm..	Infected.Volume.of.whole.lung.cm..	Infection.ratio.of.whole.lung..	Infected.Volume.of.left.lung.cm..	Infected.Volume.of.right.lung.cm..	Infected.Volume.of.left.upper.lobe.cm..	Infected.Volume.of.left.lower.lobe.cm..	Infected.Volume.of.right.upper.lobe.cm..	Infected.Volume.of.right.middle.lobe.cm..	Infected.Volume.of.right.lower.lobe.cm..	Infected.Volume.of.Left.Upper.Lobe.Apicoposterior.Segment.cm..	Infected.Volume.of.Left.Upper.Lobe.Anterior.Segment.cm..	Infected.Volume.of.Left.Upper.Lobe.Superior.lingular.Segment.cm..	Infected.Volume.of.Left.Upper.Lobe.Inferior.lingular.Segment.cm..	Infected.Volume.of.Left.Lower.Lobe.Superior.Segment.cm..	Infected.Volume.of.Left.Lower.Lobe.Anteromedial.basal.Segment.cm..	Infected.Volume.of.Left.Lower.Lobe.Lateral.basal.Segment.cm..	Infected.Volume.of.Left.Lower.Lobe.Posterior.basal.Segment.cm..	Infected.Volume.of.Right.Upper.Lobe.Apical.Segment.cm..	Infected.Volume.of.Right.Upper.Lobe.Posterior.Segment.cm..	Infected.Volume.of.Right.Upper.Lobe.Anterior.Segment.cm..	Infected.Volume.of.Right.Middle.Lobe.Lateral.Segment.cm..	Infected.Volume.of.Right.Middle.Lobe.Medial.Segment.cm..	Infected.Volume.of.Right.Lower.Lobe.Superior.Segment.cm..	Infected.Volume.of.Right.Lower.Lobe.Medial.basal.Segment.cm..	Infected.Volume.of.Right.Lower.Lobe.Anterior.basal.Segment.cm..	Infected.Volume.of.Right.Lower.Lobe.Lateral.basal.Segment.cm..	Infected.Volume.of.Right.Lower.Lobe.Posterior.basal.Segment.cm..	Infected.Volume.of.HU....750..cm..	Infected.Volume.of.HU..750..300..cm..	Infected.Volume.of.HU..300.49..cm..	Infected.Volume.of.HU.50...cm..
0000014232	Paxlovid group	3527.4	1474.1	2053.3	10.2	0.3	8.5	1.6	0.3	8.2	0.9	0	0.8	0	0	0.2	0.2	0	4.3	2.7	1.2	0	0	0.9	0	0	0.3	0	0	0	0.5	3.9	4.5	1.4	0.3
0000014986	Paxlovid group	4884.1	3175	1709.1	127.2	2.6	4.9	122.3	2.3	2.6	4.2	9.1	109	0	0	0.8	1.5	0	0	1	1.6	0.5	2.5	1.2	9.1	0	29.8	0	6.3	44	28.9	9.8	35.8	66	15.7
0000014986	Paxlovid group	4884.1	3175	1709.1	127.2	2.6	4.9	122.3	2.3	2.6	4.2	9.1	109	0	0	0.8	1.5	0	0	1	1.6	0.5	2.5	1.2	9.1	0	29.8	0	6.3	44	28.9	9.8	35.8	66	15.7
0000017375	Paxlovid group	3480.6	1653.4	1827.2	672.6	19.3	327.8	344.7	80.3	247.6	114.8	22.3	207.6	6.4	14.2	34.2	25.6	94.8	21.3	61.9	69.5	30.1	61.3	23.4	10.9	11.4	75	25.8	36.8	26.8	43.3	129.4	372	137.6	33.6
0000030715	Paxlovid group	4007.9	1736	2271.9	91.7	2.3	66.7	25	5.8	60.9	8.7	3.5	12.8	0	0.1	0.2	5.5	35.6	0.5	4.6	20.2	0	8.7	0	0	3.5	9.7	0.2	0	1	1.8	35.4	42.5	10.1	3.7
0000061646	Paxlovid group	3091.4	1411.3	1680.1	319.1	10.3	116.6	202.5	1.2	115.4	7.3	67.1	128.1	0.3	0	0.3	0.6	13.6	34.8	39.5	27.5	0.2	5	2.1	29.7	37.5	22.8	21.3	9.8	13.6	60.6	65.4	188.3	57.6	7.8
0000064567	Paxlovid group	2231.6	1028.8	1202.8	698	31.3	375.1	322.9	146.8	228.2	96.5	48.8	177.6	61.1	55	22.8	7.8	44.2	46.8	79.2	58.1	0.2	67.1	29.2	30	18.7	39.1	13.8	9.9	63.9	51	16.3	474.8	197.5	9.3
0000086612	Paxlovid group	2965.2	1350.9	1614.3	2029.5	68.4	904.1	1125.4	502.7	401.4	534.5	155.9	435	171.4	103.4	113.4	114.5	66.5	83.1	122.8	129	146.6	173.3	214.6	69.7	86.2	107	53.4	83.5	115.6	75.4	87.2	1383.9	543.1	15.3
0000108840	Paxlovid group	4458.3	2476.2	1982.1	441.2	9.9	235.9	205.2	65.1	170.8	31.5	30	143.7	5.1	11.5	32.9	15.6	72.8	40	17.1	41	3.9	12.6	15.1	19.5	10.5	21.6	0.5	33.1	59.3	29.1	119.9	221.7	73	26.5
0000121825	Paxlovid group	5492.9	2589.9	2902.9	2105.4	38.3	785.9	1319.4	272.8	513.1	527.2	122.6	669.7	131.5	3.1	40.9	97.4	64.5	239.7	88.8	120.1	129.3	233.1	164.8	93.5	29	170.8	50.9	125.5	145.3	177.1	589.7	1387.3	102.4	25.9
0000146688	Paxlovid group	4545.6	2119.5	2426.1	256.3	5.6	22.6	233.7	2.5	20.2	52	28.7	153	1.2	0.5	0.2	0.5	1.1	5.1	9.3	4.7	0.3	34.4	17.3	25.3	3.3	29.5	0	32.3	59.9	31.3	64.8	138.2	46.6	6.7
0000150781	Paxlovid group	3144.6	1364.6	1780.1	592.2	18.8	357.7	234.4	200.3	157.4	113.2	20.4	100.9	104.3	25.3	37.4	33.4	83.9	17	45	11.5	26.9	40.7	45.6	16.8	3.5	3.1	2	2.4	53.7	39.6	84	308.9	162.2	37.1
0000158011	Paxlovid group	2364.7	1071.5	1293.2	11.7	0.5	0.3	11.4	0.3	0	0	0.5	10.8	0.3	0	0	0	0	0	0	0	0	0	0	0	0.5	3.7	0	0	3.6	3.6	0.2	10.7	0.8	0
0000184792	Paxlovid group	4361.6	1949	2412.6	207.2	4.8	105.6	101.7	73.3	32.3	39.3	5.9	56.5	17.7	11.1	28.3	16.2	0	16.7	6.7	8.8	10.2	17.6	11.5	5.3	0.6	19.4	1.7	14.8	11.8	8.9	12.2	159.6	34.5	0.9
0000190362	Paxlovid group	3294.7	1580.7	1714	1350.6	41	654.1	696.5	252	402.1	208.5	95.1	392.8	54.2	76.4	78.8	42.6	107.3	91.3	83.3	120.2	42.3	30	136.3	54.5	40.7	97.1	17.1	108.2	90.8	79.6	161.8	1029.8	155.6	3.3
0000221283	Azvudine group	3440.6	1553.2	1887.4	153	4.4	23.3	129.6	3.8	19.5	9.5	0.4	119.8	0	0.1	3.5	0.3	0	0.2	5.4	13.9	1	8.3	0.2	0.2	0.1	29.4	0.4	16.2	39.7	34	82.9	63.4	5.4	1.3
0000246981	Paxlovid group	6086.4	2739.7	3346.7	0	0	0	0	0	0	0	0	0	0	0	0	0	0	0	0	0	0	0	0	0	0	0	0	0	0	0	0	0	0	0
0000280303	Paxlovid group	6115.5	2823.9	3291.6	0	0	0	0	0	0	0	0	0	0	0	0	0	0	0	0	0	0	0	0	0	0	0	0	0	0	0	0	0	0	0
0000291750	Azvudine group	2839.2	1317	1522.3	0	0	0	0	0	0	0	0	0	0	0	0	0	0	0	0	0	0	0	0	0	0	0	0	0	0	0	0	0	0	0
0000296831	Paxlovid group	2406.1	1027	1379.2	213.9	8.9	127.2	86.7	8.1	119	7.3	0.8	78.6	3.9	1.2	0	3.1	15.6	36.7	47.6	19.2	1.9	5.4	0	0.8	0	22.2	3.1	10.8	19.2	23.3	24.2	152.8	34	2.9
0000306054	Paxlovid group	4840.7	2239.5	2601.2	788.3	16.3	303.7	484.6	189.1	114.6	166	30.5	288.1	84.2	25.4	37.1	42.5	7.1	27.1	67.3	13.1	7.4	114.2	44.4	9.4	21.1	148	3.7	9.2	67.6	59.6	35.6	589.4	160.1	3.2
0000307654	Azvudine group	3865.8	1596.9	2268.9	326.5	8.4	152.6	174	90.2	62.3	29.1	10.4	134.5	6	0.7	22.7	60.8	1.3	23.3	7.3	30.4	0	7.5	21.6	2.3	8.2	42	9	12.1	30.7	40.7	37.4	266.8	21.8	0.6
0000308605	Paxlovid group	2431.6	1115	1316.7	121.4	5	33.9	87.6	2.1	31.8	8.8	17.9	60.8	2.1	0	0	0	4.4	2.6	12.9	11.9	3.6	4	1.2	11.8	6.1	14.7	4.7	1.7	23.5	16.2	2.6	60.1	49.5	9.3
0000325887	Azvudine group	4506	2036.4	2469.6	74.1	1.6	15.5	58.6	6.6	8.9	12.8	0	45.8	1.3	2.1	0.8	2.4	7.8	0	0.4	0.7	0.4	12.3	0	0	0	22.3	0.5	0.1	10.7	12.2	7.4	58.1	8.3	0.3
0000325980	Paxlovid group	3978.4	1929.7	2048.7	57	1.4	35.9	21	8.6	27.3	2.6	0	18.5	6.8	0	0	1.8	1.6	12.1	8.2	5.4	0	2.6	0	0	0	13.5	0	0.6	0.6	3.8	25.2	22.2	7.4	2.3
0000337873	Paxlovid group	3806.1	1645.2	2160.9	1541.9	40.5	830.1	711.8	290	540.1	153.9	75.5	482.5	43.5	78.8	49.3	118.5	25.6	149.4	198.3	166.8	4.6	49.5	99.8	30.3	45.2	136.7	15	127.5	149.1	54.1	91.6	783.8	613.7	52.9
0000341557	Paxlovid group	3856.5	1500.2	2356.2	477.3	12.4	465.9	11.4	426.6	39.3	1.1	7.2	3.1	92.4	120	116	98.2	19.5	0.4	3.6	15.7	0	0	1.1	2.3	5	1.9	0.1	0.1	1.1	0	29.6	293.8	142.9	11
0000370702	Paxlovid group	3941.6	1729.2	2212.4	35.3	0.9	6.8	28.5	0.4	6.4	8.2	0.1	20.2	0.3	0	0	0.2	4.8	0	0	1.6	0.1	8.1	0	0.1	0	1.7	4.8	11.5	1.9	0.4	3.7	18.8	10.8	2
1000057764	Paxlovid group	4165.3	1948.2	2217.1	1199.5	28.8	247.4	952.1	122.4	125	478.2	133	340.8	82.7	6.9	12.5	20.3	11.4	11.6	50.3	51.7	113.9	187.2	177.2	102.1	31	113.8	7.6	77.4	80.3	61.7	226.4	570.8	311	91.3
1000091615	Paxlovid group	1917.2	1023.3	893.9	9.9	0.5	0	9.9	0	0	0	0.4	9.5	0	0	0	0	0	0	0	0	0	0	0	0	0.4	0.5	2.4	0	3	3.6	0.2	5.1	3.9	0.6
1000098957	Paxlovid group	3441.7	1727.1	1714.5	155.8	4.5	26	129.7	14.4	11.6	3.2	0	126.6	6.6	0	0.2	7.6	0.9	0.5	5.1	5.1	0	3.2	0	0	0	66.5	12.6	9.2	27.3	10.9	3	121.7	28.6	2.5
1000102044	Paxlovid group	4928.3	2083	2845.3	520.5	10.6	123.8	396.7	48.6	75.2	165.4	46.1	185.2	2.5	26.1	15.5	4.5	9.8	21.1	16.7	27.5	61.3	85.3	18.9	25.1	21	54.3	1.3	29.5	69.9	30.2	139.4	356.4	23.9	0.8
1000108172	Paxlovid group	3474.3	1587.2	1887	0	0	0	0	0	0	0	0	0	0	0	0	0	0	0	0	0	0	0	0	0	0	0	0	0	0	0	0	0	0	0
1000121884	Paxlovid group	3837.1	1402.1	2434.9	315.5	8.2	86.8	228.7	3.3	83.5	41.5	9.6	177.6	3.3	0	0	0	2.2	10.6	37.1	33.6	9.2	24.3	8	7.8	1.8	39.7	7.3	31.2	29.6	69.8	78	171.6	57.8	8.1

1000130965	Paxlovid group	4142.4	1850.9	2291.5	437.7	10.6	179.6	258.1	24.7	154.9	114.8	12.9	130.4	11.5	0.7	6.7	5.7	14.4	31.5	70.5	38.5	2.1	100.2	12.6	12.4	0.6	61.8	0.9	9	42.8	15.9	147.2	239.4	42	9.1
1000135966	Azvudine group	3383.6	1610.4	1773.1	852.1	25.2	404.3	447.8	207.7	196.7	100.4	114	233.3	74.4	59.8	52.1	21.4	27.1	60.3	54.4	54.8	25.4	46.5	28.5	70.8	43.2	79.7	23.3	50.2	49.8	30.3	72.6	424.1	309.6	45.8
1000169129	Paxlovid group	3310	1299.6	2010.5	913.4	27.6	375.2	538.2	139.5	235.7	107.9	17.4	412.9	66.4	4.9	25.9	42.3	43.5	44.8	101.1	46.3	10.5	97.3	0.2	4.5	12.9	102.6	20.2	96.3	121.6	72.1	62.8	481.1	326.4	43.2
1000255169	Paxlovid group	4783	1966.7	2816.3	1906.4	39.9	907.6	998.9	520.1	387.5	420.2	106.9	471.7	96	79.7	198.6	145.8	43.1	112.7	112.3	119.4	128.4	84.6	207.3	51.3	55.6	32.1	52.3	160.1	178.3	48.9	118.1	934	742.2	112.1
1000273005	Paxlovid group	5512.1	2604.9	2907.1	292	5.3	70.8	221.2	0.5	70.3	28	50.6	142.7	0.1	0.4	0	0	1.9	0.2	3.2	65	1.7	8.7	17.5	2.5	48	88.5	1	1.7	27.6	23.9	171.5	100.3	17.8	2.4
1000281522	Paxlovid group	2451.7	1037.1	1414.7	425.3	17.3	131.6	293.7	53.7	78	105.4	29.5	158.8	26.6	8.1	14.9	4	22.4	21.6	31.9	2.1	20.2	59.7	25.5	26.3	3.2	71.5	2.8	44.1	25.8	14.6	14	300.4	107.2	3.7
1000301548	Paxlovid group	5167.1	2364	2803.1	68.7	1.3	31.5	37.2	10.4	21	11.9	5.8	19.5	3.8	0.8	4.2	1.7	2.8	2.9	11	4.3	2	0.9	9	1.4	4.4	1.4	1.1	2.8	13.6	0.5	12.4	41.9	12.1	2.3
1000303107	Paxlovid group	1797.5	803.9	993.6	681.8	37.9	388.2	293.6	166.1	222.1	79.4	38.7	175.5	75.6	26	35.6	29	42.9	56.3	72.7	50.3	11.2	13.8	54.4	20.3	18.4	46.1	7.3	44.4	53.9	23.7	72.2	384.9	189.8	34.9
1000316781	Paxlovid group	5826.8	2713.9	3112.8	40.5	0.7	7.8	32.6	1.2	6.6	9	13.4	10.2	0.1	0	0	1.1	0	0.4	5.5	0.7	5.7	2.7	0.6	7.2	6.2	0.1	1.7	2.3	3.9	2.3	10.4	18.9	9.1	2.1
1000321166	Paxlovid group	3831.4	1284.2	2547.2	1097.9	28.7	796.7	301.2	414.3	382.4	94.3	9.1	197.8	153.4	103	95.5	62.4	104.1	63	106.7	108.6	31.5	53.3	9.6	4.5	4.6	31.6	19	34.9	44.2	68	190.9	488.3	345.3	73.5
1000321265	Paxlovid group	5842.8	2745.5	3097.3	95.1	1.6	30.5	64.7	12.7	17.8	14.9	0.5	49.2	7.7	0.1	0.1	4.7	5.2	4.6	2.7	5.3	0.9	13.6	0.4	0.3	0.2	25.3	0	1.8	12.6	9.6	16	50.7	27	1.4
1000357266	Paxlovid group	3754.1	1691	2063.1	132.5	3.5	43.4	89.1	1	42.4	2.5	3.9	82.7	1	0	0	0	1	4	18	19.4	0.9	0.2	1.4	0.4	3.5	4.4	2.4	8.5	39.3	28.2	5.1	84	42.5	0.8
1000383224	Paxlovid group	2486.4	1344.9	1141.4	937.5	37.7	341.8	595.7	104	237.8	210.8	125.1	259.8	35.9	6.4	18.9	42.9	37.8	34.9	89.2	75.8	31	97.4	82.4	55.2	69.9	65.3	10.8	54.2	87.7	41.9	123.8	555.5	218.7	39.6
1000452970	Azvudine group	2157.4	1054.9	1102.5	1001.3	46.4	417.2	584.1	213.1	204.2	251.3	72.6	260.2	85.7	27.4	51.9	48.1	41	48.1	67.8	47.2	99.8	97.2	54.4	28.3	44.3	87.9	24.4	67.8	59.8	20.2	33	592.7	361	14.7
1000454501	Paxlovid group	4035.7	2594.7	1441	10.4	0.3	0	10.4	0	0	0	8.8	1.6	0	0	0	0	0	0	0	0	0	0	0	1.5	7.2	0.8	0	0	0	0.8	2.3	6	2	0.2
1000519287	Azvudine group	5223.7	2440.1	2783.6	186	3.6	93.1	92.9	66.2	27	57.8	5.6	29.5	8.9	39.8	11	6.5	13.6	3.2	4.8	5.4	11.4	9.5	36.9	4	1.6	7.3	0.9	12.2	4.6	4.4	14.9	164.8	6.2	0.1
1000538607	Paxlovid group	1981.2	942.3	1038.9	875.4	44.2	360.1	515.3	151.4	208.8	127	33.4	354.9	40.4	31.2	39.9	39.9	70.3	51.1	42	45.5	31	69	27	22.4	11	69.2	39.2	64.1	110.1	72.3	18.3	303.4	438.1	115.6
1000587722	Paxlovid group	2680.2	1198.5	1481.8	547.6	20.4	405.1	142.5	153.6	251.5	22.9	29.2	90.4	49.6	37.7	26.8	39.4	26	80.4	79.5	65.6	7.2	3.5	12.2	3.8	25.4	25.6	4.7	10.9	20.2	29	62.2	200.9	212.1	72.3
1000587722	Paxlovid group	2680.2	1198.5	1481.8	547.6	20.4	405.1	142.5	153.6	251.5	22.9	29.2	90.4	49.6	37.7	26.8	39.4	26	80.4	79.5	65.6	7.2	3.5	12.2	3.8	25.4	25.6	4.7	10.9	20.2	29	62.2	200.9	212.1	72.3
1000625574	Azvudine group	3909.5	1910.8	1998.7	738.9	18.9	220.6	518.3	103.2	117.4	209	107.3	202	20	40.6	15.2	27.4	43.9	34.3	32.2	7	46.3	88.1	74.6	65.4	41.9	77.2	0.4	69.6	34.5	20.2	86.3	349.5	253.4	49.7
1000674022	Paxlovid group	5278	2200.5	3077.6	31.1	0.6	30.5	0.6	0	30.5	0	0	0.6	0	0	0	0	2.5	15.9	8.9	3.2	0	0	0	0	0	0	0	0	0	0.6	2.3	21.9	6.7	0.2
1000743528	Paxlovid group	2334.4	897.3	1437.1	252.8	10.8	42	210.8	27.7	14.4	134.3	60	16.4	4.3	3.7	7.9	11.7	0.5	8.8	2	3.1	67.9	48	18.3	48.4	11.6	0	10.4	6.1	0	0	34.9	125.4	72.7	19.7
1000783638	Paxlovid group	4251.5	1985.5	2266	49.8	1.2	38.3	11.4	25.9	12.5	3.1	4.6	3.7	9.6	6.4	6.6	3.3	0	8.1	4.4	0	0.4	2.7	0	0	4.6	0.2	0	1.8	0	1.7	5.9	32.7	10	1.2
1000833034	Paxlovid group	3343	1609.9	1733	230.8	6.9	118.7	112	10.3	108.5	18.5	4.3	89.2	2.5	2	0	5.8	0.5	39.6	30.2	38.2	0	18.5	0	0	4.3	19.3	5.7	32	2	30.1	16.4	141.8	62	10.6
1000843573	Paxlovid group	4031.2	1697.6	2333.6	33.8	0.8	8.1	25.6	4	4.2	0.8	4.2	20.6	0	2.7	0	1.3	0	0.5	3.7	0	0	0	0.8	3.8	0.4	9	0	0.6	2.8	8.3	4.3	25.3	4.1	0.1
1000845317	Azvudine group	4782.3	2271.8	2510.5	113.6	2.4	64.7	48.9	0.3	64.4	2.5	2.6	43.8	0	0.1	0	0.1	8.4	1.9	28.8	25.3	0.6	0.9	1	2.6	0	5.6	11.3	0.4	9.2	17.2	71.3	39.2	2.3	0.7
1000894449	Paxlovid group	3425.7	1496.9	1928.7	379.5	11.1	84.5	295	50.9	33.6	98.1	76.6	120.3	32.9	15.4	2.5	0.1	16	5.7	10.4	1.5	9.6	59.1	29.3	56.9	19.7	46.6	3.8	39.4	24.1	6.4	107.9	237.4	32.1	2.1
1000901328	Paxlovid group	2685	1217.3	1467.7	449.1	16.7	187.4	261.8	18	169.4	76.7	13.1	172	14.7	0	1.4	1.9	23.7	57.9	50.1	37.7	2.2	73.9	0.7	2.7	10.4	41.2	9.7	21.5	58.1	41.5	24.8	300.1	117.8	6.4
1000931768	Azvudine group	2828.7	1284.6	1544.2	7.8	0.3	4.9	2.8	4.5	0.4	0	2.8	0	0	0	0	4.5	0	0.4	0	0	0	0	0	1.8	1	0	0	0	0	0	3.1	4.3	0.3	0
1000938014	Paxlovid group	3202.1	1384.5	1817.7	807.7	25.2	173.5	634.1	134	39.5	188.3	28.7	417.1	33.2	86.3	4.7	9.7	8.4	4.2	7.4	19.5	35.6	88.5	64.3	22.2	6.5	137.3	7.5	90.4	115.9	66.1	222.4	475.2	83.7	26.3
1000963479	Paxlovid group	4094.4	2069.5	2024.9	658.4	16.1	97.8	560.7	44.9	52.8	229.3	49.9	281.5	0.8	19.1	21.1	4	0	17.6	16.3	18.9	43	82.5	103.8	14.8	35.1	96.5	7.2	46.1	37.6	94.2	37.9	502.3	116.2	2.1
1001017808	Azvudine group	4801	2148.8	2652.2	2228.6	46.4	1120.6	1108.1	660.7	459.8	484.8	254.3	369	212.4	225.5	131.6	91.3	108.5	122.4	118.4	110.4	169.2	72.7	242.9	146.2	108.1	70.2	22.4	116.8	105.9	53.7	574.1	1244.2	340	70.2
1001028235	Paxlovid group	3881.8	1802.6	2079.2	1067.1	27.5	506.6	560.4	261.8	244.8	292	100.2	168.2	66.1	94.1	71.3	30.3	48.9	4.1	51.1	140.8	71.1	127.5	93.4	41.6	58.6	76.8	2.9	33.4	44.8	10.4	370.8	540.5	122.9	32.9
1001069844	Azvudine group	4778.7	2221.2	2557.5	38.2	0.8	17.3	21	0	17.3	0	0	21	0	0	0	0	13.9	1.1	0.3	2	0	0	0	0	0	8.8	0	0.4	6.8	5	4.2	27.4	6.2	0.4
1001070557	Paxlovid group	3763	1540	2223	10.1	0.3	7.1	3	3.3	3.8	0	0	3	0.8	0	0	2.5	0.3	0.7	1.8	1	0	0	0	0	0	1.6	1.4	0	0	0	0.8	7.9	1.4	0
1001143616	Paxlovid group	3928.6	1581.4	2347.2	342.6	8.7	180	162.7	65.9	114	58.3	9.4	94.9	42.3	3.1	11	9.6	33.6	12.5	40.9	27	5	20.7	32.6	9.4	0	0.2	13.4	56.1	19.6	5.7	40.9	101.1	140	60.7
1001156030	Paxlovid group	3403.5	1610.1	1793.4	56.5	1.7	6.5	50.1	5	1.5	0.3	1.5	48.2	0	4.7	0.3	0	1.5	0	0	0	0	0.3	0	0	1.5	31.7	1.1	0.3	5	10.2	2	30.9	23.1	0.5
1001237355	Azvudine group	3272.2	1668.3	1603.9	173.6	5.3	104.3	69.3	0.4	103.9	1.5	1.9	65.9	0.3	0	0	0.1	0.2	9.6	41.8	52.4	0	1.5	0	0.5	1.4	21.4	1.6	0	0.9	42.1	54.5	92.9	19.9	6.3
1001257003	Azvudine group	2768.7	1220.6	1548.2	759.7	27.4	350.7	409	90.5	260.3	119.5	22	267.4	4.3	26.9	26.6	32.6	45.7	52.9	87.6	74.1	7.3	65.4	46.8	1.8	20.2	58.5	26.5	48.7	65.9	67.9	17.1	397.7	331.3	13.7

1001301785	Azvudine group	4231.8	1660.9	2570.9	845.6	20	300.1	545.5	164.4	135.7	256.8	12	276.7	78.1	31.8	33.5	21	38.2	38.8	39.7	18.9	90.8	142	24	4.6	7.3	49.5	6.2	61.5	80	79.6	81.8	497	236.4	30.4
1001360064	Paxlovid group	3764	1839.1	1924.9	112.2	3	26.8	85.4	0.6	26.3	1.1	4.5	79.7	0	0	0	0.6	12.7	0	2.5	11.1	0	1.1	0	3	1.5	13.5	0.3	1.1	42.9	22.1	16.9	90.4	4.6	0.3
1001360893	Paxlovid group	5007	2348.5	2658.5	0	0	0	0	0	0	0	0	0	0	0	0	0	0	0	0	0	0	0	0	0	0	0	0	0	0	0	0	0	0	0
1001383651	Azvudine group	2595.3	1168.5	1426.9	1182.7	45.6	390.5	792.2	190.4	200	318.7	106.4	367.2	92.4	27.7	26.5	43.9	38.3	43.2	63.4	55.1	141.4	138.3	39	55.2	51.1	60.7	47.9	79	105.2	74.4	55.6	500.8	504.8	121.5
1001418411	Azvudine group	2738.6	1147.6	1591	837	30.6	390.5	446.5	204.8	185.8	245.6	29.1	171.8	25.6	39	67.5	72.7	25.3	46.6	58.3	55.6	19.8	59	166.8	16.7	12.4	30.9	18.3	17.1	43.9	61.6	63	426.1	295.6	52.3
1001433297	Paxlovid group	1981.9	973.1	1008.7	1081.6	54.6	543.3	538.3	340.7	202.6	265	81.2	192.1	125.9	112.4	66.9	35.4	58.1	47.1	32	65.5	36.9	141.8	86.2	49.9	31.3	40.2	19.2	50.9	30.2	51.6	338.3	576.5	121	45.8
1001480398	Azvudine group	3147.3	1496.2	1651.1	31.3	1	10.2	21.1	6.7	3.5	12.6	0.5	8	0.8	0	0.3	5.6	2.2	0	0	1.3	2.2	8.6	1.8	0.5	0	2.9	0	2.1	2.4	0.6	3.1	25.3	2.9	0
1001481703	Paxlovid group	3676.7	1832.3	1844.4	65.4	1.8	26.7	38.8	8.6	18	21.8	3	14.1	0.2	7.9	0.5	0	13.7	0.1	0.1	4.1	1.9	12.1	7.7	2.5	0.4	3	0	4.8	6.2	0.1	13.5	35	14	2.9
1001493057	Azvudine group	3345.2	1506.7	1838.5	0	0	0	0	0	0	0	0	0	0	0	0	0	0	0	0	0	0	0	0	0	0	0	0	0	0	0	0	0	0	0
1001493129	Azvudine group	2818.6	1283.3	1535.3	167.8	6	0.4	167.4	0	0.4	147.8	15	4.6	0	0	0	0	0	0	0	0.4	19.8	27.7	100.3	13.5	1.5	2.4	0.2	1.9	0	0.2	86.1	71.3	7.7	2.7
1001497739	Paxlovid group	4835.5	2049.6	2785.9	10	0.2	9.2	0.8	0	9.2	0	0.8	0	0	0	0	0	0	4.9	0.3	4.1	0	0	0	0.8	0	0	0	0	0	0	0.9	5.8	3.2	0.2
1001497739	Paxlovid group	4835.5	2049.6	2785.9	10	0.2	9.2	0.8	0	9.2	0	0.8	0	0	0	0	0	0	4.9	0.3	4.1	0	0	0	0.8	0	0	0	0	0	0	0.9	5.8	3.2	0.2
1001498698	Paxlovid group	2672.4	1094.4	1578	1067.6	39.9	229.8	837.8	104.1	125.6	355.4	190.7	291.7	33.6	38.8	14.3	17.4	2.2	55.7	50.1	17.6	141.8	91	122.6	63.4	127.2	91.2	23.6	33.9	91.6	51.3	39.6	450	509.5	68.4
1001498850	Azvudine group	3418.6	1680.5	1738.1	7	0.2	0	7	0	0	0	0	7	0	0	0	0	0	0	0	0	0	0	0	0	0	1.8	0	0	0	5.1	2.7	4.2	0.1	0
1001499027	Paxlovid group	2021.6	872.8	1148.8	336.6	16.7	260.3	76.4	69.7	190.5	8.5	6.6	61.3	18.6	5.4	18.7	27.1	31.9	48.4	67.5	42.7	0.1	7.2	1.2	2.3	4.3	7.6	6.1	1.1	27	19.3	13.4	192.7	115.6	14.9
1001499070	Azvudine group	7889.2	3867.9	4021.3	206.2	2.6	25.4	180.8	1.7	23.7	66.8	3.5	110.4	0	0.4	1.3	0.1	3.8	0.6	6.5	12.8	16.8	47.8	2.2	3.5	0	30.1	9.4	14.5	18.1	38.4	116.4	61.7	18.9	9.2
1001499192	Azvudine group	3957.5	1747.3	2210.2	137.9	3.5	47.9	90	14.6	33.3	15.6	1.2	73.3	5.3	2.7	2.6	4.1	3.5	1.1	14.5	14.2	1.5	10.1	4	0.2	0.9	14.7	0.1	3.9	27.3	27.2	58.4	73.1	5.8	0.7
1001499299	Azvudine group	4665.7	2370.7	2295	23.4	0.5	0.2	23.2	0.1	0.1	22.7	0.2	0.3	0	0.1	0	0	0	0	0.1	0	0.3	22.3	0	0	0.2	0.2	0	0	0.1	0.1	5.6	12.2	4	1.5
1001499300	Azvudine group	4151.9	1855.9	2296.1	56.2	1.4	30.3	25.8	10.6	19.8	14.9	0.8	10.2	5.5	0.2	0	4.9	2.6	15.5	0.3	1.3	0.3	13.6	0.9	0.6	0.2	2.8	0.2	0.3	3.6	3.3	4.4	33.4	16.4	1.9
1001499316	Paxlovid group	2522.8	1096	1426.8	219.5	8.7	3.9	215.6	2.2	1.7	68	32.4	115.2	0.2	0	0.3	1.7	0.1	0.1	0.9	0.6	10.7	40.9	16.4	13.7	18.7	49	1.2	6.2	24.7	34.2	21.5	102.2	74	21.8
1001499325	Paxlovid group	5949.7	2646.9	3302.7	0	0	0	0	0	0	0	0	0	0	0	0	0	0	0	0	0	0	0	0	0	0	0	0	0	0	0	0	0	0	0
1001499353	Azvudine group	1987.6	853.2	1134.5	48.4	2.4	8.8	39.6	3.6	5.3	18.9	0	20.7	0.2	0	0	3.4	0	2	1.7	1.6	4.7	10.7	3.5	0	0	5	0	0.7	4.3	10.6	2.6	26	16.5	3.3
1001499356	Paxlovid group	3173.8	1231.2	1942.7	1277.7	40.3	295.8	981.9	14.2	281.6	80.8	102.2	798.9	0	0	0.8	13.4	15.3	71.9	95	99.4	0.2	71	9.6	47.1	55.2	128.3	84.4	153.5	201.6	231.1	117.6	626.2	489.4	44.5
1001499359	Azvudine group	2281.3	930.1	1351.2	97.9	4.3	55.6	42.3	0	55.6	6.3	4.9	31.1	0	0	0	0	6.6	6.2	16.7	26.1	0	6.3	0	2.6	2.3	5.6	1.8	0.6	2.8	20.3	7.5	57.4	30.5	2.5
1001499366	Azvudine group	4441.2	1924	2517.2	69.1	1.6	28.8	40.3	0.2	28.6	2.4	3.8	34.1	0	0	0	0.2	12.9	5.5	3.6	6.6	0	2.4	0	3.8	0	6.2	0	11.3	14.3	2.3	11.9	51.6	5.5	0.1
1001499550	Paxlovid group	2621.4	1154	1467.4	53.8	2.1	0.7	53.1	0.2	0.5	6.8	4	42.3	0.1	0	0.1	0	0	0	0	0.5	0.1	4.9	1.8	1.4	2.6	18	0.1	10.2	3.6	10.5	3.3	33.6	15.3	1.6
1001499554	Paxlovid group	2975.4	1018.2	1957.2	269.8	9.1	93.1	176.7	44.5	48.6	103.6	8.5	64.6	38.1	0	0	6.4	6.9	4.6	13.5	23.7	13.9	67.9	21.8	3.9	4.6	55.4	1.9	0.7	2.6	4	71	104.8	65.1	28.8
1001499606	Paxlovid group	4561.4	2271.6	2289.8	74.2	1.6	40.7	33.5	1.2	39.4	2	0	31.6	0	0	1.2	0	0	10.1	18.7	10.7	0	2	0	0	0	7.1	0.8	2.8	10.8	10.1	4.1	65.1	5	0
1001499626	Azvudine group	4149.7	1813.4	2336.3	308.6	7.4	84.4	224.2	19.4	65	50.6	7.9	165.7	7	6.6	5	0.8	9.1	33.8	13.5	8.6	8.1	15.5	27	7.5	0.4	17.6	5.1	32.3	49.4	61.3	99.7	139.7	51.3	17.9
1001499887	Azvudine group	5706.5	2651	3055.5	81.6	1.4	30.1	51.5	29.8	0.3	44.9	6	0.5	23.9	5.9	0	0	0	0.3	0	0	2.6	23	19.3	6	0	0.1	0.4	0	0	0	7.3	39.6	29.5	5.3
1001499895	Azvudine group	3038	2008.1	1029.9	238.4	7.8	203.7	34.7	73.4	130.3	0	3.1	31.6	12.4	23.5	10.4	27.1	41.9	20.4	26.7	41.3	0	0	0	1.6	1.6	16.8	0.3	1.7	12.2	0.6	50	94	63.7	30.7
1001499898	Azvudine group	2867.9	1274.7	1593.2	27	0.9	2.1	24.9	0.2	1.9	8.4	5.8	10.7	0.1	0	0	0.1	0	1.4	0.1	0.3	0	7.8	0.6	0.5	5.3	5.8	2.5	0.1	0.1	2.2	2.3	14	8.1	2.6
1001499919	Azvudine group	2956.3	1400	1556.3	8.5	0.3	2.3	6.2	0.3	2	0	0	6.2	0.3	0	0	0	0	0	1	1	0	0	0	0	0	3.4	0	0	0.6	2.1	0.2	7.4	1	0
1001499924	Azvudine group	3579.2	1752	1827.2	190.7	5.3	82.4	108.4	19	63.3	14.1	15	79.3	7.3	5.3	0.7	5.8	0	37.4	17.2	8.7	3.1	0.7	10.3	14.4	0.5	19.2	5.8	7.3	24.3	22.7	15.9	147	26.9	0.9
1001500188	Paxlovid group	3725	1702.1	2022.9	5.1	0.1	2.9	2.3	0.1	2.8	0	0.3	2	0	0	0	0.1	0.7	0	1.1	0.9	0	0	0	0	0.3	1.2	0.6	0	0	0.2	1.4	3.5	0.3	0
1001500204	Azvudine group	3675.8	1699	1976.8	0	0	0	0	0	0	0	0	0	0	0	0	0	0	0	0	0	0	0	0	0	0	0	0	0	0	0	0	0	0	0
1001500205	Azvudine group	4139.2	1952.5	2186.7	171.7	4.1	83.5	88.3	0.6	82.9	1.1	5.4	81.8	0.6	0	0	0	12	27.9	14.5	28.5	0	1.1	0	0	5.4	6.5	2.1	16.7	22.1	34.3	5.3	149.1	17.2	0.1
1001500296	Azvudine group	3989.7	1719.1	2270.6	5.8	0.1	5.8	0	3.3	2.4	0	0	0	0	0	0	3.3	0	2.4	0	0	0	0	0	0	0	0	0	0	0	0	0.4	4.3	1.1	0
1001500434	Paxlovid group	5104.2	2351.2	2752.9	0	0	0	0	0	0	0	0	0	0	0	0	0	0	0	0	0	0	0	0	0	0	0	0	0	0	0	0	0	0	0

1001500672	Azvudine group	3387.3	1465.9	1921.4	3.7	0.1	1.3	2.3	0	1.3	0.9	0.1	1.3	0	0	0	0	0	0	0.1	1.3	0	0.9	0	0	0.1	0.3	0	0.2	0	0.7	1.5	1.9	0.2	0
1001500751	Paxlovid group	5443.7	2324.4	3119.3	0	0	0	0	0	0	0	0	0	0	0	0	0	0	0	0	0	0	0	0	0	0	0	0	0	0	0	0	0	0	0
1001500755	Azvudine group	4288.7	1819.1	2469.6	0	0	0	0	0	0	0	0	0	0	0	0	0	0	0	0	0	0	0	0	0	0	0	0	0	0	0	0	0	0	0
1001500756	Azvudine group	5006.8	2678.1	2328.7	74.5	1.5	1.6	72.9	0.1	1.5	1.3	0.7	70.9	0.1	0	0	0	0	0	0	1.5	0.7	0.1	0.5	0.7	0	5.9	0.7	19.1	17.2	28.1	26.5	36	9.9	2
1001500885	Paxlovid group	4195.1	1967.1	2228	510.9	12.2	100.9	410	90.1	10.7	280.3	6.4	123.3	16.7	65.6	7.8	0	8.5	0	0.1	2.1	101.3	164.1	14.9	6.2	0.2	52.1	1.6	15.7	37.5	16.5	196.3	237.6	63.6	13.4
1001501102	Paxlovid group	3014.4	1190.8	1823.6	172.8	5.7	39.1	133.7	0.4	38.6	13.8	54.3	65.6	0	0	0.1	0.4	1.1	10.9	16.6	10.1	4.3	5.5	4	20.5	33.9	11.5	6.3	10.6	12.6	24.8	35.6	74.7	42.6	19.9
1001501119	Azvudine group	3879.2	1874	2005.2	0	0	0	0	0	0	0	0	0	0	0	0	0	0	0	0	0	0	0	0	0	0	0	0	0	0	0	0	0	0	0
1001501160	Paxlovid group	2425.5	1106.7	1318.8	754.5	31.1	340	414.4	122.1	217.9	110.9	79	224.5	37.9	27.3	20.2	36.7	46.8	51.5	58.5	61	17.2	57.7	36.1	38.1	40.9	50.3	17.7	37.4	69.1	50	70.5	418.1	222	43.9
1001501335	Azvudine group	2963.4	1093.1	1870.3	36.6	1.2	19.3	17.3	3.6	15.8	4	10	3.3	2.8	0	0	0.7	8.8	1.2	0	5.7	0.2	2.2	1.6	6.4	3.6	1.6	0.4	1	0.3	0	3.6	19.3	11.6	2.1
1001501339	Paxlovid group	2264.5	1065.1	1199.4	60	2.6	5.9	54.1	3.5	2.3	0.9	2.7	50.5	0.1	0.1	0.4	3	0	0.3	1.5	0.6	0	0.9	0	1.9	0.8	5.3	11.8	1.6	4.8	27.1	8.5	33.7	13.9	3.9
1001501384	Azvudine group	2662.9	1103.8	1559.1	122.3	4.6	91	31.3	0.8	90.2	4.3	15.1	12	0.6	0	0.1	0.1	0.4	4.6	25	60.2	0.2	3.5	0.7	12.6	2.5	1.3	6	1.3	2.5	1	21.7	75	19.8	5.9
1001501600	Azvudine group	3851.2	1852.4	1998.8	0	0	0	0	0	0	0	0	0	0	0	0	0	0	0	0	0	0	0	0	0	0	0	0	0	0	0	0	0	0	0
1001501603	Paxlovid group	5237.1	2004.1	3233	188.4	3.6	102.2	86.3	9.1	93.1	19.4	2.6	64.2	0	0.5	0	8.6	2.5	7.5	43.1	40	0.1	0	19.3	0	2.6	0.1	17.9	0.1	1.3	44.8	30.5	97	50.6	10.3
1001501667	Paxlovid group	3039.6	1440.8	1598.8	13.7	0.5	5.7	8.1	0.7	4.9	0.4	0.6	7	0	0	0.1	0.6	3.2	0	0.1	1.6	0	0.2	0.2	0.4	0.2	3.5	0	2.1	0.2	1.2	1	7.6	4.4	0.7
1001501731	Paxlovid group	1885.2	887.2	998	145.1	7.7	29.8	115.2	17.9	11.9	3	26.9	85.3	0.2	3.4	7.7	6.7	2.8	4	1	4.1	0.2	1.3	1.6	14.5	12.5	2.7	9.2	11.9	25.4	36	15.9	81.5	38.3	9.3
1001501797	Azvudine group	4154.3	1824.8	2329.5	538.6	13	201.3	337.3	5.9	195.4	71.7	30.1	235.5	1.4	0.7	2.1	1.8	2.1	25.4	107.9	60.1	5.8	44.4	21.4	7.8	22.3	65.8	28.6	25.4	57.4	58.4	187.2	261.2	67.7	22.6
1001501826	Azvudine group	4078.9	1978.2	2100.7	0	0	0	0	0	0	0	0	0	0	0	0	0	0	0	0	0	0	0	0	0	0	0	0	0	0	0	0	0	0	0
1001501918	Azvudine group	4826	2273.2	2552.8	0	0	0	0	0	0	0	0	0	0	0	0	0	0	0	0	0	0	0	0	0	0	0	0	0	0	0	0	0	0	0
1001501978	Azvudine group	3071.7	1291.8	1779.9	125.6	4.1	101.2	24.4	8.4	92.8	2.9	0.6	20.8	4.5	0.2	1.3	2.4	45.2	1.3	24.5	21.8	0	2.9	0	0	0.6	0.5	0.7	10.8	5.2	3.7	3	50.8	57.1	14.7
1001501989	Paxlovid group	3030.9	1321.7	1709.2	483.2	15.9	291.9	191.3	106.3	185.6	78.6	10.1	102.6	70.3	3	12.6	20.4	66.5	10	73.6	35.4	0.4	77	1.3	0.1	10	43.8	0.4	0.8	35.7	21.8	28.7	188.9	200.6	65
1001502063	Paxlovid group	2563.7	1179.3	1384.4	27.5	1.1	0.6	26.8	0	0.6	1	14.9	10.9	0	0	0	0	0	0	0.6	0	0	0	1	14.9	0	1.6	0.7	0.1	2.5	6	5.1	19.5	2.7	0.2
1001502224	Paxlovid group	3631.3	1608.1	2023.2	88.1	2.4	32.6	55.5	13.4	19.2	2.6	6.7	46.2	1.4	6	5.2	0.8	0.8	3.6	9.6	5.2	1.7	0.4	0.5	3.9	2.8	7.7	4.5	7.7	12.1	14.2	7.8	70.4	9.7	0.3
1001502311	Paxlovid group	5364.6	2439.9	2924.7	42	0.8	10.8	31.2	0.7	10.1	0	0.2	31.1	0.6	0.1	0	0	0	0	6.2	4	0	0	0	0.2	0	0.5	0.8	7.3	9.4	13.1	4.3	35.7	2	0
1001502315	Azvudine group	4180.1	1949.6	2230.5	0	0	0	0	0	0	0	0	0	0	0	0	0	0	0	0	0	0	0	0	0	0	0	0	0	0	0	0	0	0	0
1001502338	Paxlovid group	6333.9	3133.6	3200.2	213.7	3.4	55.8	157.9	24.2	31.6	114.9	1.3	41.7	24	0	0.2	0	7.7	2	4.4	17.5	22.7	92.2	0	0	1.3	6.2	0.4	8.7	16.7	9.8	36.9	136.4	34.1	6.3
1001502341	Paxlovid group	4572.3	2096.6	2475.7	0	0	0	0	0	0	0	0	0	0	0	0	0	0	0	0	0	0	0	0	0	0	0	0	0	0	0	0	0	0	0
1001502382	Paxlovid group	3101.2	1260.8	1840.4	43.1	1.4	35	8.1	0	35	0	0	8.1	0	0	0	0	8.5	0	3.3	23.2	0	0	0	0	0	1.1	0	0	0	6.9	1	39.4	2.7	0
1001502848	Paxlovid group	1869.6	871.7	998	208.7	11.2	15.6	193.1	0.8	14.8	114.5	71.3	7.3	0.8	0	0	0	5.2	0	0	9.5	11.4	0	103.1	32.3	39	0	7	0.3	0	0	19.4	153.3	33.2	2.8
1001502864	Paxlovid group	3043.7	1404.9	1638.9	0	0	0	0	0	0	0	0	0	0	0	0	0	0	0	0	0	0	0	0	0	0	0	0	0	0	0	0	0	0	0
1001502867	Paxlovid group	3273.8	1501.4	1772.4	7.1	0.2	5.6	1.6	0.6	5	1.6	0	0	0	0.6	0	0	0	1.3	3.4	0.3	0	0	1.6	0	0	0	0	0	0	0	0.6	5.1	1.5	0.1
1001503182	Paxlovid group	4062.5	1825.1	2237.4	229.4	5.6	157.8	71.7	49.6	108.1	6.3	0	65.4	29.8	3.5	11.4	5	21.1	3.4	35.1	48.5	0	4.3	1.9	0	0	58.1	0	0	2.7	4.6	26.4	165.2	37	0.9
1001503388	Paxlovid group	5295.3	2577.7	2717.7	15.6	0.3	8.1	7.5	0	8.1	0	0	7.5	0	0	0	0	0	0.5	4.2	3.5	0	0	0	0	0	0	0	2.5	2.3	2.7	0.6	14.1	0.9	0
1001503603	Azvudine group	2719.1	1204.5	1514.6	456	16.8	190.3	265.7	39.8	150.6	70	21.9	173.7	8.1	0	3.9	27.7	31.4	55.1	39.2	24.8	0.6	68	1.4	20.6	1.3	43.2	5.2	35.7	54.5	35.2	44.3	289.3	116.3	6.1
1001503671	Paxlovid group	2817.6	1169.2	1648.4	186.1	6.6	54.7	131.4	0.8	53.8	4.4	0	127	0.8	0	0	0	3.9	4.5	35.8	9.7	0	4.4	0	0	0	57.8	0	0.9	49.1	19.3	3.7	103.3	66.5	12.5
1001503716	Azvudine group	3102.7	1483.4	1619.3	0	0	0	0	0	0	0	0	0	0	0	0	0	0	0	0	0	0	0	0	0	0	0	0	0	0	0	0	0	0	0
1001504262	Paxlovid group	3405.4	1454.7	1950.7	6.5	0.2	4.7	1.8	0	4.7	0.6	0	1.2	0	0	0	0	0	2.4	2.3	0	0	0.6	0	0	0	0	0	0.9	0.3	0	0.2	3.7	2.4	0.2
1001504450	Paxlovid group	3586.2	1923.9	1662.3	89.2	2.5	7	82.3	1.7	5.2	3.7	0	78.5	0	0	1.4	0.4	0	5.2	0	0	3.1	0.6	0	0	0	25.4	0.2	1.8	19.6	31.6	6.5	29.8	41.9	11.1
1001504454	Azvudine group	5075.6	2381.2	2694.4	0	0	0	0	0	0	0	0	0	0	0	0	0	0	0	0	0	0	0	0	0	0	0	0	0	0	0	0	0	0	0
1001505002	Paxlovid group	2376.2	118.8	2257.4	223.5	9.4	0	223.5	0	0	33.2	34.1	156.2	0	0	0	0	0	0	0	0	4.8	10.2	18.2	8.3	25.9	5.7	19.7	25.9	43.4	61.3	15.1	173.1	34.7	0.6

1001505028	Paxlovid group	4360.2	1981.7	2378.5	0	0	0	0	0	0	0	0	0	0	0	0	0	0	0	0	0	0	0	0	0	0	0	0	0	0	0	0	0	0	0
1001505303	Paxlovid group	4647.9	2213.1	2434.8	0	0	0	0	0	0	0	0	0	0	0	0	0	0	0	0	0	0	0	0	0	0	0	0	0	0	0	0	0	0	0
1001506272	Azvudine group	3017.5	1365.1	1652.4	751.8	24.9	315.1	436.7	23.4	291.8	57.8	20.5	358.3	8.3	7.7	5	2.4	65.9	47.4	80.2	98.2	8.2	45.1	4.5	11	9.6	100.9	20.6	40.1	94.7	102	53.6	465.7	214.5	18
1001506357	Paxlovid group	4145.9	1881	2264.9	13.3	0.3	0.7	12.6	0	0.7	0	12	0.7	0	0	0	0	0.7	0	0	0	0	0	0	4.5	7.4	0	0.7	0	0	0	4.8	7.8	0.7	0
1001506402	Paxlovid group	2524.5	1025.3	1499.3	84.8	3.4	16.9	67.9	1.7	15.2	0	4.6	63.3	0	0	1.2	0.5	0.5	1.8	9.3	3.6	0	0	0	0	4.6	12	2.5	0.1	7	41.7	6.5	68.2	9.9	0.2
1001506482	Paxlovid group	6845.2	3203.2	3642.1	75.2	1.1	42.5	32.7	1.9	40.7	1.2	0	31.6	0	0	1.4	0.5	0	22.9	13.7	4.1	0	0	1.2	0	0	0	9	6.8	3	12.8	11.8	60.6	2.7	0.1
1001506497	Paxlovid group	5861.9	2700.2	3161.7	172.9	2.9	91.3	81.6	7	84.2	0.6	8	73	0	0	4.6	2.5	0	37	17.6	29.6	0	0.6	0	0	8	11.6	3.8	14	18.7	25	22.1	133.7	16.8	0.4
1001506610	Azvudine group	3952.8	1766.3	2186.5	320.2	8.1	190.8	129.4	65	125.8	50.5	3.5	75.4	27.6	4.9	12.3	20.2	24.4	24.4	25	51.9	3.6	36	10.9	1.7	1.8	35.1	4.3	8.3	4.1	23.6	48.2	232.2	38.9	0.8
1001506704	Paxlovid group	4792.2	2137.7	2654.5	0	0	0	0	0	0	0	0	0	0	0	0	0	0	0	0	0	0	0	0	0	0	0	0	0	0	0	0	0	0	0
1001506752	Paxlovid group	3546.6	1496.4	2050.2	651	18.4	166.4	484.6	71.5	94.9	60.5	95.2	329	8.4	25.3	16.5	21.3	8.8	24.4	40.6	21	5	14.4	41.1	30.7	64.5	41	14.8	56.2	108.8	108.3	87.3	471	89.6	3.1
1001506878	Azvudine group	2929.6	1291.2	1638.4	405	13.8	198	207	11.7	186.2	30.1	42.2	134.7	2.8	0	3.5	5.4	40.5	39	58.2	48.5	1	10	19.1	23.5	18.7	14.7	13.2	18.6	38.8	49.4	34	266.7	97.4	6.8
1001506921	Paxlovid group	4190.1	1792.4	2397.7	81.3	1.9	1.3	80	0.3	1	0.1	76.3	3.6	0	0	0	0.3	0	0.1	0.6	0.3	0	0	0.1	17.1	59.2	1.2	1.5	0.1	0	0.7	31.5	39.8	8.5	1.5
1001507231	Paxlovid group	3058.3	1511.6	1546.7	241.7	7.9	1.5	240.2	0.2	1.3	20.9	13.9	205.4	0	0.1	0	0.1	0	0.1	0.1	1.1	0	17.8	3.1	5.5	8.4	34.6	9.1	37	80.8	43.9	34.5	113	77.5	16.9
1001507695	Paxlovid group	4015.9	2001	2014.8	606.5	15.1	97.5	509	0.6	96.9	93.7	120.9	294.4	0.1	0	0	0.5	23.2	0.1	6.6	67	0.9	83.3	9.6	56.7	64.3	63.5	0.4	56.3	89.4	84.8	48.3	356.6	177	24.6
1001507700	Azvudine group	4532.5	2104.6	2427.9	880.4	19.4	237.5	642.9	23.2	214.3	268.6	38.9	335.4	10.3	1.6	0.4	11	52	65	51.3	45.9	55.8	169.5	43.3	15.6	23.2	123.3	9.4	40.4	92.9	69.4	90.4	590	190.5	9.6
1001507762	Paxlovid group	4193.8	1970.6	2223.2	315.5	7.5	172.8	142.7	70.8	102	17.1	6	119.7	22	14	17	17.9	7.1	2.9	42.7	49.2	11.5	2.1	3.5	0.2	5.8	49.4	1.6	21.7	21.7	25.2	19.9	218.5	75.7	1.5
1001507770	Paxlovid group	4878.6	2133.3	2745.3	1573.7	32.3	606.1	967.6	234	372.2	370.5	74.6	522.4	77.8	26.4	62.5	67.3	109.9	114.4	95.2	52.6	56.9	192	121.7	41.3	33.3	133	43.9	157.5	115.8	72.3	496.3	791.3	233.5	52.6
1001507852	Paxlovid group	3800.9	1941.3	1859.6	526.9	13.9	25.4	501.6	0	25.4	312.6	32.7	156.3	0	0	0	0	2.6	0	0.7	22.1	87.4	158.4	66.8	32.5	0.2	49.8	2.1	21.1	49.8	33.5	90.4	267.5	154.4	14.6
1001508045	Azvudine group	3242.5	1426.6	1815.9	617.1	19	201.9	415.2	71.9	130	106.8	66.9	241.5	16.9	9.9	34.1	11	37.2	30.8	27.4	34.6	7.5	71.3	28.1	46.3	20.5	80.6	7.3	68.9	58.6	26.1	38	498	78.7	2.3
1001508196	Azvudine group	4168	1784	2384	457.8	11	324.4	133.4	76	248.4	27.4	25.8	80.1	4.4	26.9	16.1	28.5	12	51.8	83.9	100.7	10.4	8.1	8.9	12	13.9	25	0.4	0	6.6	48.1	30.8	380.4	45.6	0.8
1001508315	Paxlovid group	4983.3	2272.9	2710.3	898	18	256	642	210.4	45.6	279.5	171.7	190.8	103.7	87.1	9.2	10.4	12.8	9.6	4.9	18.4	35.3	127.7	116.4	90.8	80.8	56.4	7	36.2	37.7	53.5	253.9	629	14.5	0.6
1001508345	Paxlovid group	2666.7	1045.8	1620.9	1016.2	38.1	299.7	716.5	223.3	76.4	340.2	100.3	276	108.8	28.1	55.3	31.2	22.7	37	13.4	3.2	86.6	139.7	113.9	91.7	8.6	71.4	9.8	86.3	48.7	59.8	83.4	684.6	241	7.2
1001508362	Paxlovid group	3773.9	1982.5	1791.4	1236.2	32.8	488.2	748	225.8	262.4	254.2	160.8	333.1	56.9	43.3	58.5	67	50.9	64.4	66	81.1	54.4	117.4	82.4	105.6	55.2	79.1	11.9	72.8	84.1	85.2	42.1	766.5	417.5	10.1
1001508422	Paxlovid group	3301.8	1001.7	2300.1	509.6	15.4	137.1	372.5	29.3	107.9	71.3	26.1	275.1	12.3	1	3.9	12.1	3.6	21.4	15.1	67.8	6.6	53.1	11.6	24	2.1	63.5	5.5	93.3	63.4	49.4	117.5	243.8	127	21.4
1001508428	Paxlovid group	5712.3	2830.1	2882.2	8	0.1	5.3	2.8	1.5	3.7	0	2.2	0.6	0	0	0	1.5	0	0	0	3.7	0	0	0	1.2	1	0	0	0.2	0.2	0.2	3	4.2	0.8	0.1
1001508558	Azvudine group	2387.9	626.2	1761.7	421.6	17.7	207.5	214.2	12.7	194.7	5.3	19.8	189	1.6	0	0.5	10.6	52.5	45.3	55	41.9	0	4.8	0.5	19.7	0.1	61.7	15.7	23.9	50.1	37.7	29.6	275.5	112.5	4
1001508562	Paxlovid group	3365.1	1532.8	1832.3	1443.9	42.9	702.6	741.3	403.5	299.1	401	81.7	258.6	102.4	154.6	83.5	63	59.9	102.1	77.9	59.2	60.2	161.9	179	53.9	27.8	87.5	10.3	93	51.9	15.9	455.4	727.7	199.5	61.3
1001508703	Paxlovid group	3172.5	1467.8	1704.7	1239.3	39.1	543.8	695.4	145.6	398.3	189.2	48.3	457.9	4.9	64.8	26.7	49.1	45.2	83.6	147.8	121.7	49.1	58.6	81.5	11.5	36.8	84.4	46.7	163	96	67.8	72.3	831.4	324	11.5
1001508736	Paxlovid group	5307.5	2487.7	2819.8	1257.2	23.7	577.2	680	136.1	441	177.6	61.8	440.6	22.1	30.7	31.6	51.7	64.2	152.5	98	126.3	6.2	98.9	72.4	21.4	40.4	96.1	15.1	71.5	134.9	123	292.7	921.4	41.7	1.4
1001508782	Paxlovid group	4896.3	2140.1	2756.2	695.5	14.2	294.2	401.3	242.9	51.3	260.7	34.5	106.1	138.3	79.9	22.8	1.9	23.6	0.1	8.1	19.6	116.3	107	37.4	17.5	17	69.8	0.6	4.3	28.7	2.6	72.6	443.7	171	8.2
1001508807	Paxlovid group	4129.3	1832.7	2296.6	1551.6	37.6	780.6	770.9	426.8	353.8	296.9	45	429	167.6	59.9	119.8	79.5	85.9	86.8	84.2	96.9	76.1	174.2	46.7	22.5	22.5	107.3	47.8	58.5	123.2	92.2	432.2	932.1	148.6	38.7
1001508812	Paxlovid group	3618.6	1645.5	1973.1	489.3	13.5	288.3	201	198.8	89.5	43.4	12.7	144.9	97.5	101.3	0	0	30.3	4.7	24.9	29.6	16.1	13.1	14.1	3.9	8.9	18.8	2.9	36.8	46.2	40.1	50.1	398.6	40	0.6
1001508812	Paxlovid group	3618.6	1645.5	1973.1	489.3	13.5	288.3	201	198.8	89.5	43.4	12.7	144.9	97.5	101.3	0	0	30.3	4.7	24.9	29.6	16.1	13.1	14.1	3.9	8.9	18.8	2.9	36.8	46.2	40.1	50.1	398.6	40	0.6
1001508913	Paxlovid group	3815.8	1661.7	2154.1	214.1	5.6	154.8	59.3	88.8	65.9	11.8	13	34.5	3.1	42.5	31.3	11.9	0.4	21	34.9	9.6	2.4	3.3	6.2	9.7	3.4	10.1	2.4	1.3	8.4	12.4	51.1	128.4	30.7	3.9
1001508964	Paxlovid group	2725.7	1317.7	1408	745.2	27.3	341.1	404	172.5	168.6	102.8	69.2	232	56.5	23.5	46.8	45.8	31	54.9	47.3	35.4	6.5	59	37.3	35.9	33.4	51.9	13.3	49.9	56.5	60.2	177.4	387.9	156.5	23.3
1001508980	Paxlovid group	6827.8	3411.3	3416.5	39.3	0.6	9.2	30.1	1	8.2	13.9	4	12.2	0.3	0.3	0.1	0.2	2.2	0	5.1	0.9	8.6	0.5	4.8	2.6	1.4	0.6	2.5	0.4	2.8	5.9	21.4	14.8	2.6	0.5
1001509079	Paxlovid group	2529	1354.5	1174.6	966.2	38.2	579.9	386.3	258.7	321.1	213	29	144.3	55.4	83.6	55.8	63.9	59.3	108.1	88.7	65	56.9	83.3	72.8	21.9	7.2	52.5	15.1	23.6	21.3	31.7	31.3	507.8	397.1	30
1001509081	Paxlovid group	4449.8	2242.7	2207.1	798.6	17.9	156.4	642.2	23.6	132.8	117.7	174.6	349.8	0	6	2.7	14.8	43.9	34.1	33.3	21.5	6.2	55.6	55.9	121.6	53	69	15	68.3	105.5	92	215.1	488.9	78.1	16.5
1001509084	Paxlovid group	3126.2	1287.9	1838.4	1028.5	32.9	402.9	625.6	210.4	192.5	175.6	65.4	384.7	122.3	29.2	36.8	22.1	41.1	23.9	80.3	47.2	49	113.9	12.7	43.6	21.7	103.3	20.8	110.6	96.9	53.1	128	553.7	293.4	53.4

1001509110	Paxlovid group	2842.8	1382.9	1459.9	1676.6	59	643.6	1033	331.2	312.4	605.4	80.3	347.3	119.7	88.2	70	53.3	39.5	107.4	101.7	63.8	145.2	212.2	248	22.6	57.6	70.7	39.9	58.5	91.1	87.2	110.1	1008.6	485.3	72.5
1001509124	Paxlovid group	4598.4	2102.8	2495.6	908.9	19.8	376.1	532.9	110.2	265.9	156.1	41.3	335.5	51.3	7.8	21.2	29.9	90.6	35.2	107.7	32.4	4.2	93.6	58.3	16.8	24.5	130	13.2	6.6	112.2	73.5	132.4	417.3	285.1	74.2
1001509140	Azvudine group	3139.1	1199	1940.1	154.5	4.9	129.1	25.3	26.3	102.8	3.2	9.9	12.3	0.8	1.4	12.9	11.1	6.7	24.5	38.4	33.3	0	3.1	0.1	1.2	8.7	1.1	0.5	3.7	1.6	5.4	31.3	93	25.3	4.9
1001509151	Paxlovid group	4074.5	1933.5	2141.1	130.4	3.2	38.3	92	11.2	27.1	42.4	15.9	33.8	3.1	1.8	3.8	2.5	3.3	1.1	11	11.7	6.3	7.2	28.8	14	1.9	6.9	0.4	0.7	21.6	4.2	19	84.7	23.4	3.2
1001509181	Paxlovid group	2230.5	1010.9	1219.6	242.2	10.9	94.6	147.6	11.9	82.7	8.3	21.4	117.9	0.5	4.1	0.5	6.8	1.3	28.1	11.1	42.2	1	0.6	6.7	6.3	15	24.1	9.7	16.8	33.8	33.4	15.9	154	66.7	5.6
1001509183	Paxlovid group	3560	1255.2	2304.8	1083.5	30.4	395.7	687.8	128.3	267.4	134.3	31.1	522.4	14.1	48.1	53.1	13	35.1	97.5	73.9	60.9	29.4	50.4	54.4	7.8	23.4	79	46.2	122.8	145.8	128.7	127.8	624.4	252.7	78.6
1001509188	Paxlovid group	3675.3	1780.2	1895.1	1677.3	45.6	710.7	966.6	324.9	385.7	349.2	88.3	529.1	89.7	84.1	94.9	56.2	72.2	108	104.6	100.9	125.9	112.7	110.6	51.5	36.7	133	23	156.1	91.1	126	170.2	823.7	603	80.4
1001509218	Paxlovid group	5100.6	2623.1	2477.5	907.6	17.8	255.4	652.2	33.7	221.6	255.7	65.8	330.8	6.1	11.5	8.3	7.9	75.9	32.4	49.8	63.6	27.1	176.8	51.7	53.9	11.9	80.9	12.6	81.8	93.6	61.9	78.9	789.4	38.5	0.8
1001509233	Paxlovid group	2412.2	985.7	1426.5	179.6	7.4	13.4	166.1	7.4	6	59.6	25	81.6	5.6	1.5	0	0.3	2	2.8	1.2	0	13.8	20.9	24.9	18.4	6.6	45.3	0.6	6.8	24.2	4.6	17.1	125.9	35.1	1.5
1001509251	Paxlovid group	3445.5	1556	1889.5	984.5	28.6	374.7	609.9	193	181.7	205.1	116.7	288.1	51.2	38.8	46.6	56.3	60.7	46	20.2	54.7	34	122.2	48.9	80.7	36	84.5	8.7	55.3	50.8	88.8	370.1	488.3	110.1	16
1001509259	Paxlovid group	2599.8	1096.1	1503.7	1737.1	66.8	590.2	1146.9	411.3	178.9	520.7	266.8	359.4	86	32.7	112	180.6	17.4	56.1	67.1	38.3	182.1	145.8	192.8	139.6	127.2	100.9	38	90.9	75.2	54.5	70.7	835.8	691.1	139.4
1001509272	Paxlovid group	2654.2	1259.9	1394.3	755.1	28.4	293.3	461.8	125.4	167.9	148.6	33.3	279.9	27.9	13.4	42.3	41.8	16	90.7	51.9	9.3	38	72	38.6	20.7	12.6	63.8	12.5	71.1	102.3	30.1	26	267.8	384.1	77.1
1001509335	Paxlovid group	3047.9	1513.6	1534.4	1332.9	43.7	532.2	800.8	256.3	275.9	326.7	101.7	372.3	61.6	61.2	68.2	65.3	56	70	79.9	70	64.9	146.4	115.5	58.2	43.5	81.2	30.5	121	72.1	67.5	238	787.9	279.7	27.3
1001509354	Paxlovid group	4607.6	2246	2361.6	497.7	10.8	213.7	284	16.6	197.1	1.9	108.8	173.3	0	0.3	0.3	16.1	7.6	109.3	60.3	19.9	0	0	1.9	42.3	66.5	1.8	15.7	14.6	70.4	70.8	61.8	363.5	71.2	1.2
1001509385	Paxlovid group	2664	1285.4	1378.6	306.6	11.5	76	230.6	22	54	79.9	4.1	146.6	12.3	3.1	6.4	0.3	1.6	13.6	18.6	20.1	29.5	44.9	5.5	2.6	1.5	42.8	1.2	24.7	55.2	22.8	22.1	229.1	53.2	2.2
1001509393	Paxlovid group	4489.9	2143.3	2346.6	1006.2	22.4	475	531.2	147.4	327.6	192.6	170.9	167.7	54.1	10.6	35.5	47.2	30.9	79.9	60.8	156	29.3	103.7	59.6	118.1	52.9	46	3.2	19.8	67.5	31.1	249.4	727.9	27.5	1.5
1001509401	Paxlovid group	4230.8	1779.6	2451.2	1588.8	37.6	662.5	926.3	243	419.5	222.2	209.9	494.2	70.6	46.1	50.1	76.2	51.8	148.4	120.7	98.7	36.1	95.3	90.8	115.7	94.2	115.9	38.7	114.5	123.3	101.8	118.1	752.3	617	101.4
1001509462	Paxlovid group	3965.6	1623.3	2342.3	1512.4	38.1	757.8	754.5	376.5	381.3	288.1	70.3	396.1	140.1	21.1	91.7	123.6	83.2	58.1	107.5	132.5	28.4	168.8	90.9	45.8	24.5	126.6	27	69.9	94.9	77.8	56.2	619	741.7	95.5
1001509481	Paxlovid group	2799.3	1321.1	1478.2	859.9	30.7	324.1	535.9	153	171.1	212	48.9	274.9	44.4	59.1	31.9	17.6	36.1	33.6	45.2	56.2	48.6	95.3	68.1	31.7	17.2	72.3	3.2	48.7	86.3	64.4	49.7	529.9	271.8	8.5
1001509512	Paxlovid group	4587.8	2139.7	2448.1	886.8	19.3	342.6	544.2	37.5	305.1	172.4	38.5	333.3	16.7	0	1.8	19	95.3	104	68.6	37.3	20.6	143.1	8.8	19.9	18.6	120.4	6.3	61.7	69.8	75	100.8	644.1	138.7	3.2
1001509526	Paxlovid group	3644.1	1586.9	2057.2	1873.4	51.4	738.8	1134.6	397.2	341.6	471.4	186.2	477	98.4	134.7	104.7	59.3	46.6	91.7	127.8	75.5	136.7	124	210.8	146.8	39.4	105.9	35.2	104.5	105.2	126.4	260.9	1034.1	537.5	40.9
1001509575	Paxlovid group	2532.2	1132	1400.2	1811.9	71.6	749.4	1062.5	364.8	384.6	422.2	173.1	467.2	118.2	71.1	100.6	74.9	36.5	119.2	146.4	82.4	123.6	111.9	186.7	90.8	82.4	121.9	52.5	100.9	109.4	82.6	45.9	1031.3	706.3	28.4
1001509596	Paxlovid group	2171.3	878.1	1293.2	889.4	41	409.7	479.8	240.6	169.1	150.9	86.2	242.6	76.7	58.2	69.8	35.9	63.9	44.7	33.1	27.4	11.3	95.9	43.7	39.7	46.4	69	20.8	67.4	36.7	48.8	99.1	540.7	229.5	20.1
1001509630	Paxlovid group	2627.5	1486.8	1140.7	1327.3	50.5	385.9	941.4	139.5	246.5	339.2	149.7	452.5	18.1	9.5	33.6	78.3	2.1	61.2	86.3	96.9	180.3	60.9	98	99.5	50.2	109	46.3	71.3	148.2	77.8	30.1	444.1	692.4	160.7
1001509635	Paxlovid group	5492.9	2589.9	2902.9	2105.4	38.3	785.9	1319.4	272.8	513.1	527.2	122.6	669.7	131.5	3.1	40.9	97.4	64.5	239.7	88.8	120.1	129.3	233.1	164.8	93.5	29	170.8	50.9	125.5	145.3	177.1	589.7	1387.3	102.4	25.9
1001509636	Paxlovid group	2464.7	1105	1359.8	1097.4	44.5	568.7	528.7	206.9	361.8	296.4	46.6	185.7	88	42.8	39.7	36.4	48.5	77.7	133.8	101.8	158.6	128.5	9.3	34.7	11.9	53.1	13.7	34.8	59.1	25	123.1	541.2	370.2	62.9
1001509637	Azvudine group	2699	1272.3	1426.7	586.7	21.7	244.2	342.5	32.1	212.1	85.2	25.9	231.5	7.9	20.7	0.3	3.2	38.3	46.9	35.3	91.6	1.4	28.3	55.5	19.9	5.9	75.2	15.6	33.5	64.1	42.9	27.1	311.3	214.1	34.2
1001509666	Paxlovid group	2281.5	1031.4	1250.1	1157.6	50.7	626.1	531.4	306.6	319.5	289.3	49.4	192.7	135.1	49.3	60	62.2	58.3	100.5	87.4	73.3	70.2	141.1	78	22.9	26.5	67.9	15.4	47.8	27.5	34.1	72.7	818.9	251.3	14.7
1001509673	Paxlovid group	3707.6	1650.1	2057.5	474.1	12.8	194.1	280.1	63.6	130.5	58.9	46.2	174.9	15.5	18.8	21.5	7.7	5.5	31.1	51.9	41.9	5.3	30.9	22.7	31.4	14.8	48.4	5.5	45.9	44.1	31	46.8	332	93.4	2
1001509716	Paxlovid group	2921.8	1280.2	1641.6	627.4	21.5	115.7	511.7	97.2	18.5	195.7	26.8	289.2	2.4	0.2	27.3	67.1	5.8	11.2	0	1.5	79.6	115.5	0.6	18.3	8.5	108.2	13.5	22.2	103.5	41.9	34.7	457.3	130.6	4.8
1001509764	Paxlovid group	3515.6	1593	1922.5	2259	64.3	814.7	1444.3	366.7	447.9	495.5	223.7	725.1	93	34.1	157.5	82.2	71.3	129.4	133.1	114.1	222	116.8	156.6	78.5	145.3	218.8	33	179.2	160.2	133.9	115	1560.8	564.7	18.5
1001509795	Paxlovid group	3804.9	1667.1	2137.8	0	0	0	0	0	0	0	0	0	0	0	0	0	0	0	0	0	0	0	0	0	0	0	0	0	0	0	0	0	0	0
1001509810	Paxlovid group	3032.1	1271.2	1760.9	818.3	27	450.5	367.8	230.4	220.2	79.9	39.3	248.6	89.3	20.8	52.7	67.6	49.7	72	61.9	36.5	4.7	75.2	0	17.9	21.4	81	14.7	59.3	52.1	41.4	129.1	444.7	210.7	33.8
1001509863	Paxlovid group	4654.1	2086.6	2567.5	287.5	6.2	123.1	164.3	9.9	113.2	2.8	0.4	161.1	0	0.2	0.8	9	0	5.8	62.3	45.1	1.8	0.9	0	0.4	0	0.1	3.7	27.4	59	71	10.9	181.8	90.1	4.6
1001509957	Azvudine group	2295.1	787.1	1508	151.5	6.6	71.6	80	34.1	37.5	1.7	4.6	73.7	22.3	0	2.5	9.3	3.5	15.4	18.4	0.1	1	0.6	0	0.7	3.9	13.4	10	8.5	18	23.8	4	91.5	54.3	1.7
1001509972	Paxlovid group	3011.7	1658.7	1353	618.5	20.5	106.7	511.7	51.7	55	126	32.5	353.3	11.4	1.7	3.4	35.2	3	20.3	10.8	20.9	8.3	101.8	15.9	22.8	9.7	79	47.7	48.8	85.5	92.3	3.5	470.9	142.9	1.1
1001510021	Paxlovid group	3407.1	1267.2	2139.9	59.1	1.7	30.7	28.4	1.5	29.2	9.7	13.4	5.3	1.5	0	0	0	9.8	0.9	4.8	13.7	0	0	9.7	11	2.4	0.7	0.4	0.1	0.1	4.1	5.2	35.5	16.7	1.7
1001510043	Paxlovid group	3580.7	1472.2	2108.5	342.5	9.6	336.5	6	235.5	101	5.2	0.2	0.6	110.8	21.5	60.6	42.5	11.2	33.6	42.3	13.8	2	0	3.2	0.2	0	0.2	0	0	0.2	0.2	39.6	162.7	114.3	25.9
1001510077	Azvudine group	3845.6	1931.3	1914.4	766.4	19.9	229.4	537	66.5	162.9	175.3	68.5	293.2	20.4	1.5	24.8	19.7	28.5	15.4	46.4	72.6	28	107	40.3	59	9.5	102.1	8.5	50.1	68.1	64.4	49.8	467.7	236.5	12.3

1001510114	Azvudine group	3848.8	1786	2062.8	691.3	18	123.9	567.4	97.9	26.1	276.3	105.3	185.8	29.4	23.5	35.6	9.3	8.8	1.8	8.9	6.6	45.8	134.7	95.7	65.4	40	38	2.7	64.3	50.4	30.4	95.8	535.6	59	0.9
1001510119	Paxlovid group	2815.3	1217.9	1597.4	862.2	30.6	403.2	459	149.2	253.9	97.2	97.2	264.6	41.8	35.4	39.7	32.3	23.5	66.5	88	76	3.9	54.2	39.2	83.5	13.7	71.2	14.9	31.8	84.3	62.4	112	435.8	266.2	48.3
1001510136	Paxlovid group	2683.2	1249.1	1434.1	256.6	9.6	128.7	128	24.6	104.1	22.8	1.9	103.3	19.5	0	0.3	4.9	27.5	12.7	31.6	32.3	3.1	4.3	15.3	1.2	0.7	24.4	0.4	14.9	24.7	38.9	60.1	157	36.2	3.3
1001510205	Azvudine group	3215.1	1447.3	1767.8	0	0	0	0	0	0	0	0	0	0	0	0	0	0	0	0	0	0	0	0	0	0	0	0	0	0	0	0	0	0	0
1001510313	Paxlovid group	2204.9	946.1	1258.8	951.5	43.2	489.8	461.7	161.6	328.2	97.9	81.9	281.9	51.3	29.3	36.8	44.2	67.1	71.2	106.8	83.1	25.8	8	64	53.4	28.5	82.2	16	75.9	70.7	37.2	9	340.7	525.2	76.5
1001510320	Azvudine group	3186.2	1816.3	1369.9	688.1	21.6	246.7	441.4	137.3	109.4	194.2	9.3	238	56.6	63.9	9.4	7.4	36.7	17.8	28.6	26.3	79.2	60.3	54.6	1.3	8	67.6	5.3	25	76.3	63.8	42	530.2	112.1	3.7
1001510366	Paxlovid group	4662.9	2389.8	2273	26.7	0.6	0.6	26	0.3	0.3	1.4	4.4	20.2	0	0	0.3	0	0.2	0	0.2	0	0.1	0.5	0.9	3.6	0.8	2	0	7.2	9.7	1.2	5.8	18.6	2	0.2
1001510375	Paxlovid group	3415.5	1470.7	1944.8	4.9	0.1	1.5	3.4	0	1.5	0	0	3.4	0	0	0	0	0.3	0	0	1.2	0	0	0	0	0	1	0	0	0	2.3	0	4.4	0.4	0
1001510399	Paxlovid group	3079.7	1625.6	1454.1	1030.7	33.5	347.8	683	22.7	325	249.8	63.6	369.6	3.8	1.2	11.1	6.6	59.2	75.2	113.3	77.3	89.9	76.3	83.5	47.7	15.9	85.6	65.3	53.3	94.8	70.6	215	484.5	245.6	85.7
1001510543	Paxlovid group	2564.9	1223	1341.9	298.6	11.6	41	257.7	28	12.9	17.8	13.5	226.4	3.1	1	11.3	12.7	11.3	1.7	0	0	0	2.1	15.7	8.6	4.9	63.9	5.9	38.2	59.7	58.7	10.6	228.9	57.8	1.3
1001510579	Paxlovid group	3274.1	1334	1940.1	1359.4	41.5	569.1	790.3	219	350.1	168.4	153.2	468.6	69.7	21.2	60	68.1	67.9	82.4	104.6	95.1	47.4	68.2	52.8	49.4	103.9	123.3	38.1	98.9	117.3	91.1	41.9	878.9	429.8	8.8
1001510638	Paxlovid group	2267.8	1039.8	1228.1	253.8	11.2	102.3	151.5	24.1	78.2	11.4	7.4	132.7	0.9	5.9	12.6	4.6	2.5	18.5	42.3	14.8	0	2.4	9	1.9	5.5	19.7	7.9	21.9	32	51.2	8	187.9	57.1	0.9
1001510649	Paxlovid group	2824	1052.1	1771.9	826.2	29.3	490.9	335.3	239.4	251.5	64.2	2.9	268.1	80.8	25.4	60	73.2	26.5	74.5	94.2	56.3	7.5	56.6	0.2	1.5	1.5	69.6	7.9	35.9	75.3	79.3	19.7	379.2	389.5	37.9
1001510652	Paxlovid group	4555.8	2080.2	2475.6	802.6	17.6	452.6	350	123.8	328.8	32.2	21.2	296.6	13.5	30.5	28.8	51	21.5	63.1	107.9	136.3	0.7	19.7	11.8	7.4	13.8	72.5	7.8	29.3	80.2	106.8	22.7	519.9	256	4
1001510678	Paxlovid group	2443.6	1027.8	1415.8	919	37.6	418.9	500.1	158	260.9	92.7	70.8	336.6	46.6	32.4	39.5	39.5	32.6	88.5	85.9	53.9	3.5	69.2	20	34.6	36.2	60.5	48.8	72.6	111.1	43.7	116.9	462.9	277.4	61.9
1001510853	Paxlovid group	2069.5	799.5	1270.1	836.7	40.4	179.7	657	111	68.6	275.1	67.9	314	22.6	53.4	19.3	15.7	13.9	38.9	7.4	8.3	77.4	80.1	117.6	46.8	21.2	95.6	32.2	54	65.4	66.8	29.2	443.1	344.2	20.2
1001510893	Paxlovid group	2335.5	1105.9	1229.6	182.9	7.8	21.8	161.1	8.3	13.5	70.8	8.8	81.6	0	4	4	0.3	7	3.4	2	1	14.4	11.9	44.5	4.2	4.6	11.9	1.7	37.9	23.8	6.2	9.5	139.3	33.3	0.8
1001510908	Paxlovid group	5197	2298.6	2898.4	581.1	11.2	286.1	295	20.2	265.9	0.2	11.6	283.3	0	13.1	2.8	4.3	12	42.1	115.9	95.9	0	0	0.2	5.1	6.5	4.3	1.8	22.3	112.8	142	64.4	454.9	60.4	1.3
1001510924	Paxlovid group	3417.5	1589.2	1828.4	1850.6	54.2	699.9	1150.7	308.6	391.3	421.5	237.7	491.5	37.9	60.6	109.9	100.2	61.8	104.1	130.7	94.8	103.8	124.3	193.4	141.1	96.6	130.8	35.3	135.1	92.5	97.8	61.3	1284	492.2	13.1
1001510963	Paxlovid group	1756.4	729.5	1026.9	430.9	24.5	192	238.8	43.2	148.8	43.2	81.8	113.9	28.5	2	1.5	11.3	56.2	23.1	24.2	45.3	5.6	23.2	14.4	29.7	52.1	31.6	16.5	14.2	32	19.6	41.3	181.4	146.5	61.7
1001510967	Paxlovid group	3782.9	1793.7	1989.2	1545.9	40.9	602.6	943.4	349.6	253	450.7	53.1	439.6	95.1	131.6	51.2	71.6	39	21.7	37.7	154.5	98	196.3	156.4	35.6	17.4	173	11	114.6	96.1	45	58.2	731.4	687.5	68.8
1001510981	Azvudine group	2501.9	1277	1225	302.5	12.1	148.6	153.9	75.8	72.8	68.9	55.6	29.4	3.9	18.7	38.4	14.7	21.4	13.6	18.8	19	2.1	32.4	34.4	53.8	1.8	17.4	0	2.8	0.1	9.1	27.3	229.2	45.1	0.9
1001511035	Paxlovid group	3425.6	1744.8	1680.7	1565.7	45.7	757.3	808.4	367.8	389.5	320	156	332.4	200.7	112.6	30.4	24.1	94	74	125.7	95.7	96.8	93.5	129.7	134.4	21.6	108.2	12.8	22.6	106.5	82.2	193.9	1079.5	268.8	23.4
1001511036	Azvudine group	1724.1	754.2	969.9	385.1	22.3	122.6	262.6	57.4	65.2	161.5	51.5	49.6	35.8	18	0.9	2.7	21.8	8.5	20.8	14.2	45.9	60.8	54.8	16.3	35.3	23.5	0.5	17.4	5.9	2.2	20.3	235.1	119.9	9.9
1001511064	Paxlovid group	6640.4	3266.9	3373.4	0	0	0	0	0	0	0	0	0	0	0	0	0	0	0	0	0	0	0	0	0	0	0	0	0	0	0	0	0	0	0
1001511172	Azvudine group	3251.8	1546.4	1705.4	146.7	4.5	52.7	93.9	7.1	45.6	35.6	0.8	57.6	0.9	0.1	0.8	5.3	0.1	12	17.2	16.4	1.8	29.7	4.1	0.7	0.1	5.8	7	29.1	10.2	5.5	22.1	60	42.9	21.7
1001511225	Paxlovid group	2333.5	1141.3	1192.2	242.7	10.4	153.5	89.2	3.4	150	22.1	2.8	64.3	2.5	0	0.7	0.3	22.9	29	48.4	49.7	0	22.1	0	0.4	2.4	19.6	4.9	3.8	20.6	15.4	5.3	135.9	95.3	6.2
1001511272	Paxlovid group	2697.8	1169	1528.8	282.5	10.5	92.3	190.2	32.6	59.7	37.1	3.3	149.8	0.3	21.3	10.8	0.1	3.5	17.9	20.3	18	0.9	5.6	30.7	3.3	0	12.3	10.6	19.7	51.8	55.4	6.7	179.9	93.8	2.2
1001511317	Paxlovid group	3870.7	1638	2232.6	267.3	6.9	86	181.2	14.9	71.1	19.7	11.2	150.3	1.9	1.9	2.1	9.1	1.2	18.2	22	29.7	6.6	7.4	5.7	2.8	8.4	13.1	12.3	31.3	46.8	46.9	119.2	123.2	18.8	6.1
1001511385	Paxlovid group	2253.5	1124.8	1128.7	335.4	14.9	84.4	250.9	13.9	70.5	47.5	12.9	190.5	2.9	0.6	1.7	8.7	2.2	28.3	19.5	20.5	9.6	30.1	7.8	9.9	3	35.3	15.6	53	34.1	52.6	19.2	244.3	69.3	2.6
1001511388	Paxlovid group	3287.7	1473.8	1814	35.6	1.1	1.8	33.8	0.8	1.1	0	0	33.8	0	0.4	0.3	0	0	0	0	1.1	0	0	0	0	0	19.7	0.8	0	6.4	6.8	2	27.4	6.2	0.1
1001511416	Paxlovid group	1871.2	797.7	1073.5	843.3	45.1	268.2	575.2	147.2	120.9	246.1	81.1	248	24.2	49.2	50.4	23.4	29.4	54.9	30.2	6.4	60.1	33.9	152.1	35.4	45.8	82.8	20.2	54.4	72.7	17.9	17	417.8	377.7	30.9
1001511446	Paxlovid group	3744.6	1739.7	2005	1854.1	49.5	892.4	961.7	512.2	380.2	482.2	101.7	377.8	183.1	122.9	112.9	93.2	57	85	114.1	124.2	116.8	159.2	206.2	62.6	39.1	94.7	8.8	105.3	95.4	73.5	571.3	1089.2	152.4	41.2
1001511520	Paxlovid group	3953	1713.5	2239.5	318.4	8.1	136.3	182.1	3.5	132.8	10.4	29.9	141.8	0	1.2	0	2.3	37.2	2	65	28.6	0.5	2.5	7.4	18.8	11.1	0.8	10.5	21.9	45.7	63	15.7	255.1	46.7	0.9
1001511533	Paxlovid group	5015.8	2195.1	2820.7	595.7	11.9	239.3	356.4	62.3	177	142.1	11.2	203.1	21.6	5	21.5	14.1	31	27	48.6	70.5	10.4	119.6	12	9.2	1.9	89.4	4.2	22.9	63.3	23.3	172	306.9	93.1	23.6
1001511546	Azvudine group	3363.9	1370.6	1993.4	394.2	11.7	255.9	138.2	8.8	247.1	15.9	0.6	121.7	5.7	0.9	1.9	0.4	59.8	74.9	59.1	53.3	1.2	14.5	0.2	0.6	0	45.6	1.2	1	22.6	51.3	40.5	185.1	131.8	36.7
1001511550	Paxlovid group	3005.6	1232	1773.7	115.1	3.8	21.3	93.8	13	8.3	17.7	28	48.1	0	0.9	6.5	5.6	0	4.8	0	3.5	1.6	7.2	8.9	17.5	10.5	23.5	9.3	10.2	4	1.1	10.1	71.2	28.8	4.9
1001511571	Paxlovid group	2791.2	1232.8	1558.4	578.2	20.7	253.7	324.5	110.3	143.4	61.9	58.5	204.1	19.5	31.7	36.1	23	25.1	46.7	40.6	31	0	22.6	39.3	27.7	30.9	11	28.7	37.2	56.5	70.6	22.5	398.3	147.4	10
1001511599	Azvudine group	3213.9	1351.8	1862	1030.7	32.1	352.9	677.8	104	248.8	237.5	81.8	358.5	24.9	29	13.1	37	45.6	59.8	88.2	55.2	47.3	110	80.2	47.9	33.9	82.9	19.3	95.4	83.8	77.2	106.1	489.6	383	51.9

1001511622	Paxlovid group	3418.5	1497.9	1920.6	488.3	14.3	92.5	395.8	13.7	78.9	122.9	55.2	217.7	7.5	0	2.5	3.7	13.2	25.1	20.1	20.4	0.5	71.1	51.3	28.7	26.5	40.8	17	56.1	52	51.7	60.4	372.8	54.2	0.9
1001511627	Paxlovid group	2052.8	946.3	1106.4	789.3	38.5	214	575.4	200.1	13.9	269.4	2.6	303.3	58.7	52	73.2	16.2	8.8	3.1	1.3	0.7	42.7	73.1	153.6	2.6	0	12.7	144.8	106.9	29.8	9.1	126.6	461	181.3	20.5
1001511678	Azvudine group	2699.4	1291.1	1408.3	299	11.1	136.3	162.7	49.8	86.5	40.5	22.5	99.7	3.9	12.5	23.1	10.3	2	43	21.5	20	13.7	19.6	7.2	3	19.5	9.6	16.9	42.6	2.6	28	34.2	165	87.3	12.4
1001511757	Paxlovid group	2368.4	1199.9	1168.5	782.1	33	297.4	484.6	141.1	156.3	103.2	76.1	305.4	19	59.9	42.1	20.1	2.6	48.1	49.8	55.8	13.4	17.8	72	40.2	35.9	56.6	20.7	70.5	88.9	68.7	99.7	404.6	228.7	49.1
1001511842	Paxlovid group	3969.3	1703.3	2266	829.2	20.9	270.4	558.9	19.9	250.5	94.9	47.1	416.8	0.3	0.7	5.5	13.4	20.7	72.4	90.8	66.6	9	53	32.8	26.8	20.3	114.1	7.4	76.9	124.8	93.7	83.7	486.1	239.1	20.4
1001511860	Paxlovid group	2495.4	1186.8	1308.6	351.9	14.1	31.7	320.3	0	31.7	102.9	4.7	212.6	0	0	0	0	1.3	0	8.3	22.1	8.6	74.8	19.6	3.3	1.4	75.9	0.5	48.7	57.1	30.3	8.5	238.1	103.6	1.7
1001511878	Azvudine group	4653.7	2091.2	2562.5	298	6.4	63.8	234.3	1.4	62.3	60.4	0.1	173.8	0	0	0	1.4	14.5	13.1	12.3	22.5	0.7	58.4	1.3	0.1	0.1	67	4.4	6.3	65.7	30.3	70.9	158.1	53.4	15.7
1001511898	Azvudine group	1435.7	604.6	831.1	919.3	64	384.7	534.6	207.3	177.4	203.9	93.4	237.3	80	31.7	41.1	54.5	43.6	47.2	57.3	29.3	46.7	66.3	90.9	40	53.4	67.5	25.1	33.8	76.8	34.2	6.7	426	468.6	18
1001511921	Azvudine group	2777.4	1302.3	1475	965.2	34.8	374.9	590.3	203.8	171.1	273.4	61.7	255.3	97.4	55.7	35.9	14.8	26.7	49.2	58.1	37.1	49.4	148.8	75.2	45.6	16	52.3	9.7	70.5	55.3	67.4	121.7	513.6	268.7	61.2
1001511922	Paxlovid group	2751.2	1476.5	1274.6	304.1	11.1	75.8	228.2	27.6	48.2	75.2	11.7	141.4	0.5	6.5	9.5	11.1	0	15.5	25.3	7.4	12	48.6	14.6	5.2	6.5	33.6	5.7	16.3	57.3	28.4	55	218.6	30	0.5
1001511932	Paxlovid group	4253.2	1970.7	2282.4	183.9	4.3	82.7	101.3	39.2	43.4	7.2	10.1	83.9	21.8	7.6	2.1	7.8	3.9	9	20.9	9.7	0	7.1	0.1	10.1	0	14.2	0.8	12.6	35.5	20.9	7.6	139.2	35.8	1.3
1001511962	Azvudine group	2240.9	1286	954.8	257.4	11.5	39	218.4	15.1	23.8	48.3	1.6	168.5	8.1	3.2	1	2.8	0	0.2	8.4	15.3	19.9	16.2	12.2	0.6	1	15	26.5	37.8	51.7	37.6	42.9	128.6	62.4	23.4
1001511963	Azvudine group	2540.7	1146.3	1394.4	441.6	17.4	231.9	209.8	124.4	107.4	70.9	7.3	131.6	39	19.3	43	23.1	23.8	27.1	44.8	11.7	7.7	44.7	18.6	1.6	5.7	31.2	2.1	11.8	35.8	50.7	23.1	263.8	135.1	19.7
1001512009	Paxlovid group	2328.7	1027.4	1301.3	306.6	13.2	50.7	255.9	40.8	9.9	53.5	7.8	194.6	13.4	7.3	9.2	11	5.9	1.8	0.4	1.8	0.5	19.2	33.7	6.6	1.2	63.1	18.9	5.2	33.1	74.3	8.4	230.8	65.6	1.8
1001512075	Paxlovid group	2337.8	1050.1	1287.6	19.9	0.9	4.9	15.1	0.3	4.5	0.3	4.2	10.6	0.3	0	0	0	0.2	0.7	2.3	1.2	0.3	0	0	4.2	0	2.9	0	0.7	2.8	4.1	0.2	11.4	7.5	0.8
1001512170	Paxlovid group	3439.3	1785.9	1653.4	14.1	0.4	2.7	11.4	0.1	2.5	0.8	0.1	10.5	0	0	0	0.1	0.1	1.7	0.5	0.2	0	0.8	0	0	0.1	7.8	0	0.1	1.8	0.8	4.9	7.6	1.4	0.2
1001512297	Paxlovid group	2856.9	1270.9	1586	425.5	14.9	308.6	116.9	83	225.6	1.3	66.8	48.9	42.2	11.7	16.2	12.9	56.4	59.4	61.4	48.3	0	1.2	0	28.9	37.8	14.3	2.4	1.2	10.6	20.4	20.6	261.9	134.3	8.7
1001512298	Paxlovid group	3172.3	1217.7	1954.6	192.8	6.1	123	69.8	16.4	106.6	1.2	23.9	44.7	0.4	6.5	4.9	4.6	6.6	32.5	38.3	29.3	0	0	1.2	0.2	23.6	8.4	4.9	11.5	9	10.9	8.7	126.5	55.5	2.1
1001512329	Paxlovid group	3085.1	1216.3	1868.8	965.8	31.3	472.1	493.7	169.8	302.3	99	38.3	356.3	52.6	65.5	31.4	20.4	35.7	103.7	107.8	55	20.6	61.6	16.8	31.8	6.5	54.5	20.6	143.5	82.5	55.2	45.7	711.4	206.8	1.9
1001512437	Paxlovid group	2321.8	1134.5	1187.2	235.9	10.2	55.4	180.6	3.4	52	28.3	16.7	135.6	0	0	2.7	0.7	4.5	7	20.6	19.9	2.1	15.4	10.7	8.6	8.2	36.3	6.6	6.4	52	34.2	8.8	178.9	45.2	3
1001512445	Paxlovid group	2840.5	1370.4	1470.1	911.3	32.1	394	517.3	117.6	276.4	148	42.7	326.6	7.7	16.8	56.9	36.2	48.8	67.2	99.7	60.6	31.9	33.5	82.6	35.3	7.4	52.3	23.6	40.2	122.6	87.9	95.2	580.9	215	20.2
1001512477	Paxlovid group	6076.6	2779.5	3297.1	349.3	5.7	12.2	337.1	7.7	4.4	46.3	29	261.8	0.6	0	2.4	4.7	0.3	0.1	2.3	1.8	5.1	26.3	14.8	15.1	14	49	7.3	139.1	61.6	4.8	134.2	167.7	42.5	4.9
1001512579	Azvudine group	3872.5	2076.5	1796	889.7	23	167.8	721.9	83.3	84.5	203.3	169.2	349.5	14.6	6.8	28.1	33.8	10.7	19.4	28.8	25.6	68.9	94.1	40.3	144	25.1	128	17	59.3	81	64.2	47.9	571.7	260.9	9.2
1001512593	Azvudine group	3957.6	1777.6	2180.1	1749.9	44.2	528.6	1221.3	115	413.6	399.3	144.2	677.8	20.8	3	31.9	59.3	63.9	148	100	101.7	91.4	145.3	162.6	102.1	42.1	168.6	50.8	159.1	185.1	114.2	123.6	1052.7	529	44.5
1001512713	Azvudine group	3655.9	1947.7	1708.1	720.1	19.7	283.6	436.6	88.4	195.2	366.6	64.9	5.2	68.5	1.8	11.3	6.7	60.7	45.8	80.8	7.8	116.2	43.3	207.1	57.4	7.4	4.8	0	0.3	0	0	61.3	362.5	258.9	37.4
1001512788	Paxlovid group	6198.1	2711	3487.2	63.4	1	1.9	61.5	0.7	1.2	36.2	0	25.2	0.4	0	0.3	0	0	0.6	0.2	0.4	2.3	33.5	0.5	0	0	4.7	0	0.4	3.5	16.6	10.5	49.1	3.7	0.1
1001512810	Paxlovid group	3767.8	2111.5	1656.3	903.1	24	576	327.1	133.3	442.6	73.9	0.5	252.8	15	10.7	30.8	76.8	16.3	155.5	169.9	101	1.9	68.1	3.9	0.5	0	97.1	12.1	7.7	46.1	89.8	79	554.9	243.6	25.6
1001512817	Paxlovid group	3378.2	1518.4	1859.8	25.7	0.8	6.1	19.6	5.2	0.9	5.8	0.7	13.1	0.3	0.4	3	1.5	0	0.5	0.4	0.1	1	2.7	2.1	0.6	0.1	1.3	0	7.1	2.2	2.5	4.2	18	3.4	0.2
1001512970	Paxlovid group	3740.1	1642.4	2097.7	306.8	8.2	126.8	180	70.3	56.5	0	0.7	179.3	27.7	13.9	6.1	22.6	11.6	19.7	14.5	10.7	0	0	0	0	0.7	19	10.4	41.5	40.2	68.2	14.3	218.8	71.4	2.3
1001512976	Azvudine group	4738.5	2076.3	2662.1	0	0	0	0	0	0	0	0	0	0	0	0	0	0	0	0	0	0	0	0	0	0	0	0	0	0	0	0	0	0	0
1001513547	Azvudine group	3804.9	1667.1	2137.8	0	0	0	0	0	0	0	0	0	0	0	0	0	0	0	0	0	0	0	0	0	0	0	0	0	0	0	0	0	0	0
1001513565	Azvudine group	2104	827.6	1276.4	496.5	23.6	218.3	278.2	91.5	126.9	35.4	60.3	182.5	13.4	28.8	22	27.3	41.7	30.7	23.6	30.9	7.5	20.9	6.9	14.2	46.1	47.9	0.7	50	59.5	24.4	60.3	261.5	151.6	23.1
1001513682	Paxlovid group	3219.2	1519.3	1699.9	92.1	2.9	3.2	88.9	2.3	0.8	4.6	3.8	80.5	0.7	0.3	0.6	0.8	0	0.8	0	0	0.3	2.7	1.5	1.2	2.7	14.2	1.3	21.9	23.8	19.4	10.6	61.6	18.3	1.7
1001513769	Paxlovid group	2534.1	1133.1	1401	1574.5	62.1	614	960.5	343	271	391.9	94.9	473.7	109	64	94.2	75.7	63.2	61.9	64.1	81.9	111.7	145.2	135.1	35	59.8	96.5	46.7	101.3	125.7	103.6	25.6	916.5	619.5	12.9
1001513829	Azvudine group	2976.1	1389.1	1587	124.5	4.2	41.5	82.9	11.6	29.9	31.5	0.7	50.8	7.3	0	0	4.4	1	8.4	12.7	7.8	0.3	30.9	0.3	0	0.7	5	0	9.1	17.6	19	6.6	98.6	19.2	0.1
1001513912	Azvudine group	3053.9	1362.9	1691	437.3	14.3	125.2	312	59.7	65.5	123.6	18.5	170	31.2	2	10.1	16.4	12.9	20.3	24.2	8.2	6	69.3	48.3	14.5	4	52	0.3	43.8	62.2	11.6	36.6	262.8	123.1	14.8
1001513938	Paxlovid group	3300.4	1384.2	1916.2	408.5	12.4	169.9	238.7	11.5	158.3	56	10.4	172.3	2.5	0	6.2	2.8	0.2	0.5	34.5	123.2	12.2	7.7	36.1	6.8	3.6	16.4	3.1	65.1	63.8	23.9	113.2	239.6	48.5	7.2
1001513996	Azvudine group	4769.8	2238.3	2531.4	333.2	7	135	198.1	25	110	4	1.8	192.4	4.6	2.3	1.9	16.2	4.1	48.3	31.6	26	2.3	1.7	0	0.5	1.3	14.6	28.3	13.6	52	83.8	95	197.5	35.7	4.9
1001514127	Azvudine group	2847.8	1614	1233.8	524.2	18.4	142.1	382.1	47.9	94.2	152.9	71.1	158.1	0.5	0	16.2	31.1	4.3	15.8	38.2	35.9	11.4	81.4	60.1	51.2	20	33.9	12.6	62.7	28.1	20.7	171.3	187.4	107.1	58.5

1001514157	Azvudine group	2713.9	1071.2	1642.8	672	24.8	245.6	426.5	113.7	131.9	174.4	9.7	242.4	80	13.3	15.2	5.2	19.8	12.4	52.9	46.7	48.2	76.8	49.4	2.9	6.8	69.8	10.3	35.6	88.1	38.6	45.1	462.9	152.4	11.6
1001514167	Azvudine group	4320.9	2062.9	2258	415.7	9.6	133.9	281.8	0.6	133.3	2.4	119.9	159.4	0.6	0	0	0	39.8	0.6	24.8	68.1	1.7	0.4	0.3	36.7	83.3	38.8	4.1	45.6	54.7	16.2	46	119.8	176.5	73.3
1001514299	Paxlovid group	5822.5	2537.7	3284.8	101.3	1.7	81.2	20.1	0.5	80.7	0.1	0	20	0	0.1	0	0.4	0.5	2.8	26.6	50.8	0	0.1	0	0	0	0	6.2	0.2	10	3.5	20.7	54	21.4	5.2
1001514365	Azvudine group	3078.3	1385.3	1693.1	447.3	14.5	86	361.3	6.7	79.3	182.8	68.8	109.8	4.1	0	0.5	2.1	13.8	0.1	15.7	49.7	53.9	51.8	77.1	48.1	20.7	16.9	3.3	65.5	12.4	11.8	7.4	180.8	221.3	37.9
1001514509	Paxlovid group	2948.5	1128.5	1820	618	21	216.3	401.7	110.9	105.4	107.8	11	283	46.4	26.3	20.5	17.6	24.9	18.1	34.6	27.7	15.3	82.4	10.1	6.3	4.7	85.7	5.9	54.2	93.2	43.9	19.3	382.1	201	15.6
1001514559	Paxlovid group	2377	1101.2	1275.8	1526.4	64.2	708.9	817.5	323	385.9	327.7	70.9	418.9	94.5	38.9	54.7	134.9	54.4	76.6	98	156.8	104.4	134	89.3	43.3	27.6	76.2	34.4	105.3	122.7	80.3	49.6	377.3	871.5	228
1001515002	Paxlovid group	4540.9	2174.6	2366.3	0	0	0	0	0	0	0	0	0	0	0	0	0	0	0	0	0	0	0	0	0	0	0	0	0	0	0	0	0	0	0
1001515325	Azvudine group	3359.6	1555.8	1803.9	29.8	0.9	5.8	24	5.8	0	18.1	0	5.9	1.4	0	0.3	4.2	0	0	0	0	2.4	15.2	0.5	0	0	3.5	0	0	0	2.5	0.7	26	3.1	0.1
1001515623	Paxlovid group	3327.3	1477.3	1850	655.6	19.7	316.7	338.9	148.6	168	18.2	7	313.8	42.2	50.1	35.9	20.4	9.3	34.3	48.4	76	4.7	5.8	7.6	0.7	6.3	13.3	11.2	60.7	69.7	158.9	82.2	428.3	136.6	8.4
1001515709	Paxlovid group	1567.4	723.1	844.2	21.4	1.4	7.9	13.5	3.2	4.7	5.6	0	7.9	1.3	0	0.2	1.8	0	0	1.7	2.9	0	0.2	5.4	0	0	0	0.7	0	2.9	4.2	0.3	14.8	6.1	0.2
1001515832	Paxlovid group	5252.5	2766.4	2486.1	131.6	2.5	18.1	113.5	4.3	13.8	45.2	15.1	53.2	3.2	0	1.2	0	1.9	0.2	4.5	7.1	12	28.1	5.1	13.5	1.5	30.1	0.1	0.4	21.9	0.6	34.6	87.3	8.9	0.9
1001515881	Paxlovid group	2912.5	1181.6	1730.8	446.9	15.3	252.4	194.5	21.3	231.1	4.6	4.9	185	9.8	2.1	2.8	6.6	30	58.8	80.7	61.7	1.1	2.5	1.1	2.4	2.5	69	12.4	10.2	55.7	37.7	5.3	267.8	159.5	14.3
1001516174	Paxlovid group	3341.1	1538.8	1802.4	1005.1	30.1	412.4	592.7	257.7	154.7	222.1	109.2	261.3	166.5	61.8	25.6	3.8	54.3	26	34.9	39.5	83.2	123.2	15.8	57.5	51.8	69.6	13.2	58.7	80.6	39.3	71.1	605.2	300.5	28.3
1001516201	Paxlovid group	2311.8	1423.9	887.9	22	1	22	0	16.8	5.2	0	0	0	0.7	0	2	14.1	0.6	0	2.8	1.8	0	0	0	0	0	0	0	0	0	0	0.2	14.5	7.3	0.1
1001516202	Paxlovid group	3727.9	1831.9	1896	310.4	8.3	69.5	240.8	34.1	35.4	36.8	1.7	202.3	1.7	1.1	21.5	9.9	8.6	1.1	7.8	17.8	0.5	36.1	0.1	0.3	1.4	51.9	1.2	17.9	62.1	69.1	48.8	179.2	73.2	9.1
1001516283	Paxlovid group	4063.4	1787.2	2276.2	102.7	2.5	70.1	32.5	25.6	44.5	16	1.9	14.7	11.5	0	1.2	13	3.6	12	18.5	10.3	0	14.4	1.6	0	1.9	0.4	2.7	0.4	8.4	2.7	18.6	77.3	6.5	0.3
1001519865	Paxlovid group	2785.6	1446.9	1338.7	187.7	6.7	24.5	163.2	8.4	16.1	0	3.7	159.4	2.5	2	3.5	0.5	0.6	4.9	5.5	5.2	0	0	0	3.6	0.1	46	16.7	18	28.8	50.1	9.1	43.1	85	50.4
1001520158	Paxlovid group	3324.6	1839	1485.6	1753	52.7	1239.7	513.3	650.9	588.8	398.3	94.6	20.4	119.9	164.5	163.3	203.2	9.9	210.3	178.1	190.4	173.3	6.6	218.5	75.3	19.3	8.9	0.7	4.4	1.2	5.2	284.8	1140	247.6	80.6
1001520201	Azvudine group	5307.3	2464.6	2842.7	203.9	3.8	68.5	135.4	42.4	26.1	94.4	6.3	34.7	17.9	8.4	14.9	1.2	2.6	1.9	8.2	13.4	52.6	21.3	20.5	1.9	4.4	4.5	4	3.2	5	17.9	30.6	136.9	31.4	5
1001520209	Azvudine group	3417.1	1358.3	2058.8	1609.3	47.1	753.2	856.1	405.2	348	411	88.4	356.8	154.4	131.6	73.4	45.8	82.5	60.4	126.6	78.5	158.6	30.5	221.9	57.3	31.1	50.3	30	99.3	146.4	30.8	344.3	591.3	497.2	176.5
1001523061	Paxlovid group	5131.2	2336.6	2794.6	0	0	0	0	0	0	0	0	0	0	0	0	0	0	0	0	0	0	0	0	0	0	0	0	0	0	0	0	0	0	0
1001523301	Paxlovid group	3130.6	1277.1	1853.6	1613.9	51.6	609.9	1003.9	308.1	301.8	317.8	191.1	495	110.5	88.6	72.8	36.2	56.6	69.3	91.5	84.4	65.4	75.7	176.7	60.6	130.5	130.1	35.9	120.9	124.1	83.9	76.4	1286.9	239.1	11.5
1001531337	Paxlovid group	2934.1	1399.1	1535.1	14.6	0.5	2	12.6	0.9	1.1	1.8	2	8.8	0.5	0.4	0	0	0.3	0	0.5	0.3	0	0.4	1.4	0.2	1.9	2.3	0	0.7	3.8	1.9	1.7	12.6	0.4	0
1001533418	Paxlovid group	5825.9	2607.2	3218.7	205.4	3.5	95.3	110.1	80.6	14.7	107	2.5	0.6	24.7	25.5	14.2	16.1	13.2	1.4	0	0.1	28.5	34.2	44.3	2.5	0	0	0	0.3	0.3	0	33.6	104.6	55.3	12
