# Supplementary material for: Antiviral Effectiveness, Clinical Outcomes, and Artificial Intelligence Imaging Analysis for Hospitalized COVID‐19 Patients Receiving Antivirals
Source: Influenza Other Respir Viruses. 2024 Sep 16;18(9):e70006. doi: 10.1111/irv.70006 (PMC11405122; doi:10.1111/irv.70006)
Supplement: Supplementary file 2 — Data S2. Supporting Information. [file IRV-18-e70006-s002.pdf]

医保卡号	treatment	Volume.of.whole.lung.cm..	Volume.of.left.lung.cm..	Volume.of.right.lung.cm..	Infected.Volume.of.whole.lung.cm..	Infection.ratio.of.whole.lung...	Infected.Volume.of.left.lung.cm..	Infected.Volume.of.right.lung.cm..	Infected.Volume.of.left.upper.lobe.cm..	Infected.Volume.of.left.lower.lobe.cm..	Infected.Volume.of.right.upper.lobe.cm..	Infected.Volume.of.right.middle.lobe.cm..	Infected.Volume.of.right.lower.lobe.cm..	Infected.Volume.of.Left.Upper.Lobe.Apicoposterior.Segment.cm..	Infected.Volume.of.Left.Upper.Lobe.Anterior.Segment.cm..	Infected.Volume.of.Left.Upper.Lobe.Superior.lingular.Segment.cm..	Infected.Volume.of.Left.Upper.Lobe.Inferior.lingular.Segment.cm..	Infected.Volume.of.Left.Lower.Lobe.Superior.Segment.cm..	Infected.Volume.of.Left.Lower.Lobe.Anteromedial.basal.Segment.cm..	Infected.Volume.of.Left.Lower.Lobe.Lateral.basal.Segment.cm..	Infected.Volume.of.Left.Lower.Lobe.Posterior.basal.Segment.cm..	Infected.Volume.of.Right.Upper.Lobe.Apical.Segment.cm..	Infected.Volume.of.Right.Upper.Lobe.Posterior.Segment.cm..	Infected.Volume.of.Right.Upper.Lobe.Anterior.Segment.cm..	Infected.Volume.of.Right.Middle.Lobe.Lateral.Segment.cm..	Infected.Volume.of.Right.Middle.Lobe.Medial.Segment.cm..	Infected.Volume.of.Right.Lower.Lobe.Superior.Segment.cm..	Infected.Volume.of.Right.Lower.Lobe.Medial.basal.Segment.cm..	Infected.Volume.of.Right.Lower.Lobe.Anterior.basal.Segment.cm..	Infected.Volume.of.Right.Lower.Lobe.Lateral.basal.Segment.cm..	Infected.Volume.of.Right.Lower.Lobe.Posterior.basal.Segment.cm..	Infected.Volume.of.HU....750..cm..	Infected.Volume.of.HU..750..300..cm..	Infected.Volume.of.HU..300.49..cm..	Infected.Volume.of.HU.50...cm..
0000014232	Paxlovid group	3585.4	1501.7	2083.7	0	0	0	0	0	0	0	0	0	0	0	0	0	0	0	0	0	0	0	0	0	0	0	0	0	0	0	0	0	0	0
0000014986	Paxlovid group	4784.7	3141	1643.7	212.7	4.4	30.9	181.8	5.2	25.7	46.5	33.9	101.4	2.1	0.3	0.2	2.7	7.7	2.4	5.1	10.5	4.8	38.6	3.2	31	2.9	30.3	0.6	18.3	24.5	27.8	86	76.1	38.8	11.8
0000014986	Paxlovid group	4784.7	3141	1643.7	212.7	4.4	30.9	181.8	5.2	25.7	46.5	33.9	101.4	2.1	0.3	0.2	2.7	7.7	2.4	5.1	10.5	4.8	38.6	3.2	31	2.9	30.3	0.6	18.3	24.5	27.8	86	76.1	38.8	11.8
0000017375	Paxlovid group	3568.8	1707.4	1861.4	476	13.3	294.1	181.9	106.4	187.7	64.5	19.6	97.9	7.6	7.9	56.2	34.7	58.3	14.2	62.5	52.7	13.8	22.1	28.6	9.6	10	49.9	15.7	12.5	7.8	12	22.7	337.6	111.9	3.8
0000030715	Paxlovid group	4052.2	1763	2289.2	32.7	0.8	17.1	15.7	1.6	15.5	5.1	1.2	9.4	0	0	0	1.6	11.5	0	0.9	3.1	0	5.1	0	0	1.2	6.3	0	0	1	2.1	3.6	23.5	5.3	0.2
0000061646	Paxlovid group	3131.9	1432.8	1699.1	247	7.9	93.4	153.6	0.1	93.3	6.2	69.4	78	0	0	0	0.1	7.8	25.8	34.5	25.2	0	4.4	1.8	31.2	38.2	6.8	19.1	6	6.8	39.4	16.6	187	42.3	1.1
0000064567	Paxlovid group	2186.9	1007.1	1179.8	660.5	30.2	388.9	271.6	186.7	202.2	73.1	32.3	166.1	86.7	46.9	33.5	19.6	43.8	33.1	70.4	54.9	0	58.1	15	20.5	11.8	32.6	13.3	10.1	53.7	56.4	67.4	357.8	192.2	43.1
0000086612	Paxlovid group	2925.7	1306.9	1618.8	1871.9	64	855	1016.9	470.5	384.5	496.4	76.2	444.3	195.8	99.9	106	68.8	66.9	72.2	116.4	129	153.1	155.3	188.1	39	37.2	111.2	50.1	81.7	124.5	76.8	199.9	1017.7	549.5	104.8
0000108840	Paxlovid group	4560.3	2512.5	2047.8	394	8.6	186.3	207.6	51.3	135	25.1	23.8	158.7	6.1	10	24.8	10.5	66.1	23.2	11.6	34.2	1.7	10.3	13.2	18.7	5.2	15.3	0.1	37.5	65.4	40.4	45.8	270.2	70.8	7.1
0000121825	Paxlovid group	5581.2	2645.5	2935.7	1808.4	32.4	647.7	1160.7	245	402.7	492.1	109.5	559	123.9	1.1	41.1	79	71.5	178.1	81.9	71.1	126.8	228.1	137.2	94.2	15.3	129.2	27.8	129.4	136.3	136.3	244.8	1516.6	45.1	1.9
0000146688	Paxlovid group	4638.6	2171.5	2467.1	209.5	4.5	10.3	199.2	2.4	7.9	30.2	19.8	149.2	1.1	0	0	1.3	0	2.3	5.3	0.3	0.5	21.9	7.7	19.7	0	25.6	0	34.6	55.6	33.5	18.1	152.8	38.1	0.5
0000150781	Paxlovid group	3173.9	1387.2	1786.7	570	18	340.2	229.8	214.9	125.3	95.6	21	113.2	97.7	30.4	48.2	38.6	52.2	17.2	42.6	13.3	29.8	27.9	37.9	16.4	4.7	2.2	2.1	1.9	65.9	41.1	33.4	381.8	146.5	8.2
0000158011	Paxlovid group	2315.5	1041.5	1273.9	124.4	5.4	1.8	122.6	1.2	0.6	2.7	0.4	119.5	0.5	0	0	0.7	0.1	0	0.1	0.4	0	2.7	0	0	0.4	32.3	6.5	0	35.3	45.5	28.8	81.7	11.3	2.7
0000184792	Paxlovid group	4306.2	1921.8	2384.4	249	5.8	126.5	122.5	90.8	35.7	39.5	7.3	75.7	34.2	14.5	27.6	14.6	0	14.2	10.6	11	11.6	16.6	11.3	6	1.3	27.3	2.4	16	14.9	15.1	48.6	142.2	47.7	10.5
0000190362	Paxlovid group	3234.6	1535.3	1699.4	1526.1	47.2	754.6	771.5	316	438.7	246.7	102.5	422.2	83	92	88.1	52.9	124.4	102.6	88.2	123.5	68.5	32.1	146.1	55.4	47.1	102.9	15.4	126.5	97.4	79.9	308.8	981.6	219.2	16.4
0000221283	Azvudine group	3488.9	1584.3	1904.6	20.5	0.6	6.5	14	3.7	2.8	0	0	14	0	0	3.7	0	0	0	2.4	0.5	0	0	0	0	0	0.2	0	1.1	5.7	7.1	5.1	14.8	0.6	0
0000246981	Paxlovid group	6181.2	2785.9	3395.2	0	0	0	0	0	0	0	0	0	0	0	0	0	0	0	0	0	0	0	0	0	0	0	0	0	0	0	0	0	0	0
0000264739	Paxlovid group	2988.7	1252.6	1736.2	744.2	24.9	339.1	405.1	153.2	185.8	143.7	28.8	232.6	52.2	41.8	36.1	23.1	38.4	45.2	55.9	46.4	33.6	57.6	52.5	18.6	10.2	72.3	2.2	51.9	60.1	46.1	71.5	335	277.6	60
0000270516	Paxlovid group	2566.9	1136.7	1430.2	764.1	29.8	411.4	352.7	174.5	236.9	138.3	23.5	190.9	52.7	62.4	26.7	32.7	36.3	56.7	51.8	92.2	44.7	42.5	51	7.3	16.2	50.4	9.6	19	39.4	72.5	125.5	516.5	114.9	7.2
0000280303	Paxlovid group	6043.4	2789.6	3253.8	0	0	0	0	0	0	0	0	0	0	0	0	0	0	0	0	0	0	0	0	0	0	0	0	0	0	0	0	0	0	0
0000291750	Azvudine group	2789	1288.3	1500.7	0	0	0	0	0	0	0	0	0	0	0	0	0	0	0	0	0	0	0	0	0	0	0	0	0	0	0	0	0	0	0
0000296831	Paxlovid group	2480.6	1082.3	1398.3	128.6	5.2	107.2	21.4	8.7	98.5	0	1.3	20.1	5.2	0.4	0	3.1	10.7	30.6	36.2	21	0	0	0	0	1.3	6.2	0.1	4.6	5.5	3.7	0.2	107.5	20.8	0.1
0000306054	Paxlovid group	4743.3	2188.8	2554.5	754.8	15.9	298.7	456.1	179.6	119.1	160.9	28.5	266.7	83.9	15.4	39.6	40.7	5.9	26.3	71	16	6.3	119.6	35	9.5	19	140.3	2.8	8.2	62.2	53.2	91	443.9	179.3	40.6
0000307654	Azvudine group	3800.4	1557.8	2242.6	336.5	8.9	152.4	184.1	90.6	61.8	34.2	13	136.9	9.1	0.7	19.5	61.3	1.2	23.3	10	27.3	0.2	10.5	23.4	3.2	9.8	49	8.3	15.1	32.1	32.4	78	205.5	42.7	10.2
0000308605	Paxlovid group	2386.2	1082.7	1303.5	215.4	9	85.7	129.7	3.8	81.9	8.4	31.6	89.7	3	0	0.1	0.8	6.8	9.8	36.7	28.5	0.7	3.7	4	27.9	3.7	16.7	10.8	10.4	30.1	21.7	38.8	96.1	56.9	23.7
0000325887	Azvudine group	4443.4	1995.3	2448.1	208.2	4.7	64.5	143.7	31.4	33.1	50.6	2.1	90.9	3.8	7.9	16.5	3.2	21.3	0	2.1	9.7	4.2	45.4	1	0.1	2	34.2	0.9	0.6	24.8	30.5	74.8	108.8	20.5	4.1
0000325980	Paxlovid group	4033.4	1949.7	2083.8	21.6	0.5	19.9	1.7	2.6	17.3	1.4	0	0.4	2.2	0	0	0.4	0	9.5	3	4.8	0	1.4	0	0	0	0.4	0	0	0	0	4	14.9	2.8	0
0000337873	Paxlovid group	3783.6	1605.4	2178.1	1436.6	38	754	682.5	256.7	497.3	132.5	37.1	513	59.5	50	40.5	106.8	34.4	127	177	158.8	12.4	43.2	76.9	13.5	23.6	130.9	18.1	126.8	182.1	55	92.8	642.8	584.7	116.2
0000341557	Paxlovid group	3781	1448.5	2332.5	550.4	14.6	521.4	29	479	42.5	1	5	23	163.3	164.6	88.1	63	18.4	1.3	5.9	16.8	0	0	1	0.7	4.3	2.8	0.3	0.2	11.1	8.5	129	239	133	49.5
0000370702	Paxlovid group	4002.4	1766.8	2235.6	42.3	1.1	11.4	30.9	2.4	9	7.4	0	23.4	2.4	0	0	0	5.3	0	0	3.8	0	7.4	0	0	0	1.5	7.1	11.4	2.3	1.1	2.1	29.2	10.6	0.4
0000427108	Paxlovid group	3248.8	1366.9	1882	77.9	2.4	30.6	47.3	18.8	11.7	17.5	6.1	23.8	3.5	5.4	7.7	2.3	0.1	6.5	4.8	0.4	3.9	12.6	1	5.6	0.5	11.4	0	6.3	4.6	1.4	15.6	44.1	16.5	1.8
1000057764	Paxlovid group	4223.1	1977.9	2245.1	1215.6	28.8	208.7	1006.8	108.7	100.1	530.5	129.3	347.1	64.9	7.1	15.6	21.2	6.4	23.9	25	44.7	146.7	202.9	180.8	106.4	22.9	105.3	8.8	85.6	88.4	59	89	808	304	14.5
1000091615	Paxlovid group	1878.6	999	879.6	17.2	0.9	1.1	16.1	0.3	0.8	0.4	0.4	15.4	0.1	0	0	0.1	0	0	0.6	0.3	0	0.4	0	0	0.4	0.8	2.2	2.1	4.3	5.9	2.6	4	5.9	4.8
1000098957	Paxlovid group	3393.9	1694.3	1699.6	393.3	11.6	38.9	354.4	10.9	28	13.2	4.9	336.3	4.7	0	0.1	6.1	6.2	5.8	12.3	3.6	0	13	0.2	3.5	1.4	102.6	47.5	80.6	85.7	20	82.9	241.8	51.4	17.2
1000102044	Paxlovid group	4867.8	2053.5	2814.3	770.9	15.8	215.4	555.5	78.3	137.1	217.3	62.1	276.1	11.2	36.1	23.9	7.2	33.1	32.1	30.4	41.6	60.7	134.3	22.3	35.7	26.5	86.2	5.3	47.8	96.2	40.6	351.2	336.5	61.5	21.6
1000108172	Paxlovid group	3513.5	1605.2	1908.3	15.2	0.4	3.1	12.2	3.1	0	7.2	5	0	0.4	2	0.6	0	0	0	0	0	0.8	0.3	6.1	0	5	0	0	0	0	0	0.2	13.5	1.5	0
1000121884	Paxlovid group	3355.2	894.7	2460.4	288.9	8.6	163.9	125	1.7	162.2	8.1	3.7	113.3	1	0	0.3	0.4	3.9	46.2	53.6	58.5	3.9	4	0.2	3.7	0	17.8	5.4	19.3	13.2	57.7	17.4	216.5	54.6	0.3
1000130965	Paxlovid group	4159.1	1884.9	2274.2	283.1	6.8	75.5	207.6	12.8	62.6	104.3	6.2	97.1	7.1	0	3.7	1.9	6.9	15.9	22.2	17.7	1.6	96.8	5.9	6.2	0	60.9	0	1.8	24.2	10.2	33.9	214.1	33.4	1.7
1000135966	Azvudine group	3293.6	1556.6	1737	994.6	30.2	582.9	411.7	245.9	337	97.9	113.7	200.1	90	74.8	59.6	21.5	33.6	99.7	97.8	105.9	36.1	30.3	31.4	82.3	31.4	61.9	17.4	55.7	38.6	26.5	271.6	293.4	266.3	163.4
1000169129	Paxlovid group	3313.3	1296.4	2016.8	889	26.8	413.4	475.6	158.1	255.3	104.6	10.8	360.2	82.8	5.9	27	42.4	55	59	93.1	48.3	7.1	96.5	1	4	6.8	118.4	8.7	53.5	96.2	83.4	79.2	374.5	339	96.3
1000255169	Paxlovid group	4710.4	1976.2	2734.2	1862.8	39.5	923.5	939.3	484.8	438.7	639.4	108.1	191.9	73.9	67.2	160.7	183	42.6	140.7	129.5	125.9	262.1	103.7	273.5	39.8	68.3	29.4	85.2	54.2	11.7	11.4	54.7	1070.6	735.6	1.9
1000273005	Paxlovid group	5602.4	2649.1	2953.2	62.6	1.1	12.3	50.3	0	12.3	13.6	24.8	11.9	0	0	0	0	0.3	0.1	4.7	7.3	0.7	1	11.8	0.7	24.1	11.4	0.5	0	0	0	13.8	44.4	4.4	0
1000281522	Paxlovid group	2393.3	995.6	1397.7	431.6	18	168.5	263.1	53.7	114.8	87.4	24	151.6	31.2	6	12	4.5	29.4	27.2	54.1	4.1	18.3	48.8	20.3	20.7	3.3	64.1	5.2	35.8	21.1	25.4	52.8	229.7	119.1	30.1
1000301548	Paxlovid group	5263.4	2420.9	2842.5	51.4	1	19.5	31.9	6.4	13.1	12.5	4.5	14.9	2.2	1	2.4	0.8	0.4	2.9	7.6	2.2	1.9	1.2	9.4	1.6	2.9	0.9	1.1	2.2	10.7	0	3.3	39.6	8.3	0.2
1000303107	Paxlovid group	1848.8	834.9	1013.8	700.5	37.9	388	312.5	168.5	219.5	89.6	45	177.9	63.2	42.1	34.7	28.4	45.2	60.8	69.6	43.8	13.7	18.1	57.7	24.3	20.7	42.6	7.7	49.2	57.7	20.8	20.2	490.4	182.7	7.2
1000316781	Paxlovid group	5918.7	2761.1	3157.6	39.7	0.7	2	37.7	1.4	0.6	6	28.1	3.6	0	0	0	1.4	0	0	0.6	0	3.5	2.5	0	17.6	10.5	0	0.4	0.2	2.1	0.9	4.1	32.5	3.2	0
1000321166	Paxlovid group	3751.6	1191.8	2559.7	1053.8	28.1	810.8	243	417.6	393.3	13.9	2.3	226.7	120.5	130.3	93.1	73.6	76.9	88.6	117.8	110.1	1.4	12	0.5	0.2	2.1	38.1	25.1	36.5	57.5	69.5	59.2	636	355.2	3.4
1000321265	Paxlovid group	5908.3	2782.1	3126.2	58.9	1	11.8	47.1	8.1	3.7	15.2	0.1	31.8	4.3	0	0	3.7	1.4	0.8	0.7	0.7	0	15.2	0	0.1	0	18.8	0	0	8.1	4.9	2.5	44.3	12.1	0
1000357266	Paxlovid group	3735.4	1679.3	2056.1	127.7	3.4	40	87.7	2.2	37.8	5.8	5.8	76.1	1.8	0	0.1	0.3	1	2.8	15.6	18.4	0.8	0.7	4.4	1.3	4.5	5.8	3.7	7.9	38.1	20.6	7.7	72.2	44.4	3.4
1000383224	Paxlovid group	2544.4	1387.3	1157.1	958.1	37.7	351.7	606.3	123.9	227.8	212.3	144.6	249.4	44.3	13.7	26.6	39.3	44.2	29.1	79.2	75.3	23.3	102.5	86.4	69.9	74.7	58.1	7.4	51.1	93.6	39.2	40.8	710.6	200.6	6.1
1000452970	Azvudine group	2090.8	1018.2	1072.7	946.6	45.3	402.5	544.1	201.5	201	258.4	57.1	228.5	93	27.2	43	38.3	42.6	38.1	71.4	48.9	99.5	101.6	57.3	19.9	37.2	76.2	23.5	62.2	52.2	14.4	109.4	426.4	317	93.8
1000454501	Paxlovid group	4064.4	2611.2	1453.2	15	0.4	0	15	0	0	0.7	11.1	3.2	0	0	0	0	0	0	0	0	0	0.4	0.3	2.1	9.1	1.7	0	0	0	1.4	2.3	10.3	2.4	0
1000519287	Azvudine group	5152.7	2402.1	2750.5	222.7	4.3	103.4	119.3	61.4	42	58.9	9.4	51	10.1	37.8	9	4.5	11.7	8.1	11.3	10.9	11	12.2	35.7	7.1	2.2	8.2	6.7	18.1	6.1	11.9	40.4	159.4	20.8	2.1
1000538607	Paxlovid group	1986.7	939.8	1047	790.3	39.8	318.8	471.4	167.5	151.3	116.6	44	310.8	45.5	30.3	47.7	44	59.4	52.6	26.2	13.1	37.4	54.3	24.9	28.2	15.8	68.6	35.5	71.9	85.4	49.5	1	342.8	436.7	9.8
1000587722	Paxlovid group	2779.9	1224.5	1555.5	600	21.6	382.3	217.7	121.2	261.1	31.6	30.6	155.5	37.4	17.6	21.4	44.8	22.2	88.8	84.4	65.7	6.7	9.8	15.1	5.7	24.9	59.5	8.2	30	35	22.8	13.1	247.4	299.1	40.3

1000587722	Paxlovid group	2779.9	1224.5	1555.5	600	21.6	382.3	217.7	121.2	261.1	31.6	30.6	155.5	37.4	17.6	21.4	44.8	22.2	88.8	84.4	65.7	6.7	9.8	15.1	5.7	24.9	59.5	8.2	30	35	22.8	13.1	247.4	299.1	40.3
1000625574	Azvudine group	4002.4	1964.3	2038.1	790.8	19.8	210.3	580.5	99.9	110.4	246.1	121.7	212.7	19.6	37.9	13.4	29	48.8	30.4	25.3	5.9	69	107.9	69.2	74.7	47	81	0.1	69.8	38.7	23.1	30.5	490.9	263	6.4
1000674022	Paxlovid group	5285.7	2223.9	3061.8	57.8	1.1	32.1	25.7	1.4	30.7	2.6	4.7	18.3	0.2	0	0.4	0.7	2.7	3.4	22.2	2.4	0.3	0.1	2.3	2.5	2.3	0.3	1.8	8.7	6.4	1.2	15.7	32.9	8.2	1
1000743528	Paxlovid group	2287.5	843.4	1444.2	275.1	12	81.1	194	31.2	49.9	104	34.7	55.3	11.3	4.9	4.4	10.5	1.8	22.1	15.7	10.3	63.7	30.2	10	21.8	13	1.4	5.4	47.5	1	0	12.8	145.5	113.4	3.4
1000783638	Paxlovid group	4169.7	1944.9	2224.8	64.2	1.5	26.8	37.5	18.5	8.3	0.9	7.5	29.1	4.5	7.9	2.6	3.5	0.4	3.4	3.5	0.9	0	0.7	0.2	0.3	7.2	0.4	7.6	11.7	1.5	7.9	21.3	28.5	9.7	4.8
1000833034	Paxlovid group	3513.2	1771	1742.1	202.5	5.8	43	159.5	4.8	38.2	44.5	27	87.9	0.4	1.7	0	2.6	0.4	3.2	6.2	28.4	5.9	38.2	0.5	18.9	8.1	17.3	8.4	7	2.7	52.6	35.9	93.6	56	17
1000843573	Paxlovid group	3105.1	1269.1	1836	46.9	1.5	20.7	26.3	17.2	3.4	8.5	5.3	12.5	7.5	7.4	0.2	2.1	0	0.3	2.8	0.3	0	0.8	7.7	4.9	0.4	8.2	0.2	1.9	0.2	1.8	11.7	23.4	9.7	2.2
1000845317	Azvudine group	4829.4	2303.1	2526.3	0	0	0	0	0	0	0	0	0	0	0	0	0	0	0	0	0	0	0	0	0	0	0	0	0	0	0	0	0	0	0
1000894449	Paxlovid group	3516.7	1546.3	1970.4	205.2	5.8	83.3	121.9	30.1	53.2	25.4	35.6	60.9	22.3	6.6	0.5	0.8	18.9	9	11.8	13.5	4.1	14.4	7	24.8	10.8	24.3	0	24	10.9	1.6	10	186.8	8.4	0
1000901328	Paxlovid group	2651.2	1192	1459.2	438.3	16.5	167.9	270.5	15.7	152.2	82.5	4.8	183.2	12.8	0	1.5	1.4	20.3	50.8	45.6	35.5	4.4	77.2	0.9	2.4	2.4	39.8	13.4	27.4	61	41.5	40.1	255.8	125.7	16.7
1000931768	Azvudine group	2849.4	1295.6	1553.8	10.3	0.4	6.4	3.8	5.5	0.9	0	3.8	0	0	0	0	5.5	0	0.9	0	0	0	0	0	2.8	1	0	0	0	0	0	3.9	6.1	0.2	0
1000938014	Paxlovid group	3236.1	1424.5	1811.6	270.7	8.4	17.4	253.3	6.1	11.3	154.3	38.5	60.5	0	4.7	0.6	0.8	0.1	2	2.2	7.1	34.8	61.3	58.2	27.5	11	9.3	1.5	20.3	15.3	14.2	9.6	206.2	52.3	2.5
1000963479	Paxlovid group	4020.3	2032.2	1988.1	617.2	15.4	89.4	527.8	42.3	47.1	214.7	42.5	270.6	0.7	16.8	22	2.8	0	17.1	15.2	14.9	42.1	91.3	81.3	11.9	30.5	91	7.2	35.6	39.1	97.8	87.4	366.1	137.4	26.3
1001017808	Azvudine group	4819.1	2180.1	2638.9	1936.9	40.2	1013.7	923.2	604.4	409.3	364.7	248.2	310.3	178.8	201.9	134.9	88.9	97.8	117.5	99.7	94.3	105	88.3	171.4	150.3	97.9	59.2	22.9	99.2	80.3	48.8	212.4	1410.9	299.9	13.8
1001028235	Paxlovid group	3982.3	1859.3	2122.9	788.8	19.8	240.8	548	181.2	59.6	299.1	71.2	177.7	33.4	63.6	60.4	23.8	36.9	1.9	9.6	11.2	81.3	121.5	96.3	26	45.2	74.3	3.8	35.7	45.9	18	115.7	590.7	79.8	2.5
1001069844	Azvudine group	4703.9	2180.7	2523.2	118.5	2.5	83.6	34.9	0.2	83.4	0.9	0.2	33.7	0.2	0	0	0	66.9	3.9	2.6	10	0	0.7	0.2	0.2	0	19.8	0	1	7.6	5.2	54.3	50.3	9.9	4
1001070557	Paxlovid group	3724	1518.3	2205.7	37.5	1	17.7	19.8	9.1	8.6	0.4	0	19.4	7.1	0	0	2	2.9	1.8	1.6	2.3	0	0.4	0	0	0	10.7	4.8	0	0.2	3.7	10.1	22.9	4.2	0.2
1001090609	Paxlovid group	3272.9	1478.2	1794.8	1166.6	35.6	458.8	707.8	152.8	306.1	147.7	98.9	461.2	33.9	17.3	49.6	52	49.8	73.8	87	95.5	19.4	76	52.3	71.9	27	111.2	26.5	77.6	134	112	55.8	513.6	530.3	66.8
1001143616	Paxlovid group	4002.3	1617.9	2384.3	349.9	8.7	138.9	211	49.1	89.8	110	18.9	82.1	28.5	0.8	8.3	11.5	25.8	11	34.8	18.2	18.7	58.7	32.6	18.1	0.8	1.6	17.2	58.4	3.6	1.2	7.6	126.7	187.8	27.8
1001156030	Paxlovid group	3374.5	1590.2	1784.3	61.4	1.8	7.5	53.9	4.8	2.7	0.2	3.8	49.9	0	4.3	0.2	0.3	2.4	0	0	0.3	0	0.2	0	0	3.8	30.9	2.6	0.2	4.7	11.6	4.6	30.1	24.4	2.3
1001237355	Azvudine group	3343	1704.9	1638.1	21.6	0.6	18.2	3.4	2	16.2	0	1	2.4	0	0	0	2	0.3	1.8	3.2	10.9	0	0	0	0	1	1	0.2	0	0	1.2	0.3	11.2	9.7	0.4
1001257003	Azvudine group	2697.6	1187.7	1509.8	735	27.2	340.9	394.1	88.1	252.9	99.5	27.8	266.8	6.5	23	25	33.6	44.9	50.6	92.1	65.3	10.3	45.8	43.4	5.4	22.4	56.5	26	48.4	65.4	70.5	61.6	306.6	287.2	79.6
1001301785	Azvudine group	4205	1636.5	2568.5	968.8	23	344.5	624.4	170.8	173.6	283.8	27.6	313	86.8	17.6	42.3	24.1	52.3	31.9	57.5	31.9	113.9	141.7	28.2	21.8	5.8	48.9	13.4	62.4	99.3	88.9	146.7	489	267.9	65.2
1001360064	Paxlovid group	3701.4	1804.1	1897.3	270.1	7.3	55.4	214.6	2.5	52.9	5.7	2.1	206.9	0	0	0	2.5	20.8	0	15.2	16.9	0.2	5.5	0	1.8	0.3	28.9	12.2	20.1	77.5	68.1	107.8	138.5	18.8	5
1001360893	Paxlovid group	4947.5	2318.3	2629.2	0	0	0	0	0	0	0	0	0	0	0	0	0	0	0	0	0	0	0	0	0	0	0	0	0	0	0	0	0	0	0
1001383651	Azvudine group	2652.9	1207.1	1445.8	1347.6	50.8	477.2	870.4	275.7	201.5	396.1	148	326.3	94	84.9	30	66.8	41.6	49.5	55.3	55	179.5	150.7	65.9	90.9	57.1	67.9	36.4	60	95.8	66.3	24.8	731	558.8	33
1001418411	Azvudine group	2780.8	1165	1615.8	890.6	32	408.3	482.3	235.6	172.7	287	39.8	155.4	28.6	44.8	80.8	81.4	19.1	42.3	58.5	52.8	35.6	67.4	184	16.4	23.5	33.3	10.9	15.7	42.9	52.7	15.5	582.4	282	10.7
1001433297	Paxlovid group	2058.7	1014.9	1043.8	680.1	33	164.4	515.7	76.5	87.9	244.5	91	180.2	37.5	17.1	7	14.9	7	27.9	13.8	39.1	45.8	141.1	57.5	59.5	31.5	38	12.2	47.8	28.8	53.4	55.7	522	95.7	6.6
1001480398	Azvudine group	3106.1	1473.3	1632.9	141.2	4.5	52	89.1	38.3	13.8	48.9	1.6	38.6	16.3	10.2	6.1	5.6	6.7	0.8	0.7	5.5	3.6	36.3	9	1.5	0.1	22.9	0	3.1	5.9	6.7	59.5	60.9	15.6	5.1
1001481703	Paxlovid group	3685.7	1857.6	1828.1	79.6	2.2	7.1	72.6	4.3	2.8	41	4.6	27	0	3.7	0.2	0.3	1.7	0	0	1.1	2.9	28.9	9.3	4.2	0.4	8.2	0	18.2	0.4	0.1	3.9	36.3	36.7	2.7
1001493057	Azvudine group	3276.6	1468.9	1807.8	0	0	0	0	0	0	0	0	0	0	0	0	0	0	0	0	0	0	0	0	0	0	0	0	0	0	0	0	0	0	0
1001493129	Azvudine group	2875.5	1312.8	1562.7	6.2	0.2	0	6.2	0	0	5.6	0.5	0	0	0	0	0	0	0	0	0	0.2	0	5.4	0.5	0	0	0	0	0	0	0.7	5.2	0.2	0
1001497739	Paxlovid group	4832.8	2064.8	2768	15.1	0.3	14.1	1	0	14.1	0	1	0.1	0	0	0	0	0	7.8	0.8	5.5	0	0	0	1	0	0	0	0.1	0	0	1	9.4	4.7	0
1001497739	Paxlovid group	4832.8	2064.8	2768	15.1	0.3	14.1	1	0	14.1	0	1	0.1	0	0	0	0	0	7.8	0.8	5.5	0	0	0	1	0	0	0	0.1	0	0	1	9.4	4.7	0
1001498698	Paxlovid group	2633.1	1098.1	1535	1058.5	40.2	231	827.4	120.4	110.6	362.1	179.3	286	43.9	37.3	20.1	19.1	1.4	43.9	42.5	22.8	137.2	91.5	133.4	60.2	119.1	95.4	24.3	34	63.9	68.5	23.2	482.9	542.6	9.8
1001498850	Azvudine group	3461.8	1702.8	1759	7.4	0.2	0	7.4	0	0	0	0	7.4	0	0	0	0	0	0	0	0	0	0	0	0	0	1.8	0	0	0	5.6	2.1	5.4	0	0
1001499027	Paxlovid group	2078.7	916.1	1162.7	331.8	16	259.6	72.1	65.5	194.1	6.6	7.8	57.8	14.3	2.7	16.6	32	28.8	56.1	66.2	43	0	6.6	0	1.9	5.8	6.7	5.4	0.4	27.3	18	2.4	213.2	113.3	2.9
1001499070	Azvudine group	7956.2	3900.7	4055.5	80.4	1	19.7	60.8	2.4	17.3	22.1	1.7	37	0	0	0.6	1.8	4.2	0	3.3	9.7	7.4	14.6	0.1	1.7	0	1.5	8.5	8.6	4.6	13.7	21	44.1	13.1	2.2
1001499192	Azvudine group	3988.1	1766.9	2221.3	93.6	2.3	39.7	53.9	9.1	30.6	6.6	0	47.3	4.1	1.1	2	1.9	1.2	1.6	15.1	12.6	0.7	4.1	1.8	0	0	13.1	0	1.6	16.2	16.5	25.8	66.4	1.4	0
1001499299	Azvudine group	4722.7	2407.8	2314.9	13.7	0.3	0	13.7	0	0	13.7	0	0	0	0	0	0	0	0	0	0	0	13.7	0	0	0	0	0	0	0	0	0.7	9.8	3.1	0.1
1001499300	Azvudine group	4178.7	1874.7	2304	45.7	1.1	27.7	18	8.2	19.5	9.9	0.6	7.5	3.7	0	0	4.5	0.5	17.5	0.2	1.3	0	9.1	0.7	0.6	0	1.8	0	0	2.6	3.1	1	37.5	7.2	0.1
1001499316	Paxlovid group	2552.8	1116	1436.8	199.3	7.8	5	194.3	0.8	4.2	71.9	21.9	100.5	0	0	0	0.8	0.3	1.6	0.8	1.5	6.5	47.5	17.9	11.5	10.4	47.7	0	4.8	15.2	32.8	7.9	121.3	64.9	5.3
1001499325	Paxlovid group	5884.9	2618.4	3266.5	7.2	0.1	1.2	6	0.4	0.9	0.9	0.8	4.4	0	0	0	0.4	0	0	0	0.9	0.5	0.3	0	0.3	0.5	0	0	2.7	0.8	0.8	3.7	3	0.5	0.1
1001499353	Azvudine group	2009	871.6	1137.3	54.1	2.7	13.8	40.3	6.8	7	20	0	20.3	0	0.5	0.1	6.1	0	1.7	1.9	3.5	6.6	9.1	4.3	0	0	4.8	0.1	0.4	5.6	9.3	1.1	39.7	12.8	0.5
1001499356	Paxlovid group	3370.8	1416.6	1954.2	1275.7	37.8	291.4	984.4	15	276.4	52	116.3	816	0	0	1.4	13.6	15.9	71.6	93.6	95.3	2	48.8	1.2	52.9	63.5	110.4	85.9	169.6	207.4	242.7	76.8	671.1	520.8	7
1001499359	Azvudine group	2315.9	968.5	1347.4	96.9	4.2	57.8	39.1	0	57.8	5.5	0.6	33	0	0	0	0	7.2	7.1	18	25.5	0	5.5	0	0	0.6	5.2	2.6	2.3	1.8	21	4.3	63.5	28.5	0.5
1001499366	Azvudine group	4441.5	1924.3	2517.2	69.3	1.6	29	40.3	0.2	28.8	2.4	3.8	34.2	0	0	0	0.2	13	5.4	3.6	6.8	0	2.4	0	3.8	0	6.2	0	11.3	14.4	2.3	12	51.6	5.6	0.1
1001499550	Paxlovid group	2674.1	1189.4	1484.7	57.7	2.2	2.8	54.9	0	2.8	7.2	1.2	46.5	0	0	0	0	0	1.4	0	1.3	0	5.1	2.1	0.3	1	17.6	0.4	9.3	8.2	11	1.3	44.9	11.3	0.1
1001499554	Paxlovid group	2919.2	985	1934.2	247.4	8.5	55.5	191.9	31.7	23.8	97	2	92.9	0.6	8.7	7	15.4	0.3	15.4	7.3	0.8	14.4	66.5	16.1	1.6	0.4	85.6	0.4	0.1	3.3	3.5	51	147.2	42.5	6.7
1001499589	Paxlovid group	5508.3	2684.6	2823.7	58.1	1.1	55.1	3	16.8	38.3	0.2	0	2.7	15.3	0	0	1.5	2.1	0	13.5	22.7	0.2	0	0	0	0	0.4	0	0.6	0.7	1.1	29.1	25	3.2	0.8
1001499606	Paxlovid group	4609	2300.4	2308.6	57.4	1.2	31	26.3	0.5	30.5	0.8	0	25.5	0	0	0.5	0	0	8.1	15	7.4	0	0.8	0	0	0	4.6	0.4	1.8	9.2	9.4	1.1	52.9	3.4	0
1001499626	Azvudine group	4194.5	1844	2350.6	127	3	42.4	84.7	14.1	28.3	41.6	4.8	38.3	5.3	7	1.8	0	1.6	22.7	3.2	0.8	14.8	16.1	10.7	4.8	0	18.8	3.6	3.2	6.4	6.3	8.2	91.2	26.2	1.4
1001499887	Azvudine group	5657.3	2624	3033.3	158.9	2.8	38.7	120.2	37.9	0.7	89.3	21.2	9.7	31.4	5.9	0.6	0	0.3	0	0.2	0.2	8.8	56.4	24.2	21.2	0.1	3.8	0.5	4.5	0.5	0.4	44.1	59.1	41.1	14.6
1001499895	Azvudine group	3127	2073.5	1053.6	256.3	8.2	231	25.2	82.9	148.1	0	0.7	24.6	15.3	23.1	10.1	34.3	44.3	19.9	34	50	0	0	0	0.6	0.1	12.1	0.2	0.3	10.3	1.7	37.1	132	75.8	11.4
1001499898	Azvudine group	2890.9	1298.3	1592.6	17.2	0.6	1.5	15.6	0	1.5	7.9	4.1	3.7	0	0	0	0	0	1.5	0	0	0	7.3	0.6	0.7	3.4	2.4	1.2	0	0	0	0.6	12.3	4.3	0.1
1001499919	Azvudine group	2928.3	1378.5	1549.8	13.5	0.5	4.3	9.2	0.5	3.8	0.1	0	9.1	0.5	0	0	0	0	0	2	1.8	0	0.1	0	0	0	4.9	0	0	1.6	2.5	1.1	10.3	2	0.1
1001499924	Azvudine group	3611.8	1730.8	1881	220.2	6.1	28.4	191.8	7.4	21	36.7	36.6	118.5	5.4	2	0	0	0	11.8	4	5.2	12	3.5	21.1	31.8	4.8	35.2	10.4	13.8	34.3	24.8	37.5	133.9	43.1	5.8
1001500146	Azvudine group	3926.8	1863.4	2063.5	11.6	0.3	8.5	3.1	0	8.5	0	0	3.1	0	0	0	0	1.6	0.1	1.8	5	0	0	0	0	0	0	0.9	0	1.4	0.8	4.6	4.3	1.8	1
1001500188	Paxlovid group	3769	1729.7	2039.3	0	0	0	0	0	0	0	0	0	0	0	0	0	0	0	0	0	0	0	0	0	0	0	0	0	0	0	0	0	0	0

1001500204	Azvudine group	3694.7	1707.4	1987.3	0	0	0	0	0	0	0	0	0	0	0	0	0	0	0	0	0	0	0	0	0	0	0	0	0	0	0	0	0	0	0
1001500205	Azvudine group	4123	1942.8	2180.1	157.6	3.8	30.2	127.4	1.5	28.6	5.4	0.9	121.2	0.8	0	0	0.7	17.9	5.9	4.1	0.7	0	5.1	0.3	0.4	0.5	67.2	0.6	18.6	9.4	25.4	23	121.4	12.7	0.4
1001500296	Azvudine group	3944.5	1698.4	2246	21.9	0.6	9.3	12.6	3.3	6	0.8	0	11.8	0	0	0	3.3	0	5.7	0.1	0.2	0	0.6	0.3	0	0	0	0	9.5	2.3	0	6.7	11.3	3.4	0.4
1001500434	Paxlovid group	5166.8	2381.4	2785.5	0	0	0	0	0	0	0	0	0	0	0	0	0	0	0	0	0	0	0	0	0	0	0	0	0	0	0	0	0	0	0
1001500672	Azvudine group	3420.6	1489.9	1930.7	0	0	0	0	0	0	0	0	0	0	0	0	0	0	0	0	0	0	0	0	0	0	0	0	0	0	0	0	0	0	0
1001500751	Paxlovid group	5539.2	2367.3	3171.9	0	0	0	0	0	0	0	0	0	0	0	0	0	0	0	0	0	0	0	0	0	0	0	0	0	0	0	0	0	0	0
1001500755	Azvudine group	4313.5	1838.4	2475.1	0	0	0	0	0	0	0	0	0	0	0	0	0	0	0	0	0	0	0	0	0	0	0	0	0	0	0	0	0	0	0
1001500756	Azvudine group	5068.5	2712.7	2355.8	20.3	0.4	0	20.3	0	0	0.5	0.1	19.7	0	0	0	0	0	0	0	0	0.1	0	0.4	0.1	0	2.4	1.3	6.5	2.5	7	2.5	14.4	3.2	0.2
1001500885	Paxlovid group	4250.4	1999.9	2250.5	265.4	6.2	66.8	198.6	63.6	3.1	84.8	7.2	106.7	0.7	54.9	7.6	0.4	1.9	0	0	1.2	12	55.2	17.6	6.7	0.4	33	0	17	35.8	20.9	34.3	185.8	44.7	0.6
1001501102	Paxlovid group	3058.7	1217.1	1841.7	160.6	5.3	16.4	144.2	3.4	13.1	13.7	55.3	75.2	0	0	0	3.4	1.3	3.3	6.5	2	3.5	7.4	2.8	21.9	33.3	11.3	9.1	9.2	16.7	28.8	18.9	95.7	40.8	5.2
1001501119	Azvudine group	3909.8	1888.3	2021.6	0	0	0	0	0	0	0	0	0	0	0	0	0	0	0	0	0	0	0	0	0	0	0	0	0	0	0	0	0	0	0
1001501160	Paxlovid group	2473.7	1132.1	1341.6	775.4	31.3	341.9	433.5	134.2	207.7	124.7	78.3	230.5	39.5	28.4	25.2	41.1	41.1	54.5	57.3	54.8	15.5	63.9	45.3	37.9	40.4	47.9	21.4	38.2	67	56	24.2	539.8	206.3	5
1001501335	Azvudine group	3013.8	1146.7	1867.1	117.1	3.9	58.3	58.9	2.8	55.5	39.8	10.8	8.2	2	0	0	0.8	23.3	1.1	0.4	30.7	17	22.8	0	5.8	5	5	0.1	2.8	0.3	0	0.5	30.7	80	6
1001501339	Paxlovid group	2308.5	1093.5	1215	31.6	1.4	0.8	30.8	0	0.8	1	3.2	26.6	0	0	0	0	0	0	0	0.7	0	1	0	3.2	0	0.5	4.6	1.5	4.3	15.7	0.8	21.3	8.5	1
1001501384	Azvudine group	2731.4	1155.1	1576.3	55.4	2	22	33.4	1.1	21	3.6	15.3	14.5	1.1	0	0	0	0.1	2	3.4	15.5	0.5	2.2	0.9	13.4	1.9	0.5	8.2	0.6	2.2	3.1	2.1	41.3	11.2	0.7
1001501600	Azvudine group	3873.7	1870.4	2003.4	0	0	0	0	0	0	0	0	0	0	0	0	0	0	0	0	0	0	0	0	0	0	0	0	0	0	0	0	0	0	0
1001501603	Paxlovid group	5295.1	2039.8	3255.3	163.3	3.1	101.3	61.9	8.5	92.9	16.9	0	45	0	0	0	8.5	0.4	13.1	37.8	41.6	0	0	16.9	0	0	0	7.5	0	1.1	36.5	10.3	108.3	44.1	0.6
1001501667	Paxlovid group	3070.2	1462	1608.2	7.6	0.2	3	4.5	0.4	2.7	0.2	0	4.3	0	0	0	0.3	1.5	0	0	1.1	0	0.2	0	0	0	2	0	0.8	0.5	1	0.2	5.4	1.9	0
1001501731	Paxlovid group	1927	915.7	1011.3	117.5	6.1	29.1	88.4	18.8	10.3	2.9	23.3	62.3	0.4	4.7	9.3	4.4	2.5	2.1	2.2	3.5	0.6	2.3	0	14.6	8.7	4.4	6.7	8.5	15.1	27.7	3.3	86.4	26.1	1.6
1001501797	Azvudine group	4222.5	1858.3	2364.2	223.6	5.3	158	65.6	12.5	145.5	6.2	26.8	32.6	0.3	0	0.9	11.3	0	14.3	82.5	48.8	0.9	2.6	2.7	6.5	20.3	2.7	1.9	0	15	13	15.1	158.9	45.4	4.1
1001501826	Azvudine group	4042.5	1952.9	2089.6	0	0	0	0	0	0	0	0	0	0	0	0	0	0	0	0	0	0	0	0	0	0	0	0	0	0	0	0	0	0	0
1001501918	Azvudine group	4871.7	2298	2573.6	0	0	0	0	0	0	0	0	0	0	0	0	0	0	0	0	0	0	0	0	0	0	0	0	0	0	0	0	0	0	0
1001501978	Azvudine group	3102.8	1304.4	1798.3	124.2	4	104.3	20	8.7	95.6	2.9	1.1	15.9	3.5	0.2	2.1	3	45	1.5	28.3	20.7	0	2.9	0	0.1	1	0.3	0.7	10.2	2.4	2.3	0.6	50.4	72.8	0.4
1001501989	Paxlovid group	3056.9	1336.9	1720.1	455.7	14.9	267.2	188.4	108.6	158.6	81.6	10.4	96.4	69.7	4.7	13.1	21.1	56.9	7.9	64.2	29.7	3.1	76.7	1.8	0	10.4	46	0.5	0.2	31.1	18.5	7.6	203.7	216.9	27.5
1001502063	Paxlovid group	2602	1207	1395	34.6	1.3	4.3	30.3	0.5	3.8	1.8	13.9	14.6	0	0	0.5	0	0	0	1.5	2.3	0	0.2	1.6	13.9	0	2.3	0.8	0.1	3.6	7.8	3.6	27.4	3.6	0
1001502224	Paxlovid group	3612.8	1588.3	2024.5	62.5	1.7	10.1	52.4	8	2	8.9	2.3	41.2	2.5	2.6	2.5	0.5	0.9	0.1	0.2	0.9	3.8	4.4	0.7	0.7	1.6	10.2	4	5.5	9.5	12.1	7.9	41.3	12.1	1.2
1001502311	Paxlovid group	5401.4	2456.9	2944.5	43.2	0.8	11.6	31.6	1.2	10.4	0	0.5	31.1	0.6	0.1	0.5	0	0.4	0	4.6	5.5	0	0	0	0.5	0	0.5	0.6	7.9	12	10	4.2	37.8	1.3	0
1001502315	Azvudine group	4204.4	1960.6	2243.8	0	0	0	0	0	0	0	0	0	0	0	0	0	0	0	0	0	0	0	0	0	0	0	0	0	0	0	0	0	0	0
1001502338	Paxlovid group	6370.3	3153.1	3217.2	200.2	3.1	50.7	149.5	20.7	30	113.8	1	34.7	20.7	0	0	0	8.2	3	3	15.8	24.4	89.3	0	0	1	5.3	0	5.6	15	8.8	19.2	146.8	31.5	2.6
1001502341	Paxlovid group	4610.8	2114.1	2496.7	0	0	0	0	0	0	0	0	0	0	0	0	0	0	0	0	0	0	0	0	0	0	0	0	0	0	0	0	0	0	0
1001502382	Paxlovid group	3114.7	1278	1836.8	4.8	0.2	2.1	2.7	0	2.1	0.6	0	2.1	0	0	0	0	0.3	0	0	1.8	0.2	0	0.4	0	0	1	0	0.9	0.1	0	0.5	3.7	0.6	0
1001502848	Paxlovid group	1467.4	910.5	556.9	237	16.1	31.4	205.6	1.3	30.1	133.6	61.3	10.7	1.3	0	0	0	9	0	0.3	20.7	50.1	0.1	83.4	30.9	30.4	0	1.5	9.2	0	0	10.9	174.7	51.3	0.2
1001502864	Paxlovid group	3077.9	1422.7	1655.2	0	0	0	0	0	0	0	0	0	0	0	0	0	0	0	0	0	0	0	0	0	0	0	0	0	0	0	0	0	0	0
1001502867	Paxlovid group	3303	1517.5	1785.6	7	0.2	4.4	2.6	0	4.4	2.6	0	0	0	0	0	0	0	2.1	2.3	0	0	0	2.6	0	0	0	0	0	0	0	0.1	5.4	1.5	0
1001503182	Paxlovid group	4020.6	1803.8	2216.8	309.1	7.7	171.2	138	55.8	115.4	15.4	1.3	121.2	34.3	3.1	13.3	5.1	27.4	4.3	36.6	47.1	0	11.8	3.7	0.8	0.5	69.6	3.1	1.1	9.3	38.2	79.2	178.6	46.8	4.4
1001503388	Paxlovid group	5260.9	2559.5	2701.4	6.6	0.1	0.6	6	0	0.6	0.5	0	5.5	0	0	0	0	0	0.6	0	0	0	0.5	0	0	0	0	0	2	3.2	0.2	0.6	5.4	0.6	0
1001503603	Azvudine group	2756.5	1241	1515.5	438.6	15.9	182.7	255.9	30.8	151.9	67.8	23.6	164.5	7.5	0.1	1.3	21.9	30.3	51.5	44.3	25.7	0.5	64.1	3.1	22	1.6	44.3	4.4	30.7	52.8	32.4	20.4	303.6	114.3	0.2
1001503671	Paxlovid group	2800.5	1156.3	1644.2	194.9	7	28.9	166.1	1.4	27.5	11.3	0.9	153.9	1.4	0	0	0	4	0.4	17	6.1	0.3	9.9	1	0.9	0	62.5	0	4.6	52.6	34.1	12	90.3	68.6	24.1
1001503713	Paxlovid group	2680.6	1109.9	1570.7	827.3	30.9	348.1	479.2	100.4	247.7	145.7	77.5	256	31.1	17.4	45.1	6.8	37.5	42.6	110	57.6	37.6	40.8	67.2	57.5	20	77.1	13.6	43.3	78	43.9	81.4	571.5	172.1	2.3
1001503716	Azvudine group	3077.3	1470.7	1606.6	0	0	0	0	0	0	0	0	0	0	0	0	0	0	0	0	0	0	0	0	0	0	0	0	0	0	0	0	0	0	0
1001504262	Paxlovid group	3447.7	1482	1965.7	12.8	0.4	7	5.8	0	7	0.7	1.5	3.6	0	0	0	0	0	4	2.5	0.5	0	0.7	0	1.4	0.1	0	0	2.6	0.9	0	0.1	8.8	3.9	0
1001504450	Paxlovid group	3608.4	1939.1	1669.4	85.5	2.4	6.9	78.6	2.2	4.7	3.7	0	74.9	0	0	1.9	0.3	0	4.7	0	0	3	0.7	0	0	0	22.8	0.9	1.5	15.9	33.8	4.4	31.9	49.1	0.2
1001504454	Azvudine group	5120.4	2404.9	2715.6	0	0	0	0	0	0	0	0	0	0	0	0	0	0	0	0	0	0	0	0	0	0	0	0	0	0	0	0	0	0	0
1001505002	Paxlovid group	2339.2	114.5	2224.8	160	6.8	18	142	3.6	14.4	41.7	26.4	73.9	3.5	0	0	0	5.3	0	4	5.2	8.5	13	20.2	6.3	20.1	3.6	17.1	23.7	11.9	17.7	13.6	95.1	43.9	7.5
1001505028	Paxlovid group	4314.1	1960.1	2354	0	0	0	0	0	0	0	0	0	0	0	0	0	0	0	0	0	0	0	0	0	0	0	0	0	0	0	0	0	0	0
1001505303	Paxlovid group	4583.4	2185.1	2398.4	0	0	0	0	0	0	0	0	0	0	0	0	0	0	0	0	0	0	0	0	0	0	0	0	0	0	0	0	0	0	0
1001506272	Azvudine group	3129.1	1454.6	1674.5	768.9	24.6	313.9	455	28.7	285.2	62	25.8	367.2	9.2	7.4	7.5	4.6	70.2	45	70.8	99.2	8.3	48	5.7	14.2	11.6	99.2	31	47.9	96.1	92.9	9.6	584.1	174.6	0.6
1001506357	Paxlovid group	4112.3	1863.4	2248.9	81.8	2	20.4	61.4	0	20.4	0	38.8	22.5	0	0	0	0	19.9	0	0	0.5	0	0	0	24.1	14.7	0	9	7	1.9	4.7	53.1	23.9	4.2	0.6
1001506402	Paxlovid group	2513.8	1032.8	1481	98.4	3.9	5.3	93.1	1.7	3.6	5.6	10.6	76.9	0	0.1	1.6	0	0.3	2.3	0.2	0.8	0.8	2	2.8	0.1	10.5	18.7	2.4	4.5	2	49.4	19.4	67.5	11	0.5
1001506482	Paxlovid group	5762.2	2625.9	3136.3	132.4	2.3	22.3	110	9.6	12.8	56.6	11.8	41.6	0	0.8	3.8	4.9	0.2	11.4	0.6	0.6	5.9	28.2	22.5	8.8	3	13.7	0.5	5.8	12.2	9.4	58.2	54.1	16.7	3.3
1001506497	Paxlovid group	5859.6	2691.3	3168.3	120.3	2.1	4.3	116	2.9	1.4	21	22.6	72.3	0	0.7	1.2	1	0	0	0	1.4	8.5	4.9	7.5	14.4	8.2	22.3	3.7	27.5	9.3	9.6	28.3	72.4	17.6	1.9
1001506610	Azvudine group	3913.1	1745.3	2167.8	526.8	13.5	257.2	269.6	90.2	167	80.4	8.4	180.8	38.6	8	17.5	26.1	31.8	26.7	35.6	72.8	5.5	56.9	18	4.5	4	64.5	31.2	34.7	12.4	37.9	167.6	288.1	64.2	6.9
1001506704	Paxlovid group	4748.2	2116.9	2631.3	30.4	0.6	1	29.4	0.3	0.7	8.2	0	21.2	0	0	0.1	0.3	0	0	0.2	0.4	0	7.9	0.2	0	0	4.7	1	0	1.9	13.6	16.5	11.3	2.3	0.2
1001506752	Paxlovid group	3526.9	1474.2	2052.7	642	18.2	182.9	459.1	85	97.9	78	92.2	288.8	5.3	34.9	15.6	29.2	12.7	28	38.8	18.4	9	21.5	47.5	33.6	58.7	33.5	15.1	44.3	107.6	88.4	111.9	405.9	112	12.3
1001506878	Azvudine group	2903.8	1276.6	1627.2	348.6	12	191	157.6	5.2	185.7	35	35.4	87.1	3.3	0	1.5	0.5	53.2	23.2	61.9	47.4	1.6	19.4	14.1	20.6	14.8	25.8	10.9	5.3	21.8	23.4	43.3	203	88.8	13.5
1001506921	Paxlovid group	4214.1	1817	2397.1	41.4	1	1.5	39.9	0.5	1	0	36.1	3.8	0	0	0.1	0.4	0	0.3	0.6	0	0	0	0	4.6	31.5	0	0.4	1.1	0.3	2	6.1	31.5	3.7	0.1
1001507231	Paxlovid group	3111.3	1540	1571.3	235.8	7.6	0	235.8	0	0	13.9	9.7	212.2	0	0	0	0	0	0	0	0	0	13.2	0.7	3.6	6.1	36.7	10.1	37.4	84.2	43.7	10.8	142	80.4	2.7
1001507695	Paxlovid group	4061.5	2025.3	2036.2	615.5	15.2	96.4	519.1	0.6	95.8	95.4	123.3	300.5	0	0	0	0.6	15.8	0.1	7.7	72.1	0.7	85.3	9.3	57.9	65.4	62.1	0.7	51.5	94.2	92	14.5	423.1	174.9	2.9
1001507700	Azvudine group	4543.8	2110.1	2433.7	828.2	18.2	184.4	643.7	24.5	160	280.3	59.6	303.9	12	2.4	0.6	9.5	55.3	34.7	40.8	29.2	52.9	177.3	50	25.5	34.1	116.2	10	29.3	79.2	69.1	117.5	484.9	200.4	25.3

1001507762	Paxlovid group	4233.6	1991.2	2242.4	317.9	7.5	173.7	144.1	71.2	102.6	17.3	5.9	120.9	22.1	13.8	17.7	17.6	6.8	3.2	42.8	49.8	11.6	2	3.7	0.2	5.7	48.7	1.9	22.7	22.6	25.1	9.9	237	70.9	0
1001507770	Paxlovid group	4929.8	2161.6	2768.2	1221	24.8	518.2	702.8	198.7	319.6	236.2	30.8	435.9	79.3	19.9	41.9	57.6	87.7	123.5	66.4	42	15.1	162.5	58.5	19.4	11.4	136.6	20.2	107.1	109.7	62.2	156.2	887.1	173.3	4.4
1001507852	Paxlovid group	3810.7	1914.2	1896.5	563.6	14.8	34	529.6	0.2	33.9	310.5	47.8	171.3	0	0.1	0	0.1	4.9	3.3	0.6	25.1	86.4	167.7	56.4	44.2	3.7	63.5	3.3	15.4	38.9	50.2	142.5	210.5	148.7	61.8
1001508045	Azvudine group	3154.6	1376.8	1777.8	615.8	19.5	221.5	394.2	80.2	141.3	105.5	67.2	221.5	22.2	13.8	32.1	12	41.9	27.3	32.7	39.4	7	75.8	22.7	44.3	22.9	78.1	4	72.8	52	14.6	105.5	378.1	107.5	24.5
1001508128	Paxlovid group	3006.5	1475.6	1530.9	2087.1	69.4	920.8	1166.3	267.5	653.2	507.6	221.9	436.8	70.4	98.7	41.6	56.8	104.3	142.7	166.1	240.1	203.7	139.2	164.6	112.7	109.2	95.5	41.1	104.7	87	108.6	84.7	813.3	964.9	224.2
1001508196	Azvudine group	4109.6	1746.4	2363.1	463.2	11.3	327.9	135.3	77.2	250.8	27.7	28.4	79.2	6.9	26.9	13.8	29.6	12.7	49.5	83.1	105.5	8.1	9.9	9.7	14	14.4	24.2	0.6	0.1	14.6	39.8	76.5	303.6	72.9	10.2
1001508315	Paxlovid group	4904.5	2226.6	2678	2862.1	58.4	1479.7	1382.4	652.8	826.9	492.3	239.7	650.4	227.4	196.9	126.2	102.3	206.9	141.4	207	271.7	104.1	246.2	142.1	117.7	122	195.7	39.6	97.2	168.8	149	1360.2	1342.9	99.4	59.6
1001508345	Paxlovid group	2653	1023.5	1629.5	1017.3	38.3	297.2	720.1	231.6	65.6	340.8	110.1	269.3	103.8	33.1	60.4	34.2	23.5	27.5	8.5	6.1	81.4	140.8	118.5	96.9	13.2	65.1	7	90.5	49.3	57.2	126.5	610.1	254.8	26
1001508362	Paxlovid group	3694.6	1944.1	1750.6	1171.6	31.7	459.9	711.7	201.7	258.2	240.3	150.9	320.5	50.6	37.9	53	60.3	47.6	52.4	68.7	89.4	47.7	107.6	85	96.2	54.6	81.9	14.3	67.8	77.1	79.3	97	619.7	376.7	78.1
1001508422	Paxlovid group	3585.1	1314.2	2270.9	709.6	19.8	65.3	644.3	45.2	20.1	248.4	97.2	298.6	16.4	5.7	9.4	13.7	2.7	1	1.6	14.8	93.6	89.1	65.7	71.1	26.1	54.7	9	109.6	85.1	40.3	300.7	246.4	125.4	37.2
1001508428	Paxlovid group	5797.2	2872.5	2924.7	0	0	0	0	0	0	0	0	0	0	0	0	0	0	0	0	0	0	0	0	0	0	0	0	0	0	0	0	0	0	0
1001508558	Azvudine group	2706.8	947.6	1759.2	430.9	15.9	193	237.9	12.9	180.1	21.8	17.7	198.4	3	0	0	9.9	48.4	41.2	48.6	42	9.5	9.1	3.1	17.3	0.4	65	9.8	23.4	57.2	43	51.1	252.3	117	10.5
1001508562	Paxlovid group	3427.3	1567.4	1859.9	1307.6	38.2	577.2	730.4	347	230.2	396.9	84.8	248.7	70.9	148.9	68.4	58.8	44.7	86.2	61.3	38.1	42.6	162.1	192.2	53.7	31	85	16.6	81.7	36	29.4	188.2	980.6	131.4	7.4
1001508634	Paxlovid group	2845.1	1270.6	1574.4	1174.6	41.3	626.1	548.4	287.3	338.8	236.9	37.5	274.1	83.7	110.1	32.8	60.6	55.6	126.5	95.4	61.3	71.9	98	67	20	17.4	66.8	22	50	66.7	68.6	98.9	587.4	427.7	60.5
1001508703	Paxlovid group	3080.7	1396.4	1684.3	1072.6	34.8	433.3	639.4	93	340.2	191.2	77.2	371	3.9	24.1	14.5	50.6	41	94.7	116.1	88.4	61.5	46	83.7	40	37.2	82.9	38.6	89.7	102.4	57.5	83.3	637.5	316.5	35.3
1001508736	Paxlovid group	5272.2	2464.8	2807.4	1453.2	27.6	669.2	784.1	190.8	478.4	199.6	75.4	509	27.5	41.8	48.5	72.9	63.4	174.9	102.5	137.6	8.7	101.6	89.3	30.2	45.2	113.3	22.9	89.8	150.4	132.6	476	891.8	77.3	8.2
1001508782	Paxlovid group	4849.3	2122.7	2726.5	806.9	16.6	321.6	485.4	264.4	57.2	300.3	51.6	133.5	150.1	84	26.9	3.3	28.4	0	8.4	20.3	110.6	138.6	51.1	31.4	20.2	70.1	1.5	11.1	44.4	6.4	163.1	427.2	192.1	24.6
1001508807	Paxlovid group	4195.8	1869.5	2326.2	1320.2	31.5	649.9	670.3	321	328.9	263.8	36	370.5	108.7	48.2	92.3	71.7	62.7	88.6	88.6	89	62	160.6	41.3	20.1	15.9	94.2	28.9	41.5	122.4	83.5	154	1065.1	96.4	4.7
1001508812	Paxlovid group	3559.6	1604.9	1954.6	488.1	13.7	294.6	193.5	206.9	87.6	41.7	12.5	139.4	106.1	98.9	0.3	1.7	32.9	4	21.5	29.2	18.4	12	11.3	5.6	6.8	20.5	3	34.5	40.6	40.8	111.2	296.7	68.6	11.6
1001508812	Paxlovid group	3559.6	1604.9	1954.6	488.1	13.7	294.6	193.5	206.9	87.6	41.7	12.5	139.4	106.1	98.9	0.3	1.7	32.9	4	21.5	29.2	18.4	12	11.3	5.6	6.8	20.5	3	34.5	40.6	40.8	111.2	296.7	68.6	11.6
1001508913	Paxlovid group	3888.8	1710.4	2178.3	173.9	4.5	128	45.9	68.4	59.7	7.8	6.7	31.4	0	25.6	30.7	12.1	0.8	17.8	32	9.1	2.5	3.2	2.1	3.7	3	9.8	1.4	1	7.6	11.6	14.5	140.8	18.4	0.3
1001508964	Paxlovid group	2633.2	1291.9	1341.3	612.5	23.3	286	326.5	153	133	96.1	57.6	172.9	51.8	18.9	44.2	38.1	26.1	43.2	36.9	26.7	5.5	55.6	35	35	22.5	45.1	2.6	39	37.7	48.5	38.2	456.4	117.6	0.3
1001508980	Paxlovid group	6940.3	3450.3	3490	7.8	0.1	7.2	0.6	0	7.2	0	0.6	0	0	0	0	0	1.1	0	4.5	1.6	0	0	0	0.6	0	0	0	0	0	0	1.5	5.4	0.9	0
1001509079	Paxlovid group	2492.7	1333.9	1158.8	933.3	37.4	517.6	415.7	261.7	255.9	230.5	29.2	156.1	60.2	86.1	56.4	59	69.1	82.4	71.8	32.6	58.4	83.4	88.8	16.8	12.4	49.1	14.5	35.4	22	35	65.4	435.8	365.3	66.8
1001509081	Paxlovid group	4517.5	2267	2250.4	656.8	14.5	102.4	554.4	14.4	88	105.5	110.5	338.4	0	7.3	1.6	5.5	23.6	19.6	23.3	21.5	3	47	55.5	70.3	40.2	66.3	10	67.1	108	87	71.7	532.6	51.3	1.2
1001509084	Paxlovid group	3171.7	1309.7	1862	999.2	31.5	352	647.3	156.1	195.9	192.3	69.3	385.7	104	25.6	14.1	12.4	29.5	34.2	78.8	53.4	60.9	117.2	14.2	45.2	24.1	104.9	20.4	110.7	86.9	62.7	34.7	652.5	298.8	13.2
1001509110	Paxlovid group	2882.1	1423.1	1459	1714.2	59.5	665.5	1048.7	341.1	324.4	616	86.2	346.5	117.5	87.2	66.6	69.8	35.3	117.6	105.4	66.1	171.2	219.4	225.4	27	59.2	69.4	41.2	65.9	87	83.1	27.4	1194.8	478.1	13.9
1001509124	Paxlovid group	4611.7	2112.6	2499.1	831.2	18	373.8	457.4	131	242.8	132.8	42.9	281.7	54.4	7	22.5	47.1	59.4	39.1	111.7	32.7	4.8	82.7	45.3	21.5	21.4	121.4	10.8	19.9	68.4	61.1	46.7	489.7	271.4	23.5
1001509140	Azvudine group	3202.1	1230.6	1971.5	121.5	3.8	90.3	31.2	44.7	45.6	6.2	12.8	12.2	2.2	3.8	14.1	24.6	3.5	13.9	12.8	15.4	0.4	5.8	0	1.5	11.3	0.6	0.7	2.8	3	5.1	5.4	93.3	21.9	1
1001509151	Paxlovid group	4145	1968.6	2176.4	135.7	3.3	44.1	91.6	8	36.1	38.1	13.9	39.6	2.4	2.1	1.9	1.6	5	0.4	11	19.7	5.2	3.5	29.4	12.3	1.5	5	0.4	2.7	20.5	11	7.6	108.7	19	0.4
1001509181	Paxlovid group	2314.1	1066.5	1247.6	211.9	9.2	74.1	137.8	8.8	65.3	5.7	21.7	110.4	0.6	2.8	0.4	5	1.4	19.1	8.8	36	0.5	0.7	4.6	8.5	13.1	18.5	9	24.8	24.6	33.4	2.1	163.2	46.5	0.1
1001509183	Paxlovid group	3654.9	1324.5	2330.4	991.5	27.1	376.3	615.3	128.8	247.4	139.6	28.4	447.2	19.6	38	53.9	17.3	28.5	98.6	79.6	40.7	27.5	54.2	57.9	8.8	19.6	79.3	43.7	110.8	91.5	121.8	46.2	735.1	189.6	20.7
1001509188	Paxlovid group	3635.7	1757.5	1878.1	1629.6	44.8	711.6	918	305.2	406.4	321.5	87	509.5	81.4	77.6	92.8	53.4	77.4	110.2	111.5	107.3	128.3	107.2	86	59.8	27.3	121.7	18	136.1	113.8	119.9	51.7	935.8	641.6	0.6
1001509218	Paxlovid group	5000.7	2560.7	2440	919.6	18.4	257.9	661.8	32.5	225.3	252.7	80.8	328.3	6.1	12.5	6.9	7	76	33.3	49.8	66.3	28.8	173.2	50.6	65.3	15.5	89.5	18.7	88.7	84.7	46.7	189.7	627	86.3	16.7
1001509233	Paxlovid group	2477.7	1028.7	1449	207.1	8.4	21.5	185.7	7.6	13.9	57.3	19.9	108.5	5.8	1.8	0	0	1.8	3.6	3.9	4.5	15.7	17.3	24.3	16	3.9	42	1.7	9.8	47	7.9	3.6	177.5	26	0
1001509251	Paxlovid group	3522.6	1593.3	1929.4	506.5	14.4	212.2	294.3	98	114.2	51.4	59	184	31.3	20.5	21.7	24.5	32.1	32.7	12.1	37.3	10.9	16.8	23.7	44.3	14.7	47.6	3.4	48.5	36.6	48	33.2	420	53.1	0.1
1001509259	Paxlovid group	2542.8	1034.7	1508.2	1801.8	70.9	614.2	1187.6	428.5	185.7	575.7	332.9	279.1	84.2	25.7	106.1	212.5	1.6	62.1	39.8	82.1	221.6	160.4	193.7	142.2	190.7	80.6	25.9	67.3	67.2	38.1	7.6	988.8	794.5	10.9
1001509272	Paxlovid group	2649.4	1266.9	1382.5	756.8	28.6	266.6	490.3	108.1	158.4	141	42.4	306.9	0	21	47.1	40	9.4	98.1	41.4	9.5	33	71.7	36.2	16.4	26	65.4	12.4	61.4	124	43.7	35.5	244.9	361.7	114.8
1001509335	Paxlovid group	3107.3	1522.7	1584.6	1240	39.9	471.4	768.6	206.7	264.7	290.5	97.2	380.9	52.5	46.4	47.1	60.8	49	57.2	78.2	80.3	56.1	128.8	105.6	55.3	41.9	85	34.4	118.6	70.6	72.3	93.5	896.2	250	0.3
1001509354	Paxlovid group	4580.6	2231.9	2348.7	448.5	9.8	141	307.5	22	119	4.1	129.9	173.5	0	0.7	2.4	18.9	7	78.9	28.4	4.7	0.4	0.1	3.6	54.6	75.4	5.2	16.1	13.6	60.2	78.4	75.8	290.3	77.8	4.6
1001509385	Paxlovid group	2634.1	1261.8	1372.2	285.3	10.8	38.1	247.2	21.2	16.8	131.6	12.5	103.1	6.6	3.9	6.9	3.8	0.9	4.9	8.4	2.6	56.9	60.9	13.8	6.6	5.9	42.5	1.2	24.8	27.5	7.1	44.1	177.8	57.1	6.3
1001509393	Paxlovid group	4418.4	2099.3	2319.1	1457.2	33	779.7	677.4	303.1	476.6	242.9	188.9	245.7	79.8	45.2	95.5	82.6	41.2	116.9	97.4	221.2	66.8	107.2	68.8	127.7	61.1	60.4	14	27.5	94.6	49.3	654.8	697.5	77.1	27.8
1001509401	Paxlovid group	4305.7	1869.5	2436.2	1502	34.9	649.9	852.1	244.4	405.5	202	189.7	460.5	68.1	49.7	49	77.7	53.8	137.5	120.2	94	24.6	93.4	84	113.5	76.2	128.9	31.8	108.5	83.8	107.4	27.4	784.8	688.7	1.1
1001509405	Paxlovid group	3525.3	1759.4	1765.9	1823.4	51.7	755.4	1068.1	360.5	394.9	304	228.1	536	71.4	95.3	88.8	105	54.4	155.1	86.5	98.8	66.1	114.9	123	113.3	114.8	95.2	46.2	144.5	104.7	145.4	187.4	1133.8	461.3	41
1001509462	Paxlovid group	3959	1614.3	2344.7	1550.6	39.2	814.1	736.5	386.9	427.2	300.9	69.6	366	151.2	23.5	95.5	116.7	104.9	52.6	116.3	153.5	34.4	174.1	92.4	54.1	15.6	134.6	25.3	58.8	80.6	66.6	85.6	562.2	715.3	187.5
1001509481	Paxlovid group	2769.5	1303	1466.5	889.5	32.1	333.6	555.9	159.5	174.1	223.2	51.8	280.9	48	59.4	31.8	20.3	37.4	32.2	49.8	54.7	54.2	96	73	33.3	18.5	74.9	1.4	54	87.7	62.9	86	505.4	273.9	24.2
1001509512	Paxlovid group	4507.2	2095.8	2411.3	962.5	21.4	450.9	511.6	48.9	401.9	182.6	38.6	290.5	15.1	1.1	4.1	28.6	109.7	128.5	84.1	79.6	28.8	144.5	9.3	20	18.5	105.8	6	53.6	61.8	63.2	246	505.3	172.5	38.7
1001509526	Paxlovid group	3587.3	1549.6	2037.7	1837.3	51.2	718.5	1118.7	368.3	350.2	491.7	179.2	447.8	86.8	131.5	100.5	49.5	49.4	90.3	122.1	88.5	150.5	122.7	218.5	134.9	44.3	109.5	37.8	84.2	103.8	112.4	125.3	1157.2	553.8	0.9
1001509539	Paxlovid group	4905.5	2262.6	2642.9	8.6	0.2	4	4.6	0.1	4	0.8	1.4	2.5	0.1	0	0	0	0	0	0	4	0	0.3	0.5	0	1.4	0	2.2	0	0.2	0	1.7	5.6	1	0.3
1001509575	Paxlovid group	2490.8	1105.4	1385.4	1757.4	70.6	736	1021.4	350	386	405.8	161.3	454.2	120.2	71.5	88.1	70.2	40	112	155	78.9	126.4	93.9	185.5	87.7	73.7	124.7	43.6	102.2	113.9	69.8	151.4	838.9	626.9	140.2
1001509596	Paxlovid group	2226.6	921.2	1305.4	901.2	40.5	414.4	486.8	226.8	187.6	148.2	92.6	246	71.3	47.2	71.5	36.7	57.3	53.8	38.6	37.9	10.3	102.9	35	44.6	48	68.4	15.1	70.9	42.8	48.7	19.2	676.5	205.2	0.2
1001509630	Paxlovid group	2675.5	1567.4	1108.1	1291.1	48.3	419.3	871.8	132	287.4	416.9	78.1	376.8	23.3	12.6	39.5	56.5	5.2	62.6	105.5	114	198.1	127.2	91.6	32	46.1	61.7	74.6	102.4	77	61.1	4.9	404.8	876.2	5.3
1001509635	Paxlovid group	5581.2	2645.5	2935.7	1808.4	32.4	647.7	1160.7	245	402.7	492.1	109.5	559	123.9	1.1	41.1	79	71.5	178.1	81.9	71.1	126.8	228.1	137.2	94.2	15.3	129.2	27.8	129.4	136.3	136.3	244.8	1516.6	45.1	1.9
1001509636	Paxlovid group	2475.2	1107.4	1367.8	1031	41.7	561.6	469.4	209.4	352.2	254	37.7	177.7	83.1	42	43.1	41.3	50	85.5	116.8	100	132.4	116	5.5	25.2	12.5	52.6	15.9	32.3	49.6	27.4	35.9	586.9	406	2.2
1001509637	Azvudine group	2724.2	1240.9	1483.4	494.9	18.2	226.8	268	66.8	160	63.8	28.9	175.4	23.9	35.7	1.1	6.1	46.1	29.8	24.6	59.5	0.2	10.7	52.8	25.5	3.4	42.2	23.6	22.1	54.4	33.1	68.2	197.9	158	70.7
1001509666	Paxlovid group	2251.7	1014.5	1237.1	1095.3	48.6	610	485.2	333.7	276.4	274.4	55.9	155	140.4	58	67.6	67.7	52.8	72.1	87.8	63.6	55.7	145.8	72.8	33.2	22.7	45.3	12.3	56.6	16.2	24.6	172.5	580.7	270.3	71.7
1001509673	Paxlovid group	3640.9	1610.8	2030.1	479.4	13.2	196.3	283.1	61	135.3	55.2	51.2	176.7	14.2	17.6	21.6	7.6	5.8	33.4	54.4	41.7	5.4	22.9	26.8	29.6	21.7	51.8	5.9	38.7	46.2	34.1	101.9	248	105.9	23.7
1001509716	Paxlovid group	2862.4	1246.7	1615.7	630.9	22	130.7	500.2	96.3	34.4	184.5	26.9	288.7	9.5	0.3	30	56.5	11.4	16.5	1.7	4.7	78.6	105.5	0.4	17.2	9.7	112.2	13.6	27.6	91.4	44	106.2	343.6	142.4	38.7
1001509764	Paxlovid group	3539.2	1555.7	1983.5	2126.5	60.1	871.5	1254.9	335.5	536.1	508.5	125.4	621	150.6	46.1	64.3	74.5	108	93.3	203.7	131.1	216.4	175	117.1	44.4	81	189.6	9.9	110.8	157.8	153	279.8	1075.6	636.9	134.1

1001509795	Paxlovid group	3755.3	1634	2121.3	0	0	0	0	0	0	0	0	0	0	0	0	0	0	0	0	0	0	0	0	0	0	0	0	0	0	0	0	0	0	0
1001509810	Paxlovid group	3057.7	1266.7	1791	788.6	25.8	431.3	357.3	220.7	210.6	68.7	35.6	253	88.1	19.1	49.3	64.2	45.4	74.3	57.7	33.2	1.2	67.5	0	16.2	19.4	90.2	14.3	51.7	53.5	43.2	32.6	572.7	182.9	0.3
1001509863	Paxlovid group	4545.2	2028.4	2516.8	365.9	8	167.9	197.9	12.4	155.5	39.2	1.3	157.5	1.3	0.7	1.2	9.2	0.1	9	79.5	67	7.3	28.6	3.3	0.6	0.7	1.1	8.3	26.6	59.4	62	79.9	164.6	89.7	31.7
1001509880	Paxlovid group	4521.9	2035.1	2486.8	25.5	0.6	24.5	1	12.1	12.4	0	0	1	0.7	9.3	2.1	0	6.8	2.8	1.8	0.9	0	0	0	0	0	1	0	0	0	0	5.7	14.1	5.1	0.6
1001509957	Azvudine group	2292.4	793.5	1498.8	219.4	9.6	102.9	116.5	33.4	69.5	27.2	5.1	84.2	23.8	0	1	8.5	12.4	18.9	33	5.1	8.3	18.3	0.7	4	1	22.1	5	4.5	30.5	22.1	36.8	87.7	73.8	21.2
1001509972	Paxlovid group	2966.8	1613.9	1352.9	659.3	22.2	92.8	566.5	53.6	39.1	137.9	35.7	392.9	7.1	18.1	2.9	25.5	5.1	8.4	11.1	14.5	13	119	5.8	32.5	3.2	82.4	59.5	47.8	81.7	121.6	38.3	405.7	195.9	19.4
1001510021	Paxlovid group	3369.1	1226.3	2142.8	70.4	2.1	37.2	33.2	3.9	33.2	10.4	3.2	19.6	3.9	0	0	0	10.7	1.9	6.7	14	2.6	1.8	6.1	1.3	1.9	4.3	0.9	0	0.4	14	10.4	30.3	21.3	8.4
1001510043	Paxlovid group	3634.5	1520.6	2113.9	357.4	9.8	355.7	1.7	234	121.7	0.6	0.8	0.3	102.7	22	64.8	44.5	10.2	39.8	52.1	19.5	0.1	0	0.6	0	0.8	0	0	0	0.3	0	16.2	213	123.3	4.9
1001510077	Azvudine group	3811.8	1913.4	1898.4	703.4	18.5	235.9	467.5	71.5	164.4	139.9	72.7	254.9	25.8	4	23.9	17.7	33.8	8.6	40.8	81.1	16.7	89.6	33.5	50.1	22.6	98.4	6.2	37	58.3	54.9	107.6	310.8	216.7	68.3
1001510114	Azvudine group	3784.9	1754.4	2030.5	815.1	21.5	249.4	565.7	164.8	84.6	270.2	111.1	184.3	37.3	32.3	67.2	28	30.7	9.1	31.1	13.7	48.9	96.7	124.6	65.6	45.5	25.6	9.4	75.9	55	18.4	247.8	445.2	101.9	20.1
1001510119	Paxlovid group	2876	1255	1621	864.1	30	367.9	496.2	169.8	198.1	91.7	103.5	300.9	35.6	46.2	49.9	38.1	24.9	57.1	53.7	62.4	2.9	54.4	34.4	88.7	14.8	69.2	17.7	42.6	96.9	74.6	31.3	560.2	264.2	8.4
1001510136	Paxlovid group	2733.3	1280.1	1453.2	189.5	6.9	91.2	98.3	12.5	78.6	10.7	1.4	86.1	8.1	0	0.5	4	12.5	28.4	16.9	20.9	0.2	1	9.6	0.5	1	13.1	2.6	13.3	17.4	39.8	6.3	158.4	24.7	0.1
1001510205	Azvudine group	3253.3	1469.7	1783.6	0	0	0	0	0	0	0	0	0	0	0	0	0	0	0	0	0	0	0	0	0	0	0	0	0	0	0	0	0	0	0
1001510313	Paxlovid group	2189.5	931.8	1257.7	910.3	41.6	456.2	454.1	175	281.2	99.1	84.2	270.7	60.4	29.9	36	48.7	66.2	49.6	89	76.4	20.3	6.9	72	52.2	32	79.1	10	73.7	73.1	34.8	21.7	298.4	468	122.1
1001510320	Azvudine group	3141.1	1784	1357	711.5	22.7	260	451.5	158.3	101.8	203.1	12.6	235.8	61	79	16.2	2.1	35	11.3	26.8	28.6	62.2	98.7	42.2	2.7	9.9	67.6	5.8	27.4	69.4	65.6	132.8	414.4	136.6	27.7
1001510322	Paxlovid group	2574.2	1188.9	1385.3	435.8	16.9	178.9	256.9	23	155.9	2.5	33.3	221	2.7	1.4	5.4	13.6	22.7	28.6	51.2	53.3	0.2	0.7	1.7	21.1	12.3	53.3	14.7	52	53.4	47.5	34.3	165.4	184.1	52
1001510366	Paxlovid group	4714.6	2415.9	2298.7	59.5	1.3	8.6	51	0	8.6	0.4	0	50.6	0	0	0	0	0	1.3	3.5	3.7	0	0	0.4	0	0	0.9	2.8	8.2	32.1	6.5	4.7	53	1.9	0
1001510375	Paxlovid group	3396.6	1456.7	1939.8	4.6	0.1	1.5	3.2	0.5	1	0	0	3.2	0.2	0	0	0.2	0.2	0.6	0	0.2	0	0	0	0	0	1.7	0	0.9	0.3	0.3	0.1	3.5	1	0
1001510399	Paxlovid group	3132.6	1689.4	1443.1	935.6	29.9	351.4	584.3	83.3	268.1	199.8	70.5	314	3.8	13.1	29	37.4	31.5	102.8	86.8	47	35	57.2	107.6	52.8	17.7	55.8	50.9	55	80.9	71.4	83.9	621.8	207.2	22.8
1001510496	Paxlovid group	2389.3	1289.1	1100.2	1280.5	53.6	546.2	734.3	176.9	369.3	258.8	78	397.4	47.3	71.6	21.6	36.4	46.6	122.6	98	102.1	96	88	74.7	39.2	38.8	105.1	53.3	48.1	83.4	107.5	80.2	687.7	480.8	31.7
1001510543	Paxlovid group	2503.4	1180.8	1322.6	326.6	13	39.7	286.8	25.6	14.2	18.5	17.6	250.7	6	0.7	6.3	12.5	8.2	5.9	0.1	0	0	1.4	17.1	8.1	9.5	63.5	8	45.6	66.6	67	44.5	190.9	78.4	12.7
1001510579	Paxlovid group	3187	1287	1900	1318.9	41.4	564.4	754.5	233.8	330.5	160.5	121.7	472.4	83.2	25.8	69	55.9	69.7	70.7	102.5	87.6	41.4	63.4	55.7	52.7	69	120.8	42.7	105.7	117.9	85.3	134.9	693.1	414.2	76.7
1001510638	Paxlovid group	2240	1024	1216	177.9	7.9	77.1	100.8	17.1	59.9	9.5	3.1	88.1	0.1	4.5	10	2.5	1.8	9.7	36.3	12.2	0	2.9	6.6	0.1	3	19.9	1.1	15.6	25.3	26.2	6.6	116	52.4	2.8
1001510649	Paxlovid group	2782	1025.2	1756.8	836.1	30.1	513	323.1	247.1	265.8	71.2	4.8	247.1	87	30.2	58.9	71	31.9	80.5	95.7	57.8	9.5	60.8	0.9	2.6	2.2	63.4	7.3	33.8	75.9	66.7	43	357.3	363.2	72.6
1001510652	Paxlovid group	4497.9	2039.6	2458.4	804	17.9	453.5	350.5	114.7	338.8	35.4	22.4	292.7	14.4	31.3	24.9	44.2	27.3	63.4	110.1	138	2.1	18.7	14.6	6.2	16.2	65.2	6.3	31.1	97.5	92.6	68.5	441.9	259.7	33.8
1001510678	Paxlovid group	2494.8	1054.5	1440.3	882.2	35.4	413.4	468.8	166.6	246.8	68.6	75.5	324.7	44.2	30.1	29.4	62.8	28.1	90.9	88.1	39.8	3.4	56.6	8.6	37.4	38.2	36.2	48.8	78.1	109.9	51.7	31.6	543.6	294.1	13
1001510694	Paxlovid group	3304.8	1561	1743.8	916.1	27.7	459.3	456.9	203.8	255.4	103.9	35.9	317	61.7	20.4	68.5	53.2	55.3	63.9	85	51.2	1.9	83.9	18.1	13.3	22.6	110.7	24.6	26.9	84.4	70.3	55.6	377.3	413	70.2
1001510853	Paxlovid group	2053.6	784.7	1269	892.4	43.5	189.2	703.2	106.7	82.5	278.9	100.7	323.5	27.3	57.3	14.8	7.3	11.9	41.6	12.7	16.2	49.1	83.1	146.6	73.4	27.3	93.2	35.3	58.6	64.2	72.3	66.1	431.2	341.6	53.5
1001510893	Paxlovid group	2287.9	1084.5	1203.4	173.5	7.6	21.6	151.9	6.1	15.5	67.8	12.3	71.8	0.9	2.5	2.7	0	6.3	4.6	2	2.7	16.7	16.7	34.4	8.8	3.5	12.7	2.9	34.7	16.8	4.8	21.1	95	46.7	10.8
1001510908	Paxlovid group	5140.7	2263.3	2877.4	634.9	12.3	312	322.9	39.4	272.6	3.8	16.6	302.5	0.6	24.1	3.3	11.4	13.1	52.2	105.6	101.8	0.1	1.4	2.3	6.5	10	11.1	3	32.3	109.6	146.5	159.6	369.7	88.9	16.7
1001510924	Paxlovid group	3380.2	1552.5	1827.7	1804.7	53.4	684.5	1120.1	301.1	383.4	406.9	233.3	480	40.5	62.9	119.3	78.4	57.5	100.4	134.6	90.9	85.2	124.6	197.1	136.6	96.7	126.5	31.9	138.5	91	92.1	185.6	1036.3	489.4	93.4
1001510952	Paxlovid group	3708	1659.6	2048.4	2093	56.4	977.3	1115.7	463	514.3	370.2	172.8	572.7	184.8	48.3	107.7	122.3	58	151	178.5	126.8	124	179	67.2	116.8	56	131.9	38.1	172.1	120.7	109.8	115.3	1064.4	826.1	87.2
1001510963	Paxlovid group	1806.7	760.6	1046	468.5	25.9	227.5	241	40	187.5	58.1	92.3	90.6	18.3	4.7	3.6	13.3	55.8	40.1	38.2	53.4	7.4	28.3	22.3	32.5	59.8	35.7	14.6	15	16.2	9	9.1	266	179	14.3
1001510967	Paxlovid group	3718.7	1762	1956.7	1488.6	40	534.1	954.5	322.5	211.6	493	53.9	407.6	99.8	123.7	46.1	52.9	33.6	15.6	20.3	142.2	98.4	223.8	170.8	34.8	19	158.9	12.9	107	91.9	36.9	96.3	651	612.2	129.1
1001510981	Azvudine group	2431.2	1235.4	1195.8	354.3	14.6	209.1	145.2	87	122.2	66.4	47.5	31.2	3.8	29.2	38.3	15.7	36	24.5	32.1	29.6	1.4	29.1	35.9	44	3.5	14.2	0	2.9	0.7	13.4	88.9	183.1	65.6	16.7
1001511035	Paxlovid group	3386.3	1722.8	1663.5	1791.4	52.9	861.1	930.3	493.7	367.4	394.7	144.7	390.9	226.8	173.3	63.3	30.4	96	70.1	118.5	82.8	123.6	130.6	140.4	118.2	26.5	115.9	20.5	52.8	113.9	87.8	397.8	1044.1	296.1	53.5
1001511036	Azvudine group	1724.3	754.8	969.5	391.3	22.7	135.4	255.9	55.2	80.2	135	40.4	80.5	36.5	9.8	3.7	5.3	11.9	16.4	27.1	24.8	39.8	47.5	47.8	7.9	32.5	18.4	28.6	15	10.5	7.9	64.7	169.5	108.5	48.6
1001511064	Paxlovid group	6565.8	3224.5	3341.3	8.4	0.1	4.5	4	0.5	4	0	1.6	2.3	0	0	0	0.5	0.1	0	3.2	0.6	0	0	0	0	1.6	1.8	0	0	0.4	0.2	3.1	4.6	0.6	0.1
1001511172	Azvudine group	3256.1	1588.7	1667.4	166.8	5.1	29.9	137	2.3	27.5	59	0.1	77.8	0	0.3	0	2	0	6.9	14.7	6	6.2	46.2	6.6	0	0.1	30.2	2.4	25.3	13.2	6.7	9.1	75.5	73.4	8.8
1001511225	Paxlovid group	2313.4	1127.8	1185.5	239.3	10.3	141.3	98	2.9	138.4	24.8	1.5	71.7	2.3	0	0.5	0.1	20.8	24.1	47.2	46.4	0	24.8	0	0.4	1.1	22.5	4.4	7	22	15.9	11.8	123	90.6	13.9
1001511272	Paxlovid group	2719.8	1195.3	1524.5	283.1	10.4	103.6	179.5	35.7	67.8	40.6	4.1	134.7	0.4	21.4	13.5	0.5	1.8	10.8	26.9	28.4	1.6	5.1	33.9	3.1	1	15.7	10.8	27.6	43.3	37.3	8.1	194.4	78.6	2.1
1001511317	Paxlovid group	3952.3	1680.5	2271.8	38.6	1	21	17.6	9.7	11.2	9	1.7	6.9	0	0	0.5	9.3	0	6.1	4.7	0.4	5.3	3	0.7	0.3	1.4	5.7	0	0.7	0.4	0	3.2	32.8	2.6	0
1001511341	Paxlovid group	3474.7	1382.8	2091.8	1047.3	30.1	420.9	626.3	154.9	266	253.3	74.6	298.4	37.6	29.5	45.5	42.2	52.1	58.1	66.2	89.7	47.3	110.3	95.8	47.9	26.8	105	16.2	47.7	73.4	56.1	169.1	582.7	263.1	32.4
1001511385	Paxlovid group	2236.3	1103.2	1133.1	234.7	10.5	71.1	163.6	10.6	60.5	47.5	6.6	109.5	2	0.7	1.5	6.4	1.1	22.6	15.3	21.5	10.7	30.1	6.8	4.5	2.1	22.4	6.4	33.8	14	33	16.7	146.1	65.1	6.9
1001511388	Paxlovid group	3264.6	1460	1804.7	62.7	1.9	2.2	60.5	0.7	1.5	0.2	0	60.3	0	0.3	0.4	0	0	0	0	1.5	0	0.2	0	0	0	30.6	7.6	0	11.6	10.5	15.9	38.9	7.3	0.6
1001511407	Paxlovid group	2992.3	1573.2	1419.1	1481.8	49.5	594.4	887.4	263.7	330.7	309.1	160.4	417.8	85.7	70.2	81.7	26.1	108.6	85.8	57.6	78.7	91.3	121.7	96.1	77.7	82.7	83.7	43.1	73	125.6	92.4	170.2	857.2	415.3	39
1001511416	Paxlovid group	1867.1	799.8	1067.3	855.3	45.8	260.8	594.6	155.3	105.5	260.9	54.1	279.6	22	54.3	48.1	30.8	24.2	44.6	33.8	3	34.5	75.9	150.4	30.4	23.7	76.3	19.1	55.3	82	46.9	26.7	355.3	392.7	80.6
1001511446	Paxlovid group	3808.5	1778.6	2029.9	1496.3	39.3	659	837.2	372.3	286.7	469.6	60.6	307	133.4	88.3	89.2	61.5	49.6	52.4	106.4	78.3	129.8	133.4	206.4	36.7	23.9	73.3	0	84.3	87	62.3	213	1198.8	81.2	3.3
1001511494	Azvudine group	3268.9	1545	1723.8	1328.6	40.6	575.1	753.5	257.6	317.5	268.9	94.6	390.1	94.2	50.3	60.5	52.6	48.9	51.8	126.1	90.7	50.5	129.3	89	66.5	28.2	103	18.9	73.2	106.5	88.5	72.9	578.2	561.1	116.4
1001511518	Azvudine group	1960.1	732	1228	529.8	27	217.3	312.5	104.7	112.7	37	99.8	175.8	38.1	9.2	32.3	25.1	22.6	32.7	36.1	21.4	7.2	17.8	12	69.1	30.7	31.5	14.7	54.9	41.8	33	44.2	300.3	166	19.4
1001511520	Paxlovid group	3931.3	1684.4	2246.9	241.9	6.2	134.2	107.7	2.8	131.4	13.8	23.2	70.8	0.5	1.4	0	1	36.1	4.4	63.6	27.2	1	4.6	8.2	15.3	7.9	1.9	1.3	7.4	4.9	55.2	23.4	178.2	38.4	2
1001511533	Paxlovid group	5096.4	2240.5	2855.9	494.8	9.7	149.2	345.6	42.7	106.5	119.1	12.1	214.5	16.1	2.7	16.3	7.5	28.4	11.3	36.6	30.3	7.2	94	17.9	9.8	2.3	86.4	1.3	22.3	69.9	34.5	59.1	358.5	75.2	2.1
1001511546	Azvudine group	3397	1390.6	2006.4	360	10.6	234.8	125.2	6.3	228.5	5.7	0	119.5	1.6	0	3.5	1.2	47.5	85	61.2	34.9	0	5.7	0	0	0	38	0.6	0	20.9	60	8.3	209	134.5	8.2
1001511550	Paxlovid group	3057.2	1267.1	1790.1	143.6	4.7	21.4	122.1	13	8.4	20.1	16.4	85.7	0.6	0.8	6.8	4.8	0	7	0.1	1.3	0.9	11.7	7.5	9.4	6.9	23.5	12.7	9.8	14	25.7	4.8	113.6	23.8	1.3
1001511571	Paxlovid group	2714.2	1190.3	1523.9	594.8	21.9	288.9	305.9	115.6	173.2	64.9	55	186	20.8	37.6	37.6	19.7	36.6	53.7	51.1	31.9	0	24.5	40.4	29.5	25.5	9.4	27.5	38.7	45.8	64.5	86.7	311.7	146.5	49.9
1001511576	Paxlovid group	2372.3	974.1	1398.2	1027.1	43.3	437	590.1	183	254.1	131.9	120.2	338	48.8	24.1	37.4	72.7	13.3	70.8	78.4	91.7	24.7	65.4	41.8	64	56.1	88.1	54.2	47.4	84.7	63.6	40.2	465	452.4	69.4
1001511599	Azvudine group	3247.5	1359	1888.5	1006.4	31	366.7	639.7	69	297.7	211.5	77.2	351.1	23.7	18.9	12.9	13.4	51.5	50.7	98.8	96.7	41.5	99.9	70.1	51.6	25.6	82.7	17.3	90.3	85.3	75.4	28.7	564.5	412.6	0.6
1001511622	Paxlovid group	3402.5	1516.4	1886.1	490.4	14.4	79.2	411.1	25.1	54.2	141	55	215.2	10.9	0.4	5.1	8.7	16.4	13.4	9.6	14.9	2.4	81.2	57.4	33.2	21.8	42.8	13.3	63.8	57.6	37.7	96.9	318.8	68.5	6.1
1001511627	Paxlovid group	1763.3	830.3	933	725.8	41.2	186.1	539.7	185.1	0.9	239	16.6	284.2	53.8	34.6	81.8	14.9	0	0.9	0	0	28.4	71.7	138.9	9.7	6.9	8.5	118.5	87.6	36.6	32.9	37.2	537.2	150.9	0.5

1001511678	Azvudine group	2735.1	1310.3	1424.8	252.3	9.2	142.5	109.8	42.6	99.9	16.2	17	76.5	1.4	13.7	17.7	9.8	1	41.7	30.7	26.4	4.3	3.5	8.5	3.1	13.9	10.3	13.3	25.2	0.6	27	5.2	175.6	71.3	0.2
1001511757	Paxlovid group	2441.7	1242.9	1198.8	854.8	35	325.9	528.9	140.9	185	130.8	86.9	311.2	12.9	60	38.4	29.6	14.5	61.5	51.6	57.3	12.9	19.8	98.1	48.2	38.7	68.3	20.6	57.7	97.2	67.4	42.2	565.5	238.4	8.6
1001511842	Paxlovid group	4050.5	1751.6	2298.9	799.7	19.7	256.3	543.5	5.7	250.6	88.9	34.4	420.1	0	0	1.2	4.4	14.4	82.1	77.6	76.5	6.4	52.7	29.9	23.2	11.3	109.9	5.9	70.6	134.8	98.9	24.6	551.7	223.2	0.1
1001511860	Paxlovid group	2446.1	1158.2	1287.8	314	12.8	27.4	286.6	1.9	25.5	86.9	5.7	194	0.9	0.1	0	0.9	1.8	0	5.2	18.5	5.3	66.1	15.5	2.2	3.5	72.1	2.4	42.6	48.7	28	21.7	174.4	102.7	15.3
1001511878	Azvudine group	4720.9	2125.8	2595	289.8	6.1	38.3	251.5	2.7	35.6	49.5	0.2	201.9	0	0	0	2.7	13.5	6.9	6.2	9	0.1	49.2	0.2	0.2	0	69.1	1.8	9.2	76.6	45.2	35.5	203.7	46.4	4.2
1001511898	Azvudine group	1377.9	577.6	800.3	805.4	58.5	334.6	470.8	182	152.6	183.8	71.1	215.9	78.5	29.6	41.4	32.4	42	37.1	48.1	25.4	42.1	64.3	77.4	31.5	39.6	58.2	26.2	24.1	73.8	33.7	41.8	303.3	354.7	105.6
1001511921	Azvudine group	2845.3	1351.8	1493.4	996.2	35	348.1	648.1	188.1	160	301.5	93.3	253.2	89	54.1	30.8	14.2	25.1	43.4	53.2	38.3	58.8	164.6	78.1	71.3	22.1	61.2	7.5	62	60.7	61.9	53.2	637	289.2	16.8
1001511922	Paxlovid group	2729.5	1465.4	1264.1	390.2	14.3	61.8	328.4	21.7	40.1	128.4	31.3	168.7	1.8	6.8	6.1	7	0.9	11.3	22.5	5.4	21	68.8	38.5	18.1	13.2	39.5	8.4	20.9	63.7	36.1	108.7	234.8	43.5	3.1
1001511932	Paxlovid group	4194.9	1936.1	2258.8	298.9	7.1	189.6	109.3	89.3	100.3	6.9	15.2	87.1	58.8	11.4	6.6	12.5	24.7	24.2	35.5	15.8	0	5.9	1	15.2	0	14.7	2	14.8	31.8	23.8	82.9	152.6	50.8	12.6
1001511962	Azvudine group	2245.5	1303.1	942.4	122.2	5.4	58.2	64	16.5	41.6	0	0	64	4.9	5.3	0.2	6.2	0.2	6.5	10.8	24.1	0	0	0	0	0	0	6.4	15.4	29.3	13	4.8	74.4	37.8	5.2
1001511963	Azvudine group	2588.1	1187.1	1401.1	524.1	20.3	298.9	225.2	137.4	161.5	77	9.7	138.5	39.1	22.7	45.7	30	24.8	57.2	56	23.5	8.5	46	22.6	0.8	8.9	29.5	5.6	15.2	37.5	50.7	2.7	379.3	141.2	0.9
1001512009	Paxlovid group	2269.9	990.3	1279.6	287.9	12.7	92.3	195.6	75.6	16.7	26.1	8.1	161.4	19.8	4.1	28.3	23.4	9.1	6.1	1.2	0.4	0.6	4.6	20.9	3.3	4.9	41.1	9.4	3.9	29.4	77.6	31.7	171.2	72.5	12.5
1001512075	Paxlovid group	2312.3	1030	1282.3	27.4	1.2	6.5	20.9	0	6.5	0	3	17.8	0	0	0	0	0.2	1.5	3.8	1.1	0	0	0	3	0	2.2	0	1.4	6.6	7.4	1.4	10	10.9	5
1001512170	Paxlovid group	3481.8	1819.3	1662.5	0	0	0	0	0	0	0	0	0	0	0	0	0	0	0	0	0	0	0	0	0	0	0	0	0	0	0	0	0	0	0
1001512297	Paxlovid group	2849.8	1262.1	1587.7	400.4	14.1	272.5	127.9	81	191.5	6	66.3	55.7	41.2	11	17.1	11.8	51.3	48.2	57.4	34.5	0.3	5.2	0.4	29.6	36.6	18	2.3	2.7	14.2	18.5	32.8	232.7	121	13.9
1001512298	Paxlovid group	3141.6	1198.4	1943.2	183.9	5.9	121.3	62.7	25.6	95.6	3.9	20.5	38.3	1.9	16.2	5.6	2	3.6	30.8	34.6	26.6	0.8	0	3	4.4	16.1	12.4	2.6	10.7	5.5	7.2	12.3	105.4	59.7	6.5
1001512329	Paxlovid group	3080.3	1224	1856.3	1019.3	33.1	479	540.3	180	299	112.4	58.4	369.5	61.1	72	24.9	22	30.3	93.4	124.4	50.8	25.5	66	20.9	46	12.4	60	25.3	146.8	79.4	58	93.3	675.4	240.3	10.4
1001512357	Paxlovid group	3299.5	1647.1	1652.4	47.6	1.4	37.3	10.3	1.7	35.5	7.8	2	0.6	0	0	0	1.7	4.6	5	1	24.9	0	7.1	0.7	0	2	0	0.1	0	0	0.5	4.2	35.9	7.1	0.4
1001512437	Paxlovid group	2301.3	1117.9	1183.4	282.3	12.3	15.9	266.4	1.5	14.4	95.6	7.5	163.3	0.3	0.1	0.9	0.2	1.6	2.8	2.2	7.9	15.5	57.4	22.7	6.2	1.3	42.7	12	4.9	61.3	42.4	46	182.2	48.3	5.8
1001512445	Paxlovid group	2916.5	1423.2	1493.3	801.3	27.5	378	423.2	87.7	290.3	116.9	41.1	265.2	8.6	14.8	40.8	23.5	41.2	69.4	103.4	76.2	24.3	17.9	74.7	35.2	6	37.5	16.5	35.4	100.9	74.9	8	617.7	175	0.5
1001512477	Paxlovid group	5876.6	2596.6	3280	248.8	4.2	45	203.8	0.8	44.1	4.3	12.7	186.9	0	0	0	0.8	0.7	4.4	12.1	26.9	0.1	4.2	0	4.6	8.2	23.4	6.3	109.3	37.2	10.7	47.9	178.5	22.4	0
1001512510	Azvudine group	4407.1	2128	2279.1	1263.8	28.7	404.3	859.4	281.9	122.5	455.9	255.1	148.4	138.2	80.6	44.8	18.3	13	49.3	36.2	24.1	106.6	140.9	208.4	122.8	132.3	18.3	49	57.7	15.7	7.7	238.5	928.1	89.3	7.9
1001512530	Azvudine group	2766.7	1230.6	1536.1	1466.4	53	623.8	842.6	231.6	392.2	288.3	132.7	421.5	68.6	65.3	43.4	54.3	64.1	100	146.9	81.2	73.2	100	115.1	88.8	43.9	103.4	44.3	76.7	106	91.1	64.7	673.3	651.9	76.6
1001512579	Azvudine group	3809.5	2040.6	1768.9	882.3	23.2	171	711.3	89.9	81	207.3	138.5	365.5	15.6	5.5	31.7	37.2	12	15	27	26.9	68.3	96.8	42.2	119.8	18.7	129.3	24.3	69.9	71	71	132.4	410.8	258.6	80.6
1001512593	Azvudine group	3954.6	1790.8	2163.7	1736.4	43.9	494.3	1242.1	100.2	394	414.6	144.2	683.4	17.5	1.8	22.2	58.7	64.4	135.4	94.3	99.8	97.8	148.8	168	107.3	36.8	167	55.6	157.3	186	117.4	175.8	954.2	519.7	86.6
1001512713	Azvudine group	3744.7	1980.7	1763.9	814.4	21.7	267.8	546.7	82.2	185.6	431.6	105.7	9.3	67.8	0.2	7.5	6.7	64.9	45.6	70	5.1	137.7	74.1	219.8	90.7	15	8.7	0.3	0.3	0	0	111.5	352.8	274	76
1001512788	Paxlovid group	6120.6	2663.7	3456.9	169.6	2.8	15.5	154.1	13	2.5	79.1	0.3	74.7	5.4	2.7	3.8	1	0.5	0.6	1.4	0.1	7.1	71.5	0.6	0.2	0	37.3	0.1	1.2	11.7	24.4	69.8	80.8	15	4.1
1001512810	Paxlovid group	3856.3	2201.7	1654.6	868.8	22.5	426.3	442.5	136.3	290	90.9	0.6	350.9	16.1	14.3	22.4	83.4	36.8	123.3	82.7	47.2	4.5	78.2	8.2	0.4	0.3	121.5	18.1	8.3	60.3	142.8	100.6	446.1	259.6	62.6
1001512817	Paxlovid group	3362.4	1487.5	1874.9	71.2	2.1	35.4	35.8	7.1	28.3	1.6	0	34.1	0	0	3.3	3.8	0	7.1	7.6	13.7	0.8	0	0.8	0	0	0	2.5	8.5	8.9	14.2	1.2	64.5	5.5	0
1001512962	Paxlovid group	2499	1125.8	1373.2	871	34.9	439.1	431.8	117.3	321.9	123.1	34.2	274.5	29.4	11.3	44.2	32.3	52.5	105.2	78.6	85.5	2.7	59.9	60.5	20.1	14.2	69.2	10.6	34.4	82.9	77.3	95.1	594.6	171.6	9.7
1001512970	Paxlovid group	3665.7	1604.1	2061.6	329.6	9	153.1	176.5	78	75.1	0	0.8	175.7	25.5	15.8	11.5	25.2	17.1	26.7	20.4	11	0	0	0	0	0.8	20.5	9.2	40.9	41.9	63.3	61.5	167.4	76.6	24.1
1001512976	Azvudine group	4673.1	2042.4	2630.7	5.4	0.1	2.1	3.3	0	2.1	0.1	0	3.2	0	0	0	0	0	0	1	1.1	0	0	0.1	0	0	0.5	2.7	0	0	0	1.2	3.6	0.6	0
1001513547	Azvudine group	3755.3	1634	2121.3	0	0	0	0	0	0	0	0	0	0	0	0	0	0	0	0	0	0	0	0	0	0	0	0	0	0	0	0	0	0	0
1001513565	Azvudine group	2150.7	891.7	1259	446.7	20.8	206.2	240.5	90.2	116	30.6	53.8	156.1	9.8	25.9	27.2	27.3	32.2	32	26.6	25.2	7.9	17.7	5	19.9	33.9	41.7	1.9	39.6	52.5	20.5	20.1	281.9	144.3	0.4
1001513682	Paxlovid group	3315.7	1598.1	1717.7	105.5	3.2	28.6	76.9	1.8	26.7	2.5	5.6	68.9	0	1.8	0	0	0	9.7	14.2	2.8	0.2	1.8	0.5	0	5.6	2.4	0.8	17	19.9	28.8	4.1	90.3	11.1	0
1001513769	Paxlovid group	2465	1099.7	1365.3	1498.4	60.8	598.6	899.8	349.3	249.3	387.5	83	429.2	144.3	84.7	61.7	58.5	64.9	51.5	54.9	78.1	107.6	158.1	121.8	31.1	52	91.7	42.5	91.9	114	89.2	110.2	728.9	548.3	111
1001513829	Azvudine group	2959	1359.5	1599.5	125.3	4.2	38.5	86.8	13.6	24.9	27.8	1.9	57.1	5.1	0	0	8.5	0.4	3.5	11.6	9.4	0.2	24.8	2.8	1.1	0.8	4	0.1	5	26.2	21.8	13.7	72.4	33.8	5.5
1001513912	Azvudine group	3107.2	1391.8	1715.4	451.7	14.5	126.3	325.5	61.8	64.5	134.4	18.2	172.9	26.4	2.8	11.2	21.4	9.6	18.4	22.1	14.3	6.6	74.9	53	13.1	5	59.5	0	44.1	58.5	10.8	11.1	336.6	102.8	1.2
1001513938	Paxlovid group	3363.1	1416.5	1946.6	301.3	9	67.5	233.9	4.8	62.6	69	7.1	157.7	0.3	0.5	3	1.1	0	0	19.9	42.7	7.7	2.1	59.2	4.6	2.5	15.2	0.8	53.7	58.9	29.3	30.1	243.8	26.6	0.9
1001513996	Azvudine group	4848.6	2282.9	2565.7	171.5	3.5	68.3	103.2	12.3	56	4.2	0.3	98.6	3.6	1.4	0.4	6.8	4.1	14.8	18	19.1	2.3	2	0	0.3	0	14.8	11.9	2.9	31.6	37.4	10.3	141.6	19.5	0.2
1001514127	Azvudine group	2926.2	1656.5	1269.7	454	15.5	97.2	356.8	38.6	58.6	175.8	27.7	153.3	0	0.1	11.8	26.8	5.6	15.2	22.3	15.6	12.2	73.7	89.9	13.4	14.3	44.1	5.6	49.6	30.2	23.8	64.3	270.4	100.1	19.3
1001514157	Azvudine group	2699.2	1037.8	1661.4	665.3	24.6	253.3	412	153.8	99.6	192.9	6	213.1	87.1	15.9	42.3	8.5	25.8	6.6	38.5	28.6	56.2	83.7	52.9	2	4.1	78.9	8.6	26.4	73.5	25.8	84.1	394.1	160.4	26.7
1001514167	Azvudine group	4220.9	2101	2119.9	383.2	9.1	122.1	261.1	2.3	119.8	3.2	104.1	153.8	2.2	0.1	0	0	42.4	0.2	26.4	50.9	2.7	0.3	0.2	40.8	63.3	33.7	0.4	45.1	50.7	24	20.7	171.8	170.4	20.2
1001514299	Paxlovid group	5865.4	2559.4	3306	130.6	2.2	110.4	20.2	0	110.4	0	0	20.2	0	0	0	0	0.3	11.9	50.1	48.2	0	0	0	0	0	0	5.4	0	9.2	5.7	21.6	88	19.8	1.2
1001514365	Azvudine group	3007	1359.3	1647.7	492.5	16.4	78.7	413.9	11.9	66.7	233.9	68.9	111.1	4	0.1	2.2	5.6	9.4	2.7	18.3	36.4	22.6	43.6	167.7	44.4	24.5	16.7	8.1	67	9.7	9.7	27.4	152.4	215.6	97.2
1001514509	Paxlovid group	2873	1079.2	1793.8	614.2	21.4	289.5	324.8	105.1	184.4	92	7.2	225.6	52.9	17.7	21.5	13	58.7	18.5	65.8	41.4	8.3	74.9	8.8	4.1	3.1	76.5	3.3	39.7	68.8	37.4	70.6	291	186.4	66.3
1001514559	Paxlovid group	2421.4	1139.6	1281.8	1618.8	66.9	770.3	848.5	359	411.3	367	101.8	379.7	72.8	48.1	83.8	154.4	48.5	104.9	111	147	127.5	141.2	98.4	62.2	39.6	70.3	42.7	74.8	109.8	82.2	14.4	531.5	1008.9	64
1001515002	Paxlovid group	4586.6	2199.6	2386.9	0	0	0	0	0	0	0	0	0	0	0	0	0	0	0	0	0	0	0	0	0	0	0	0	0	0	0	0	0	0	0
1001515325	Azvudine group	3335.7	1539.4	1796.3	93.8	2.8	4.9	88.9	4.3	0.6	33.5	3.6	51.9	1.3	0	0.1	2.9	0.6	0	0	0	2.7	30.3	0.5	0	3.6	39.8	0.7	5.1	4.2	2.1	12.7	73	7.6	0.5
1001515623	Paxlovid group	3360.9	1499.3	1861.7	582.4	17.3	284.4	298	104.4	180.1	5.6	6.8	285.6	19.9	46.4	22.3	15.7	5.5	38.1	62.5	73.9	2.4	1.8	1.5	1.8	5	11	13.8	57.6	61.9	141.3	13.9	474.7	93.7	0.1
1001515709	Paxlovid group	1515	690.1	824.9	36.6	2.4	9.1	27.5	3.3	5.8	6.1	0	21.4	0	0.1	2.4	0.7	0	0.9	2.2	2.7	0	0.4	5.7	0	0	1.8	2.2	0.2	4.2	13	4.1	21	9.2	2.3
1001515832	Paxlovid group	5157.9	2709.8	2448.2	296.4	5.7	39.1	257.3	10.4	28.7	110.2	35.3	111.7	6	0	4.4	0	1.9	4.5	14.1	8.2	39.4	57.1	13.7	29.9	5.4	46.5	2.6	13.9	45.6	3.2	154.5	111.6	20.6	9.7
1001515881	Paxlovid group	2866.2	1151.9	1714.3	644	22.5	415.6	228.4	79.9	335.7	3.5	10.3	214.6	65.6	2.2	4	8.2	75.1	87.1	94	79.5	0.7	1.6	1.3	4.4	6	60.5	17	21.7	69.1	46.3	100.1	326.2	156.8	60.9
1001516174	Paxlovid group	3299.3	1495.5	1803.7	1179.7	35.8	561.6	618.1	321.8	239.8	217.8	99.6	300.6	182.2	73.9	51.8	13.9	70.7	57.1	54.9	57	66.8	133.8	17.3	54.5	45.1	67.1	21	74.5	93.9	44.1	236.8	519.4	313.9	109.6
1001516201	Paxlovid group	2317.6	1386.5	931.2	36	1.6	35.2	0.9	30.6	4.6	0.2	0	0.7	2.2	0	11.5	16.9	0.4	0.1	2.1	2	0.1	0	0	0	0	0.1	0	0.6	0	0	6.3	19.4	8.7	1.7
1001516202	Paxlovid group	3795.6	1866.4	1929.2	258	6.8	44.7	213.3	16.3	28.3	22.2	1.7	189.4	0.8	2.7	10.7	2.2	7.1	0.6	5.8	14.9	0	22.1	0	0.2	1.5	48.3	3.6	13	60.1	64.4	11	183.9	61.4	1.7
1001516283	Paxlovid group	4011.6	1752.2	2259.4	161.7	4	116.6	45.1	36.5	80.1	19	2.3	23.8	22.2	0	2.3	12	5.4	14.1	40.3	20.3	0.6	16.9	1.6	0.1	2.2	1.5	1.9	0.6	9.6	10.3	65.9	73.2	16.3	6.3
1001519865	Paxlovid group	2774.7	1456.3	1318.5	84.1	3	16.7	67.4	4.7	12	0	0.8	66.6	1	2.6	0.7	0.4	0	0	10	2	0	0	0	0.4	0.4	9.5	15.7	8.6	11.6	21.2	1.8	38.2	39	5.1
1001520158	Paxlovid group	3255.2	1862.4	1392.8	1136.5	34.9	612.6	524	343.3	269.3	401.4	117.5	5.1	37.7	113.6	59.1	132.8	6.6	92.9	35.1	134.7	164.3	2.5	234.6	55.2	62.3	4	0.3	0.8	0	0	54.8	874.6	192.2	15

1001520201	Azvudine group	5236.3	2424.3	2812	375	7.2	209.2	165.8	64.9	144.3	106.4	5.4	54	32	5.8	18.8	8.4	65.6	14.9	26.7	37.1	80.6	20.3	5.6	2.6	2.8	16.3	2.7	8.4	10.3	16.3	152.7	151.8	47.6	22.8
1001520209	Azvudine group	3481.6	1392.8	2088.8	1427.7	41	485.9	941.8	153.8	332	382.9	153.5	405.4	21.1	53.3	21.8	57.6	62.1	70.3	102.1	97.6	170.5	35.8	176.6	78	75.5	41.3	69.1	115.6	130	49.3	97.3	698.7	549.8	81.9
1001523061	Paxlovid group	5065.8	2303.5	2762.3	0	0	0	0	0	0	0	0	0	0	0	0	0	0	0	0	0	0	0	0	0	0	0	0	0	0	0	0	0	0	0
1001523301	Paxlovid group	3083.8	1247.2	1836.6	2095.9	68	787.3	1308.6	427.6	359.7	539.7	201.7	567.2	176.1	102.2	100.3	48.9	62.8	101	103.5	92.5	146.3	132.5	260.9	90.7	111.1	159.8	29	145.9	132.6	99.9	432.4	1272.4	296.1	95
1001531337	Paxlovid group	2881	1372.3	1508.7	59.8	2.1	10.6	49.2	4.9	5.8	10	4.5	34.7	2.4	2	0.4	0.1	1.5	1.7	1.1	1.5	0	5.4	4.6	2.1	2.4	16.5	0.3	3.1	10.5	4.3	22.9	33	3.5	0.4
1001533418	Paxlovid group	5744.8	2556.6	3188.2	143.3	2.5	47.3	96	34.9	12.4	94.8	0.2	1	18.6	9.3	6.2	0.8	11.9	0.5	0	0	32.4	29.6	32.8	0.2	0	0.2	0.7	0	0.1	0	33	34.2	37.6	38.5
