# Supplementary material for: Antiviral Effectiveness, Clinical Outcomes, and Artificial Intelligence Imaging Analysis for Hospitalized COVID‐19 Patients Receiving Antivirals
Source: Influenza Other Respir Viruses. 2024 Sep 16;18(9):e70006. doi: 10.1111/irv.70006 (PMC11405122; doi:10.1111/irv.70006)

Supplementary Material

**Content**

Disease severity classification standards from the 'Chinese Diagnosis and Treatment Protocol for COVID-19 (Trial Version 10)'…………………………………………..2

Supplementary table S1: Characteristics of hospitalized patients receiving nirmatrelvir/ritonavir or receiving azvudine………………………………………….3

Supplementary table S2: Stratified analysis of the impact of different baseline characteristics on patient prognosis…………………………………………………..5

Supplementary table S3: Analysis of follow-up durations and number of patients triggering endpoint events in the two groups…………………………………………6

Supplementary figure S1: Visualization of artificial intelligence imaging analysis results for chest CT scans based on VB-Net (United Imaging Intelligence)……………7

Supplementary figure S2: Scatter plot of serial cycle threshold values for hospitalized patients treated with nirmatrelvir/ritonavir or azvudine………………………………12

Supplementary figure S3: KM curves for the two groups based on the time to first negative test result ...…………………………………………………………………13

Supplementary figure S4: AI-based radiological analysis in hospitalized patients treated with nirmatrelvir/ritonavir or azvudine……………………………………………….14

Supplementary figure S5: KM curves for nirmatrelvir/ritonavir and azvudine after infection ratio incorporating into covariates of propensity score matching.………….15

Supplementary figure S6: KM curves for nirmatrelvir/ritonavir and azvudine in patients with high infection ratio (>9.2) and low infection ratio (≤9.2)………………………16

Supplementary figure S7: Scatter plot of serial cycle threshold values for all patients with infection ratio high (>9.2) and infection ratio low (≤9.2)………………………17

**Disease severity classification standards from the 'Chinese Diagnosis and Treatment Protocol for COVID-19 (Trial Version 10)'**

(i) Mild.

Characterized primarily by symptoms of upper respiratory infection such as dry throat, sore throat, cough, and fever.

(ii) Moderate.

Persistent high fever (≥38.5℃) for more than three days or symptoms such as cough and shortness of breath, but with a respiratory rate (RR) < 30 breaths/min and an oxygen saturation > 93% while breathing ambient air at rest. Chest X-ray image or CT shows characteristic manifestations of COVID-19 pneumonia.

(iii) Severe.

Adults meeting any of the following criteria that cannot be explained by a cause other than COVID-19 infection:

Shortness of breath, RR ≥ 30 breaths/min;

Oxygen saturation ≤ 93% while breathing ambient air at rest;

Partial arterial oxygen pressure (PaO2) to fraction of inspired oxygen (FiO2) ratio ≤ 300 mmHg. In high-altitude areas (above 1000 meters), the PaO2/FiO2 ratio should be adjusted according to the following formula: PaO2/FiO2 × [760 / atmospheric pressure (mmHg)];

Progressive worsening of clinical symptoms, with pulmonary imaging showing significant progression of lesions (> 50%) within 24-48 hours.

(iv) Critical.

Meeting any one of the following conditions:

Respiratory failure requiring mechanical ventilation;

Shock;

Failure of other organ systems requiring ICU care.

**Table S1.** Characteristics of hospitalized patients receiving nirmatrelvir/ritonavir or receiving azvudine

|  | Nirmatrelvir/ritonavir group  (n=318) | Azvudine group  (n=121) |
| --- | --- | --- |
| Age, years | 71.2 (15.0) | 69.9 (16.6) |
| Sex |  |  |
| Female | 213 (67%) | 73 (60%) |
| male | 105 (33%) | 48 (40%) |
| Comorbidities |  |  |
| Heart diseases | 91 (29%) | 33 (27%) |
| Cerebrovascular diseases | 48 (15%) | 23 (19%) |
| diabetes | 99 (31%) | 25 (21%) |
| Hypertension | 154 (48%) | 58 (48%) |
| Gastrointestinal or liver diseases | 71 (22%) | 20 (17%) |
| Kidney diseases | 70 (22%) | 27 (22%) |
| History of cancer | 57 (18%) | 12 (10%) |
| Smoking history | 60 (19%) | 24 (20%) |
| Alcohol consumption history | 42 (13%) | 14 (12%) |
| Laboratory test* |  |  |
| Alanine aminotransaminase, IU/L | 26 (19-38) | 22 (17-35) |
| Aspartate aminotransferase, IU/L | 36 (24-53) | 29 (20-52) |
| Total bilirubin, mmol/L | 10.8 (7.6-15.2) | 12.8 (8.8-18.3) |
| Creatinine, umol/L | 69 (58-87) | 76 (62-103) |
| Blood urea nitrogen, mmol/L | 5.6 (4.4-8.4) | 6.0 (4.2-10.7) |
| White blood cell, × 10^9^/L | 6.2 (4.3-8.7) | 5.6 (4.1-7.9) |
| Hemoglobin, g/L | 123 (111-136) | 123.0 (109-135) |
| Platelets, × 10^9^/L | 176 (124-223) | 172 (127-240) |
| International Normalized Ratio | 1.1 (1.0-1.2) | 1.1 (1.0-1.2) |
| Procalcitonin, ng/mL | 0.1 (0.1-0.4) | 0.1 (0.1-0.3) |
| C-reactive protein, mg/L | 51.4 (20.6-81.5) | 36.2 (11.5-66.7) |
| Initial Ct value† | 31.4 (27.1-35.6) | 29.9 (25.4-35.5) |

Data are mean (SD), n (%), n/N (%), or median (IQR). Data are rounded to the nearest whole number or to one decimal place, as dictated by the specifics of the dataset. *Data on alanine aminotransaminase/ aspartate aminotransferase were missing for 29 (16 in nirmatrelvir/ritonavir group, 13 in azvudine group), data on creatinine and blood urea nitrogen were missing for 28 (13 in nirmatrelvir/ritonavir group, 15 in azvudine group), data on international normalized ratio were missing for 69 (40 in nirmatrelvir/ritonavir group, 29 in azvudine group), data on white blood cell/ hemoglobin/ platelets were missing for 19 (11 in nirmatrelvir/ritonavir group, 8 in azvudine group), data on procalcitonin were missing for 35 (26 in nirmatrelvir/ritonavir group, 9 in azvudine group), data on C-reactive protein were missing for 31 (19 in nirmatrelvir/ritonavir group, 12 in azvudine group) †Data on the initial Ct value were missing for 28 patients in the nirmatrelvir/ritonavir group and 5 patients in the azvudine group, Ct values exceeding 40 or negative results, are denoted by a Ct value of 40. A total of 116 patients had an initial Ct value greater than 35, 84 in nirmatrelvir/ritonavir group, 32 in azvudine group. The initial Ct value refers to the first Ct value recorded after admission, although this measurement is not necessarily obtained from tests conducted on the first day of hospitalization.

**Table S2.** Stratified analysis of the impact of different baseline characteristics on patient prognosis

| Variables | **Before PSM** | | | **After PSM** | | |
| --- | --- | --- | --- | --- | --- | --- |
|  | **HR** | **95%CI** | **P Value** | **HR** | **95%CI** | **P Value** |
| Gender |  |  |  |  |  |  |
| Male | Reference |  |  | Reference |  |  |
| Female | 0.83 | 0.50-1.38 | 0.48 | 0.48 | 0.22-1.03 | 0.06 |
| Age |  |  |  |  |  |  |
| ＜65 | Reference |  |  | Reference |  |  |
| ≥65 | 1.30 | 0.69-2.47 | 0.42 | 3.42 | 1.02-11.90 | ***** |
| Diabetes |  |  |  |  |  |  |
| No | Reference |  |  | Reference |  |  |
| Yes | 1.15 | 0.71-1.88 | 0.57 | 0.563 | 0.23-1.36 | 0.20 |
| Hypertension |  |  |  |  |  |  |
| No | Reference |  |  | Reference |  |  |
| Yes | 0.67 | 0.42-1.09 | 0.11 | 0.48 | 0.22-1.05 | 0.07 |
| Cardiovascular disease |  |  |  |  |  |  |
| No | Reference |  |  | Reference |  |  |
| Yes | 3.04 | 1.85-5.00 | ******* | 2.04 | 0.97-4.28 | 0.06 |
| Cerebrovascular disease |  |  |  |  |  |  |
| No | Reference |  |  | Reference |  |  |
| Yes | 3.83 | 2.36-6.22 | ******* | 5.47 | 2.62-11.42 | ******* |
| Liver disease |  |  |  |  |  |  |
| No | Reference |  |  | Reference |  |  |
| Yes | 1.46 | 0.89-2.40 | 0.14 | 1.15 | 0.46-2.88 | 0.76 |
| Pulmonary disease |  |  |  |  |  |  |
| No | Reference |  |  | Reference |  |  |
| Yes | 0.84 | 0.52-1.34 | 0.46 | 0.83 | 0.42-1.64 | 0.58 |
| Kidney disease |  |  |  |  |  |  |
| No | Reference |  |  | Reference |  |  |
| Yes | 2.54 | 1.61-4.00 | ******* | 2.42 | 1.10-5.28 | ***** |
| Malignancy history |  |  |  |  |  |  |
| No | Reference |  |  | Reference |  |  |
| Yes | 0.88 | 0.44-1.76 | 0.72 | 0.60 | 0.14-2.63 | 0.50 |
| Smoking |  |  |  |  |  |  |
| No | Reference |  |  | Reference |  |  |
| Yes | 1.53 | 0.85-2.75 | 0.15 | 1.84 | 0.79-4.30 | 0.16 |
| Alcohol drink |  |  |  |  |  |  |
| No | Reference |  |  | Reference |  |  |
| Yes | 1.14 | 0.56-2.30 | 0.72 | 0.22 | 0.05-0.93 | ***** |
| *: P<0.05, **: P<0.01, ***: P<0.001  Note: PSM, propensity score matching; Calculations of HR in the table are based on the composite endpoint events mentioned in the main text. | | | | | | |

**Table S3.** Analysis of follow-up durations and number of patients triggering endpoint events in the two groups

|  | Maximum duration of follow-up | Numbers of drug users | Numbers of deaths |
| --- | --- | --- | --- |
| Before-PSM nirmatrelvir/ritonavir | 86 | 268 | 71 |
| Before-PSM azvudine | 45 | 102 | 25 |
| After-PSM nirmatrelvir/ritonavir | 48 | 87 | 23 |
| After-PSM azvudine | 45 | 87 | 17 |

**Figure S1**. Visualization of artificial intelligence imaging analysis results for chest ct scans based on VB-Net (United Imaging Intelligence).


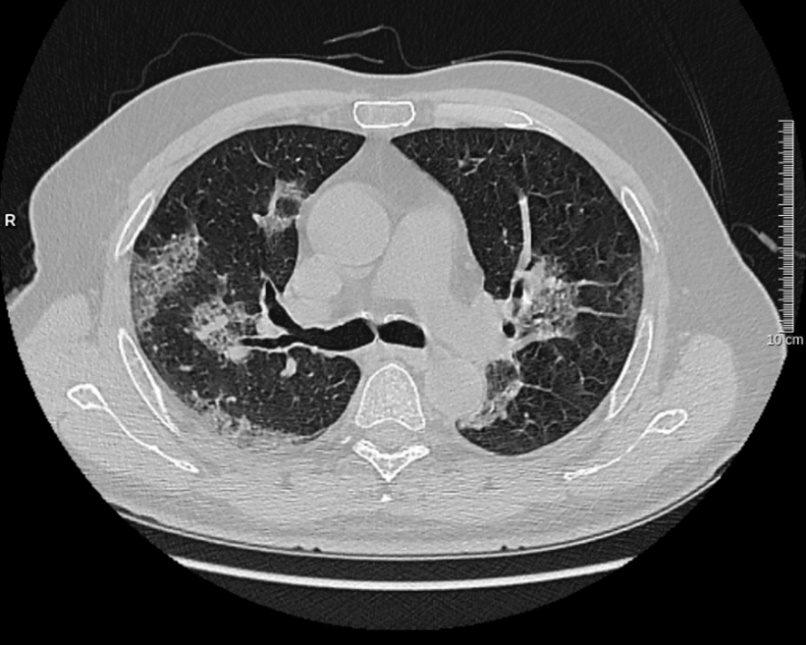


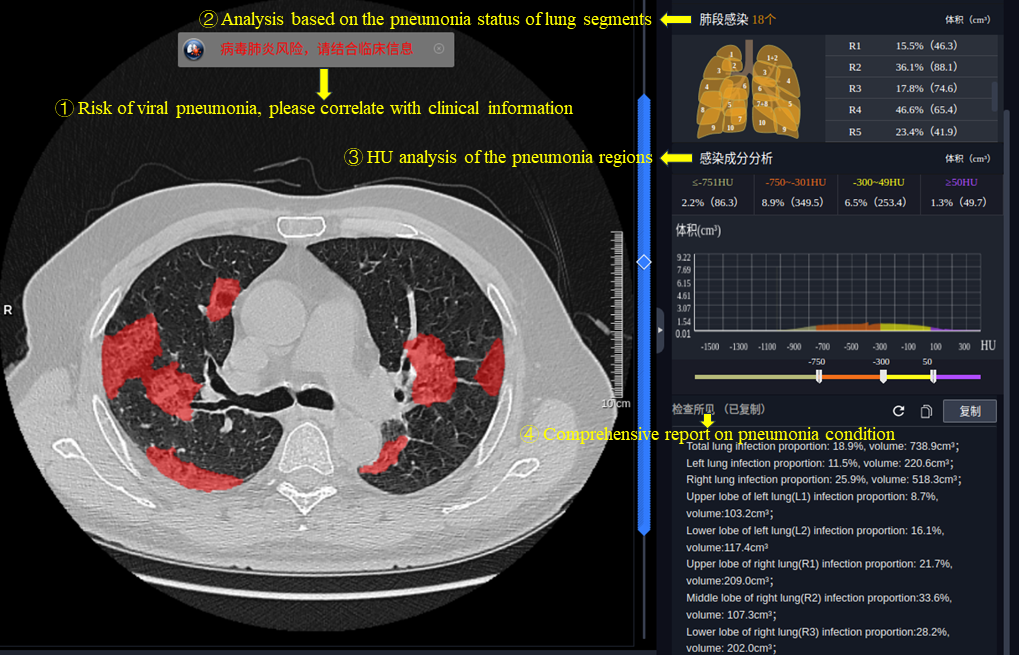


A 67-year-old male, diagnosed with COVID-19, was admitted to the hospital and treated with nirmatrelvir/ritonavir. The following describes the computation process for the pneumonia regions in the chest CT of this patient:

① The raw CT scan data were imported in "Dicom" format into an artificial intelligence software for pulmonary infection diagnostic assistance for segmentation. The software automatically divided the whole lung into lobes and segments and identified pneumonia regions. By integrating the distribution characteristics and imaging features of lesions across the whole lung, lobes, and segments, and based on an AI deep learning model, it estimated the probability of viral pneumonia, indicating the risk of viral pneumonia and suggesting a diagnosis in conjunction with clinical information. The chest CT (slice thickness of 1mm) used a red mask to mark the pneumonia infection areas on that layer.

② The three-dimensional pseudocolor rendering of lung infection indicated viral pneumonia involvement in 18 lung segments. The table on the right side, R1, showed that the apex segment of the right upper lobe had an infection ratio of 15.5% and an infection volume of 46.3 cm³. Other sections R2, R3, R4, and R5 presented the results analysis for the anterior segment of the right upper lobe, the posterior segment of the right upper lobe, the lateral segment of the right middle lobe, and the medial segment of the right middle lobe, respectively.

The volume histogram of HU values distribution in the pneumonia areas was divided into four grades based on HU values, calculating the volume and proportion of each grade relative to the whole lung. The volume of the whole lung pneumonia infection areas with CT values ≤ -751 HU was 86.3 cm³, accounting for 2.2% of the total lung volume; the infection volume within the -750 to -301 HU range was 349.5 cm³, representing 8.9% of the total lung volume; the infection volume within the -300 to 49 HU range was 253.4 cm³, accounting for 6.5% of the total lung volume; the total infection volume within the ≥50 HU range was 49.7 cm³, representing 1.3% of the total lung volume.

1. The volume histogram of HU values distribution in the pneumonia areas was divided into four grades based on HU values, calculating the volume and proportion of each grade relative to the whole lung. The volume of the whole lung pneumonia infection areas with CT values ≤ -751 HU was 86.3 cm³, accounting for 2.2% of the total lung volume; the infection volume within the -750 to -301 HU range was 349.5 cm³, representing 8.9% of the total lung volume; the infection volume within the -300 to 49 HU range was 253.4 cm³, accounting for 6.5% of the total lung volume; the total infection volume within the ≥50 HU range was 49.7 cm³, representing 1.3% of the total lung volume.
2. The result analysis report based on VB-Net calculated the total volume and infection ratio of pneumonia involvement for the entire lung and five lung lobes. The total volume ratio of pneumonia involvement in the whole lung was 18.9%, with a volume of 738.9 cm³. The left lung pneumonia involvement accounted for 11.5% of its total volume, with a volume of 220.6 cm³. The right lung pneumonia involvement accounted for 25.9% of its total volume, with a volume of 518.3 cm³. The involvement in the upper lobe of the left lung represented 8.7% of its total volume, with a volume of 103.2 cm³. The lower lobe of the left lung had a pneumonia involvement of 16.1%, with a volume of 117.4 cm³. The upper lobe of the right lung had a pneumonia involvement of 21.7%, with a volume of 209.0 cm³. The middle lobe of the right lung had a pneumonia involvement of 33.6%, with a volume of 107.3 cm³. The lower lobe of the right lung had a pneumonia involvement of 28.2%, with a volume of 202.0 cm³.

AI Model Details of VB-Net (Med Phys. 2021;48(4):1633-1645.)

Training Strategy:

The VB-Net model was trained using a human-involved-model-iterations (HIMI) strategy to assist radiologists in refining automatic annotations for each training case. This method involves iterative updating of the deep learning model by manually correcting initial segmentation results and combining them with previously annotated data. This process iteratively increases the training dataset and updates the model until the segmentation accuracy stabilizes​​.

Datasets:

The training data consisted of 249 CT scans of COVID-19 patients collected from various centers outside Shanghai. The validation was performed using 300 CT scans of COVID-19 patients from Shanghai​​.

Model Configuration:

VB-Net is a modified 3D convolutional neural network combining V-Net with a bottle-neck structure. The network includes a contracting path for global feature extraction and an expansive path for integrating fine-grained features. Specific hyper-parameters, such as the Adam optimizer with a learning rate of α=10^−4^, were used during training​​.

Accuracy Metrics:

Segmentation Accuracy:

The accuracy of VB-Net was evaluated using the Dice similarity coefficient. On the validation set of 300 CT scans, the average Dice similarity coefficient between automatic and manual segmentations was 91.6% ± 10.0%​​. The mean percentage of infection (POI) estimation error was 0.3% for the whole lung, 0.5% for lung lobes, and 0.8% for bronchopulmonary segments​​.

Comparison with Other Models:

VB-Net outperformed the popular U-Net model under the same experimental conditions. The Dice similarity coefficients for U-Net and VB-Net were compared, showing VB-Net had a higher average accuracy​​.

Reduction in Manual Annotation Time:

The HIMI strategy significantly reduced the manual annotation time from 211.3 ± 52.6 minutes to 4.7 ± 1.1 minutes after three iterations. This demonstrates the efficiency and effectiveness of integrating the HIMI strategy with deep learning models for annotating COVID-19 infection regions​​.

**Figure S2**. Scatter plot of serial cycle threshold values for all patients treated with nirmatrelvir/ritonavir and azvudine (including patients did not undergo chest CT scans).


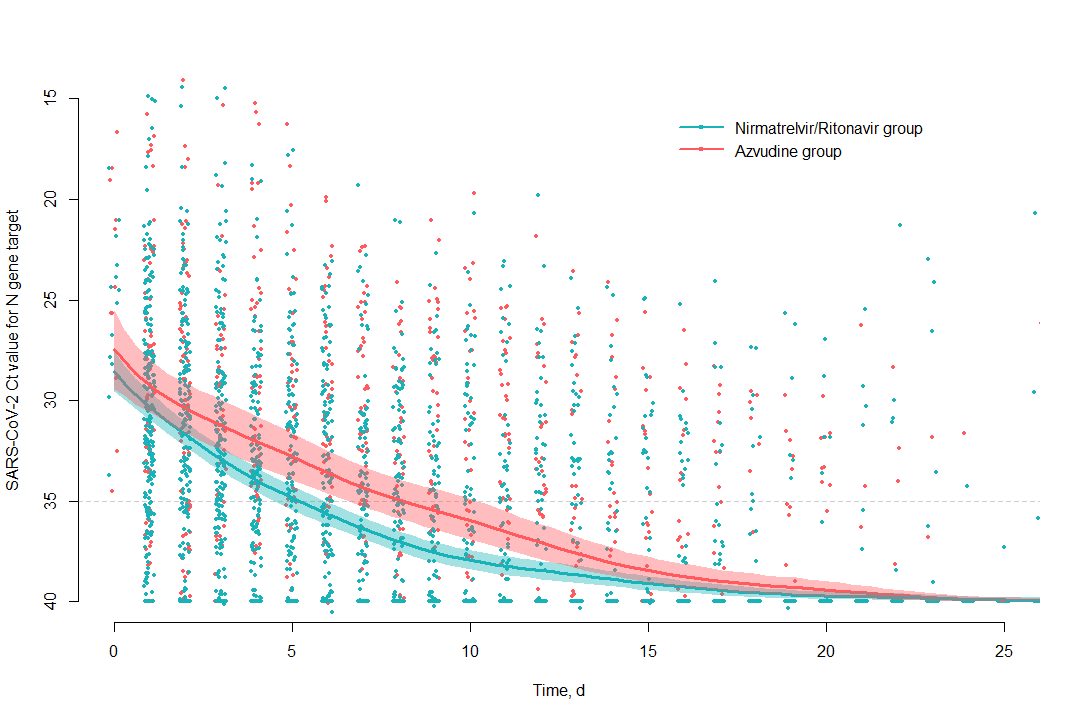


Cycle threshold (Ct) values obtained from qRT-PCR tests targeting N genes in infected individuals are depicted in the figure. blue and red circles represent patients treated with nirmatrelvir/ritonavir and azvudine, respectively. The solid lines show the average trend based on functional principal components analysis, with shaded regions illustrating 95% credible intervals for these trends. A dashed line marks the Ct threshold of 35, and any Ct values above 40 or indicating negative results are denoted by a Ct value of 40 for uniformity in representation. The blue line intersects the Ct value of 35 at 5.1 days [95% CI 4.5-6], the red line intersects the Ct value of 35 at 8.4 days [95% CI 6.6-10.2]. The nirmatrelvir/ritonavir group had a total of 1660 Ct values, the azvudine group had 783 Ct values.

**Figure S3**. KM curves for the two groups based on the time to first negative test result.


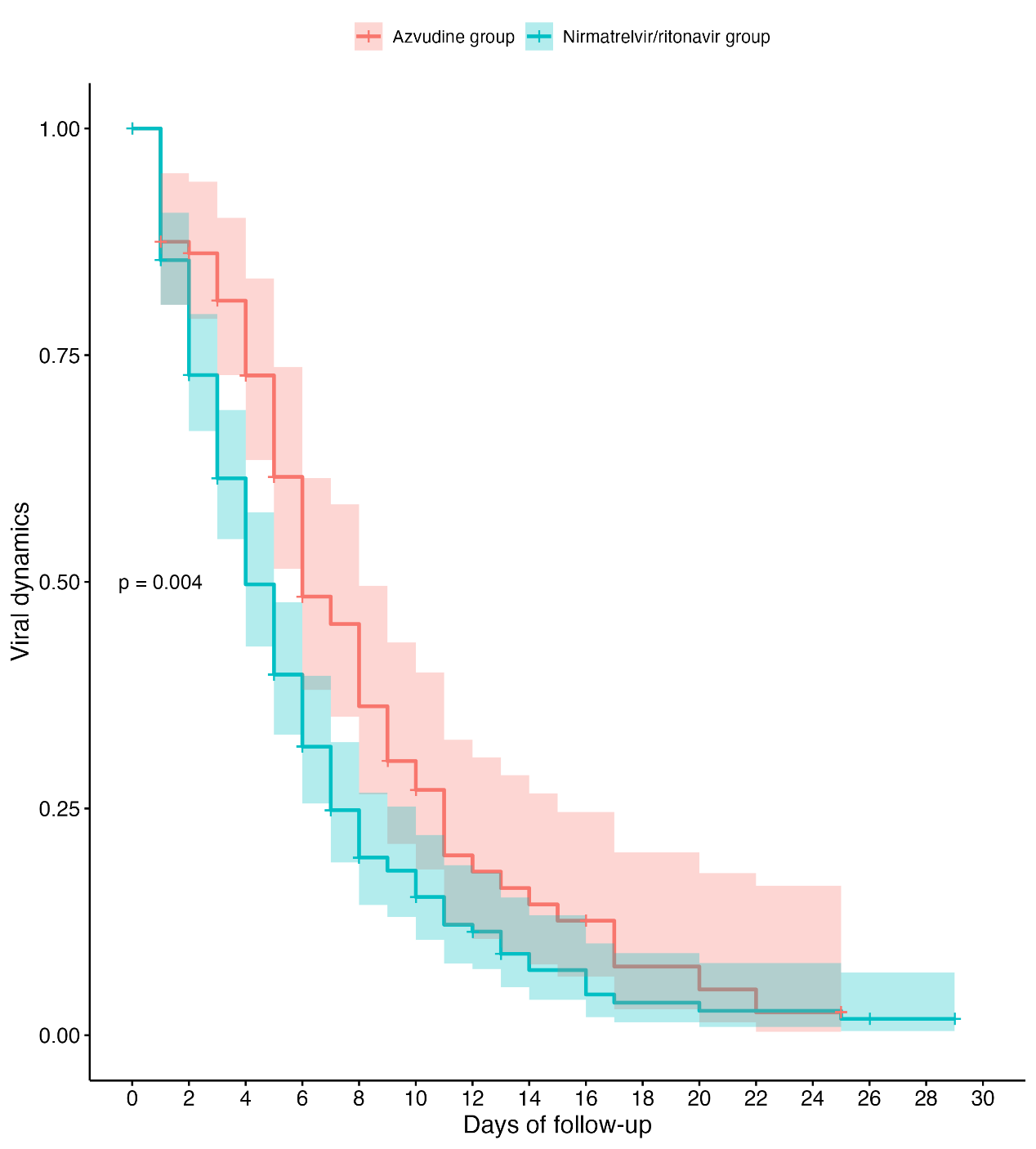


**Figure S4**. AI-based radiological analysis in hospitalized patients treated with nirmatrelvir/ritonavir or azvudine after propensity score matching.


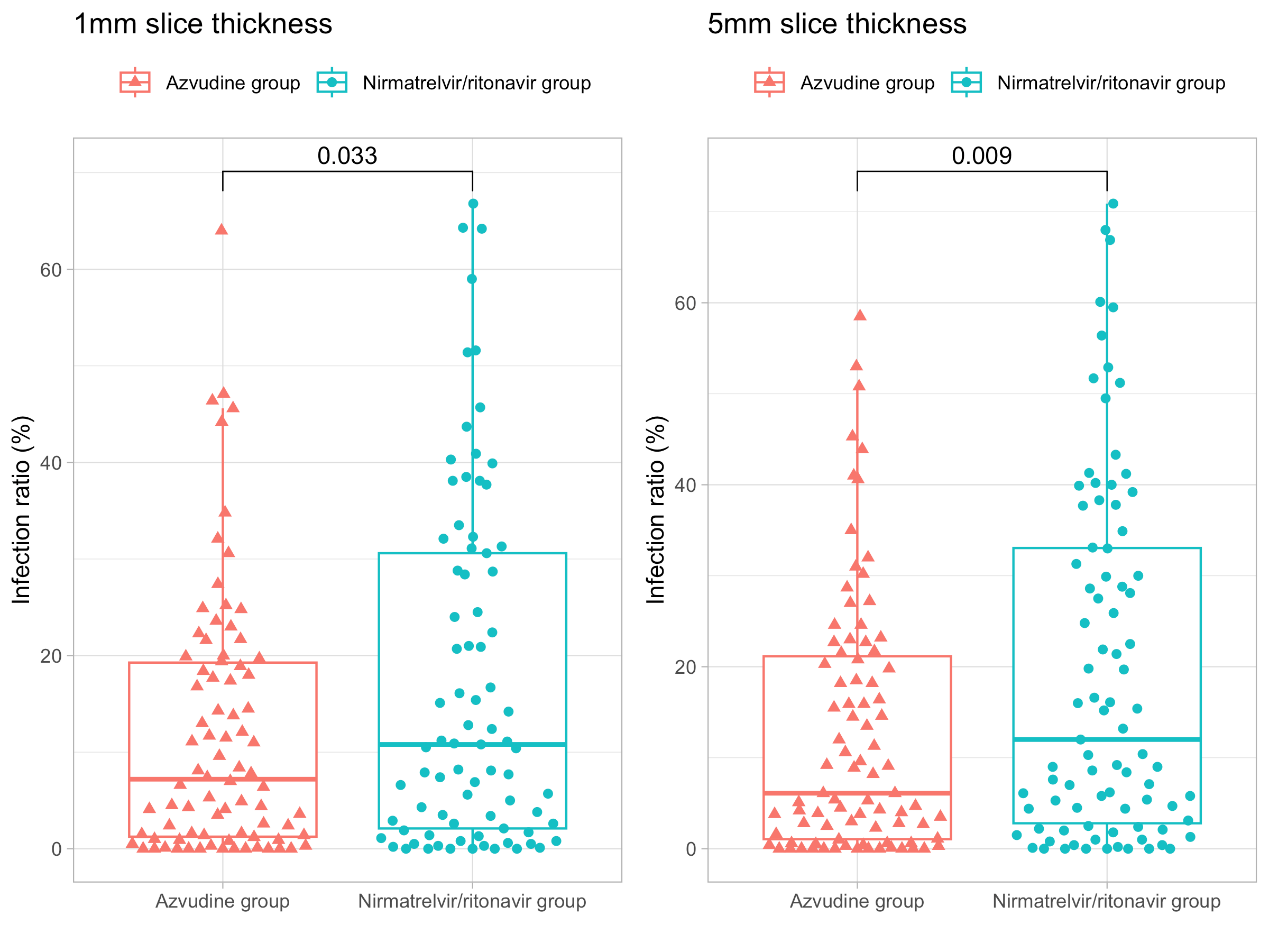


**Figure S5.** KM curves for nirmatrelvir/ritonavir and azvudine after infection ratio incorporate into covariates of propensity score matching.


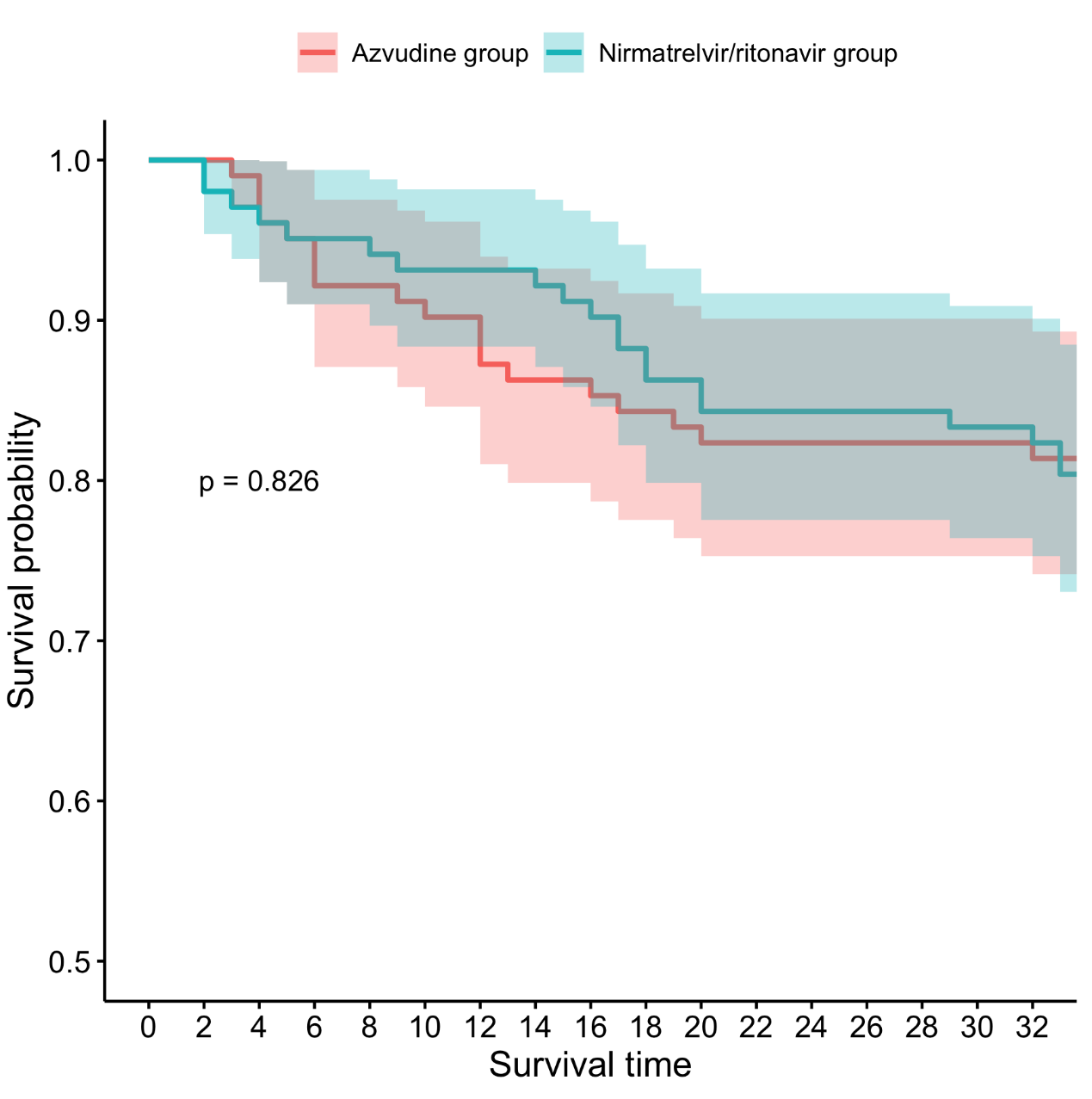


**Figure S6.** KM curves for nirmatrelvir/ritonavir and azvudine in patients with infection ratio high (>9.2) and infection ratio low (≤9.2).


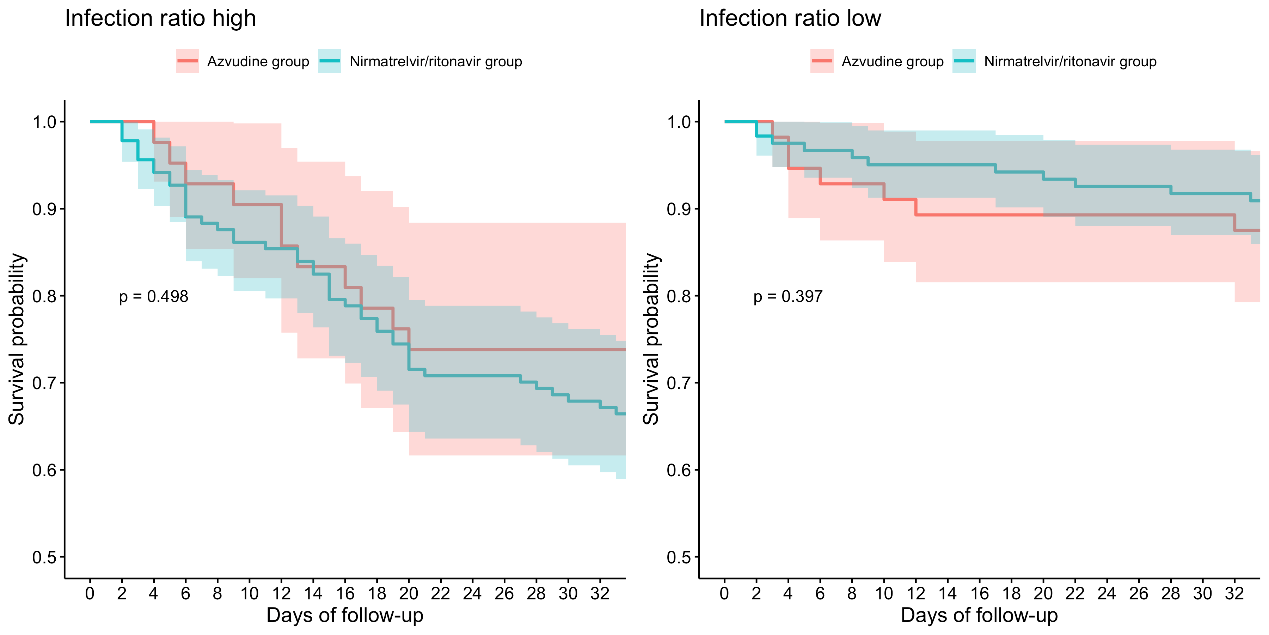


**Figure S7.** Scatter plot of serial cycle threshold values for all patients with infection ratio high (>9.2) and infection ratio low (≤9.2). As shown in the figure, although a higher proportion of patients with an infection ratio above the median were treated with nirmatrelvir/ritonavir (N/R), the change in their Ct value curve was flatter compared to patients with an infection ratio below the median. This suggests that patients with an infection ratio above the median are more likely to experience a viral clearance impairment.
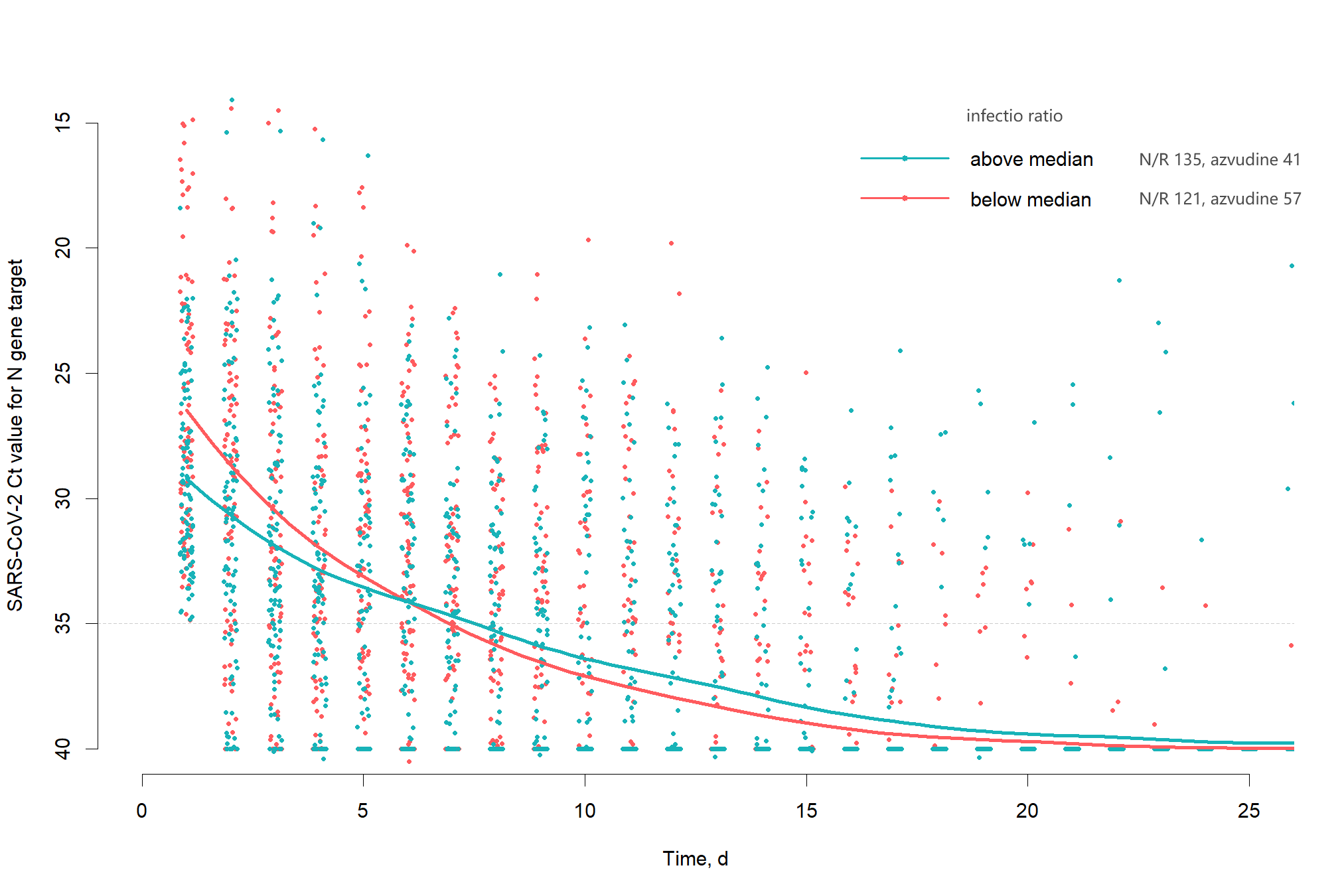

Supplement: Supplementary file 3 — Table S1. Characteristics of hospitalized patients receiving nirmatrelvir/ritonavir or receiving azvudine. Table S2. Stratified analysis of the impact of different baseline characteristics on patient prognosis. Table S3. Analysis of follow‐up durations and number of patients triggering endpoint events in the two groups. Figure S1. Visualization of artificial intelligence imaging analysis results for chest ct scans based on VB‐Net (United Imaging Intelligence). Figure S2. Scatter plot of serial cycle threshold values for all patients treated with nirmatrelvir/ritonavir and azvudine (including patients did not undergo chest CT scans). Figure S3. KM curves for the two groups based on the time to first negative test result. Figure S4. AI‐based radiological analysis in hospitalized patients treated with nirmatrelvir/ritonavir or azvudine after propensity score matching. Figure S5. KM curves for nirmatrelvir/ritonavir and azvudine after infection ratio incorporate into covariates of propensity score matching. Figure S6. KM curves for nirmatrelvir/ritonavir and azvudine in patients with infection ratio high (> 9.2) and infection ratio low (≤ 9.2). Figure S7. Scatter plot of serial cycle threshold values for all patients with infection ratio high (> 9.2) and infection ratio low (≤ 9.2). [file IRV-18-e70006-s001.docx]
